# Significance of Catecholamine Biosynthetic/Metabolic Pathway in SARS-CoV-2 Infection and COVID-19 Severity

**DOI:** 10.3390/cells12010012

**Published:** 2022-12-20

**Authors:** George Mpekoulis, Katerina I. Kalliampakou, Raphaela S. Milona, Despoina Lagou, Anastasios Ioannidis, Edison Jahaj, Christos T. Chasapis, Dionysis Kefallinos, Ioannis Karakasiliotis, Anastasia Kotanidou, Stylianos Chatzipanagiotou, Dido Vassilacopoulou, Alice G. Vassiliou, Emmanouil Angelakis, Niki Vassilaki

**Affiliations:** 1 Laboratory of Molecular Virology, Hellenic Pasteur Institute, 11521 Athens, Greece; 2 Department of Nursing, University of Peloponnese, 23100 Sparti, Greece; 3 GP Livanos and M Simou Laboratories, First Department of Critical Care Medicine & Pulmonary Services, National and Kapodistrian University of Athens Medical School, Evangelismos Hospital, 10676 Athens, Greece; 4 Institute of Chemical Biology, National Hellenic Research Foundation, 11635 Athens, Greece; 5 School of Electrical Engineering and Computer Science, National Technical University of Athens, 9 Iroon Polytechniou Street, Zografou, 15773 Athens, Greece; 6 Laboratory of Biology, Department of Medicine, Democritus University of Thrace, 68100 Alexandroupolis, Greece; 7 Department of Medical Biopathology, Medical School, University of Athens, Eginition Hospital, 11528 Athens, Greece; 8 Section of Biochemistry and Molecular Biology, Faculty of Biology, National and Kapodistrian University of Athens, 15772 Athens, Greece; 9 Department of Diagnostics, Hellenic Pasteur Institute, Athens 11521, Greece; 10 Aix Marseille University, IRD, IHU Méditerranée Infection, VITROME, 13005 Marseille, France

**Keywords:** SARS-CoV-2, catecholamine pathway, L-Dopa decarboxylase, dopamine, disease severity

## Abstract

The SARS-CoV-2 infection was previously associated with the expression of the dopamine biosynthetic enzyme L-Dopa decarboxylase (DDC). Specifically, a negative correlation was detected between DDC mRNA and SARS-CoV-2 RNA levels in in vitro infected epithelial cells and the nasopharyngeal tissue of COVID-19 patients with mild/no symptoms. However, *DDC,* among other genes related to both *DDC* expression and SARS-CoV-2-infection (*ACE2*, *dACE2*, *EPO*), was upregulated in these patients, possibly attributed to an orchestrated host antiviral response. Herein, by comparing *DDC* expression in the nasopharyngeal swab samples of severe/critical to mild COVID-19 cases, we showed a 20 mean-fold reduction, highlighting the importance of the expression of this gene as a potential marker of COVID-19 severity. Moreover, we identified an association of SARS-CoV-2 infection with the expression of key catecholamine biosynthesis/metabolism-related genes, in whole blood samples from hospitalized patients and in cultured cells. Specifically, viral infection downregulated the biosynthetic part of the dopamine pathway (reduction in *DDC* expression up to 7.5 mean-fold), while enhanced the catabolizing part (increase in monoamine oxidases A and B expression up to 15 and 10 mean-fold, respectively) in vivo, irrespectively of the presence of comorbidities. In accordance, dopamine levels in the sera of severe cases were reduced (up to 3.8 mean-fold). Additionally, a moderate positive correlation between DDC and MAOA mRNA levels (r = 0.527, *p* < 00001) in the blood was identified upon SARS-CoV-2-infection. These observations were consistent to the gene expression data from SARS-CoV-2-infected Vero E6 and A549 epithelial cells. Furthermore, L-Dopa or dopamine treatment of infected cells attenuated the virus-derived cytopathic effect by 55% and 59%, respectively. The SARS-CoV-2 mediated suppression of dopamine biosynthesis in cell culture was, at least in part, attributed to hypoxia-like conditions triggered by viral infection. These findings suggest that L-Dopa/dopamine intake may have a preventive or therapeutic value for COVID-19 patients.

## 1. Introduction

Severe acute respiratory syndrome coronavirus 2 (SARS-CoV-2), that emerged in Wuhan in December 2019 is a positive sense, single-stranded, RNA virus, which may cause severe respiratory infections in humans [1,2]. Symptoms of the disease provoked by the virus (coronavirus disease 2019 or COVID-19) may typically include fever, cough, myalgia or fatigue, sputum, headache, hemoptysis, and diarrhea, as well as dysregulation of the nervous system (loss of smell and taste, consciousness impairment, parkinsonism), and in the most severe cases may also lead to respiratory failure [3,4,5]. Virus infection activates the host immune response, causes dysfunction of the lung epithelial–endothelial barrier, and produces lung and airway hypoxia resulting in oxidative stress [6,7,8]. The induction of systemic hyper-inflammation increases the severity of the disease [9,10,11] and elevates the fatality rate even among younger COVID-19 patients [12].

The SARS-CoV-2 virion enters human cells through the binding of the Spike protein (S) to the angiotensin converting enzyme 2 (ACE2) exopeptidase of the cell membrane that acts as the receptor of the virus [13]. The ACE2 levels are elevated by several factors (such as sex and age), and comorbidities such as cardiovascular diseases, obesity, and diabetes [14], which are known risk factors of the worse clinical manifestations observed in COVID-19 patients [15,16]. Moreover, an N-terminally truncated isoform of ACE2, termed deltaACE2 (dACE2), has been identified as a product of an interferon-stimulated gene (ISG) [17,18]. The dACE2 isoform does not bind to the S protein and is upregulated by SARS-CoV-2 infection in COVID-19 patients [19,20] and in human cell lines and organoids [17]. Expression of the *dACE2* gene is higher in airway epithelia and bile duct epithelia in the liver as compared to *ACE2*, which is generally expressed in human kidney, heart, lung, liver, and vasculature [21,22]. The gene *dACE2* has been suggested to be partially responsible for the elevated expression of the *ACE2* locus reported in viral infections, including SARS-CoV-2 [17,18]. This was verified by our previous data from nasopharyngeal swab samples of SARS-CoV-2 infected subjects with mild or no symptoms [19], consistently with the induction of other ISGs in COVID-19 infection [20,23,24]. Specifically, we observed significantly higher induction in *dACE2* compared to *ACE2* in SARS-CoV-2 infected subjects [19]. Furthermore, in our study, the expression of *dACE2* seemed to have an inverse relationship with the viral load in these patients, while the expression of *ACE2* had a positive association [19]. In addition, several groups have demonstrated elevated levels of ACE2 enzymatic activity in the serum of COVID-19 patients [25,26,27].

Microarray meta-analysis of human tissues has unveiled that the most statistically important co-expressed gene with the *ACE2* locus, is L-Dopa decarboxylase (*DDC*) [28]. The DDC protein catalyzes dopamine and serotonin biosynthesis, which possess immunomodulatory, apart from their neuro-modulatory, function [29,30,31,32]. The *DDC* gene has also been related to the regulation of cell proliferation and apoptosis [33,34]. We have previously shown that it is the expression of *dACE2* that positively correlates with the expression of *DDC* in human epithelial cells of the nasal cavity, while no correlation has been detected between the expression of *DDC* and *ACE2*, regardless of SARS-CoV-2 infection [19]. In intestinal organoids infected by SARS-CoV-2, the *ACE2* locus has been found to be co-expressed with other genes encoding enzymes/proteins involved in the metabolism and transport of dopamine and trace amines, in addition to *DDC* [35]. These genes include the monoamine oxidase A (*MAOA*), the monoamine oxidase B (*MAOB*), and several transporter-encoding genes of the solute carrier family (*SLC7A9*, *SLC3A1*, *SLC6A19* and *SLC3A2*). Moreover, we have reported that *DDC* expression was enhanced in the nasopharyngeal tissue of COVID-19 patients with mild or no symptoms, whereas we detected a negative correlation between *DDC* expression and SARS-CoV-2 RNA levels, also verified in infected Vero E6 cells [19]. In later in vitro studies, Limanaqi et al. [36] observed that the exogenous administration of dopamine/dopamine agonists in epithelial cells negatively affected SARS-CoV-2 replication, in association with a reduction in D1 and D2 dopamine receptors’ mRNA and protein levels, and concurrently upregulated Type-I IFNs and ISGs. The effects of dopamine on viral replication and on D2DRs expression were reproduced by Type-I IFN administration, which in parallel upregulated DDC expression [36]. Our group also showed that the expression and activity of DDC, as well as the treatment of cells with dopamine, reduce the replication of other RNA viruses, including hepatitis C (HCV) and dengue (DENV) viruses [37,38,39]. Complementing our results, an interaction of SARS-CoV-2 viral proteins with the catecholamine degradation enzymes MAOA and catechol-O-methyltransferase (COMT) [40,41,42,43,44], has also been observed. Additionally, the presence of COMT has been deemed as necessary for SARS-CoV-2 infection [45].

It is known that the expression of *DDC* and *ACE2* can also be regulated by hypoxia as well, through mechanisms dependent on hypoxia-inducible factors (HIFs) [39,46,47,48,49,50,51]. Interestingly, based on the observed upregulation of known hypoxia target genes, such as *GLUT1* and *VEGFA* [52,53], we have indicated that the virus infection triggers hypoxia-like conditions in Vero E6 and A549 cells upon SARS-CoV-2 infection [19], and have suggested hypoxia signaling as a mechanism of *DDC* and *dACE2* co-regulation during infection. Moreover, the expression of *GLUT1* in relation to that of other genes, in the blood samples of COVID-19 patients, may be useful in predicting disease severity [54]. Additionally, *VEGF* expression has been shown to be increased also in infected subjects [53,55]. Indeed, hypoxia or silent hypoxia, that are common clinical manifestations of COVID-19, have also been detected in inflammatory sites of the injured lung tissue in these patients [8,56].

The previously observed differentiated regulation of *DDC*, *ACE2*, *dACE2* and *EPO* gene expression in the in vitro infection studies and the nasopharyngeal swab samples of SARS-CoV-2 infected subjects with mild or no symptoms, could be related to an antiviral response of the host in an attempt to restrict the propagation of SARS-CoV-2. Herein, to clarify this discrepancy and to address the implication of the catecholamine pathway in SARS-CoV-2 infection and disease severity, we studied the above-mentioned genes, and other genes involved in the catecholamine pathway in samples from patients and cultured cells infected with SARS-CoV-2. Specifically, we included nasopharyngeal swabs from COVID-19 patients with severe/critical manifestations and compared them to samples from patients with mild/no symptoms of the disease. Moreover, we analyzed the expression of genes related to the catecholamine pathway in whole blood samples of severe cases, and in SARS-CoV-2 infected Vero E6 epithelial cells. Serum dopamine levels in COVID-19 patients were also examined. Finally, we determined the influence of the DDC enzymatic substrate (L-Dopa), and its product (dopamine) on the virus-derived cytopathic effect in cell culture. Altogether, the prognostic and therapeutic values of the catecholamine pathway in COVID-19 disease were determined.

## 2. Materials and Methods

### 2.1. Human Samples

Nasopharyngeal swab samples and whole blood samples were obtained from patients in Greek hospitals or from healthy subjects. All categories of samples used in this study were totally independent and there was no intersection between them.

Nasopharyngeal swab samples were obtained upon routine diagnosis from adult patients tested positive for SARS-CoV-2, with mild or no symptoms (Mild CoV-2 group) or hospitalized in the intensive care unit (ICU) with severe/critical clinical symptoms (Severe CoV-2 group). Samples from subjects with a negative SARS-CoV-2 RT-PCR test constituted the control group for swab tissues (Negative group). All nasopharyngeal swab samples were obtained from the biobank of Eginition Hospital (Athens, Greece).

Whole blood samples were collected, with appropriate and standardized procedures, from COVID-19 patients who required admission to the COVID-19 specialized ward (Ward/CoV-2 group) or the ICU (ICU/CoV-2 group) of the Evangelismos General Hospital. Diagnosis of the viral infection was performed with RT-PCR in the nasopharyngeal swab samples. As control groups, samples from healthy donors (Healthy group), and from patients who were admitted to the ICU but tested negative for SARS-CoV-2 infection (ICU/non-CoV-2 group) were used. The ICU/non-CoV-2 group consisted mainly by subjects who were admitted to the ICU due to head injuries or multi-trauma patients (~70% of total cases).

Dopamine quantitation was performed in serum samples of the COVID-19 patients who required admission to the COVID-19 specialized ward (Ward/CoV-2 group), or in the ICU of the Evangelismos General Hospital. The COVID-19 patients who were hospitalized in the ICU were either treated with dexamethasone (ICU/CoV2 + Dexa group) or not (ICU/CoV-2 group). Serum samples from healthy donors (Healthy group), and subjects not infected with SARS-CoV-2 who were admitted in the ICU (ICU/non-CoV-2 group), were used as controls.

### 2.2. Cell and Virus Culture

Vero E6 cells, originally obtained from ATCC#CRL-1586, were cultured in Dulbecco’s modified minimal essential medium (DMEM) (Invitrogen, Waltham, MA, USA) with high glycose (25 mM) after supplementation with penicillin (100 U/mL), streptomycin (100 μg/mL) and fetal calf serum (10% *v*/*v*), at 37 °C and 5% CO_2_. A COVID-19 patient’s oropharyngeal swab sample was used to obtain the SARS-CoV-2 (isolate 30-287, lineage B1) virus [57]. Vero E6 cells at full confluency were inoculated with the isolated lineage and 4 days post-infection, the media were collected, and the virus was passaged once again in Vero E6 cells. Three days post-infection, the cultured cells were examined for a clear cytopathic effect (CPE), and the virus culture was harvested and stored at −80 °C after filtration (through 0.45μm filters). For titration of the propagated virus, Vero E6 cells were seeded in 96-well plates, inoculated with serial dilutions of the supernatant collected above and further cultured for 96 h at 37 °C. Inverted phase contrast microscopy was carried out to monitor the cytopathic effect (CPE), while the Reed and Muench method was used to determine the TCID_50_ [58].

### 2.3. Infection Studies

Vero E6 cells were infected with the SARS-CoV-2 virus (multiplicity of infection, MOI = 0.1), in 6-well plates. As a control, mock-infected cells were used. The virus inoculum was replaced with culture medium 1 h post-inoculation. Total RNA was extracted from cells, 24 and 48 h post-infection using NucleoZOL (Macherey–Nagel), according to the manufacturer’s instructions.

### 2.4. Total RNA Extraction from Human Samples

Whole blood samples were stored in tubes with RNA stabilizing solution (Applied Biosystems (Waltham, MA, USA), Life Technologies Corporation (Carlsbad, CA, USA)). Total RNA was extracted using the Tempus spin isolation kit (Applied Biosystems) according to the manufacturer’s instructions.

Nasopharyngeal swab samples were collected in appropriate tubes containing 2 mL preservation fluid. Total RNA was extracted using the MagNA Pure LC Total Nucleic Acid Isolation Kit and the MagNa Pure LC 2.0 automated nucleic acid purifier (Roche, Basel, Switzerland), according to the manufacturer’s instructions.

### 2.5. RNA Quantification by Reverse Transcription-Quantitative PCR (RT-qPCR)

Total RNA was reverse transcribed to cDNA using the reverse transcriptase of the Moloney murine leukemia virus (Promega, Madison, WI, USA) as recommended by the manufacturer, using oligo d(T)18 primers (New England Biolabs, Ipswich, MA, USA) and the recombinant ribonuclease inhibitor (Takara, San Jose, CA, USA). After cDNA synthesis, real-time quantitative PCR (qPCR) was performed for each target gene, using specific primer sets (Table 1), previously validated [18,33,59,60,61], and the SYBR-Green Luna Universal qPCR Master Mix (New England Biolabs). Two different primer sets were used for the detection of *DDC* expression, recognizing the majority of DDC mRNA isoforms (variants: 1, 2, 4, 6 and 7) [29,62]. The first pair binds to the first (forward primer) and second (reverse primer) exons and was used for DDC quantification in the nasopharyngeal swab samples. The second primer set, binds to the second (forward primer) and third (reverse primer) exons and was used in the whole blood samples. The mRNA levels of the house-keeping gene 14-3-3-zeta polypeptide (*YWHAZ*) were used for normalization. The SARS-CoV-2 positive and negative samples were analyzed concurrently. All qPCR reactions were carried out in the VIIA7 platform (Applied Biosystems).

The SARS-CoV-2 RNA quantification was assessed by RT-qPCR using the LightMix Modular Sarbecovirus *E*-gene Kit for the viral envelope protein (E)-encoding gene quantification and the LightMix Modular SARS-CoV-2 (COVID-19) RdRP Kit for the quantification of the *RdRp* gene, according to the manufacturer’s instructions. Myostatin (MSTN) mRNA levels were quantified, using the LightMix ModularDx Kit MSTN Extraction Control kit (Roche), and served as a reference. The SARS-CoV-2 positive and negative samples were analyzed in parallel.

The cell gene expression data were analyzed utilizing the relative quantification method 2^−∆∆Ct^ [63]. The expression of the house-keeping gene 14-3-3-zeta polypeptide (*YWHAZ*) was used for the cell genes’ expression normalization.

### 2.6. Elisa Assays

Dopamine levels were quantified in the sera of the following groups: Ward/CoV-2, ICU/CoV-2 + Dexa, ICU/CoV-2, Healthy and ICU/non-CoV-2, using the Dopamine ELISA kit (IBL International, Hamburg, Germany), according to the manufacturer’s instructions. The detection limit of this assay is 4 pg/mL and the intra- and inter-assay coefficients of variability are 11% and 16.3%, respectively. Hence, this assay provides high specificity, good accuracy, and precision [64].

### 2.7. Cytopathic Effect Quantitation (CPE)

To record the effect of L-Dopa and dopamine on SARS-CoV-2- infection in vitro, Vero E6 cells were seeded in a 24-well plate (1.8 × 10^5^ cells/well) and were inoculated with the produced viral stock (Section 2.2) for 1 h. Subsequently, the virus containing medium was replaced with DMEM complete supplemented or not with L-Dopa or dopamine in a non-cytotoxic concentration (50 μΜ). Seventy-two hours post-infection, the cells (10 wells per condition) were observed using an inverted microscope with phase contrast for the detection of SARS-CoV-2 induced cytopathic effect (CPE). The CPE foci in the L-Dopa or dopamine treated cells were counted and compared to those in the non-treated cells.

### 2.8. SARS-CoV-2–Human Protein–Protein Interactions (PPIs)

The experimentally determined protein-protein interactions (PPIs) between SARS-CoV-2 and human host proteins were retrieved from the publication of the respective high-throughput yeast-2-hybrid experiments in which all pairwise combinations of 28 SARS-CoV-2 proteins against ~16,000 human proteins were systematically tested [65] and the available literature [41,42,43,45,66]. The experientially derived interconnectivity of human host factors was mined from the PICKLE (Protein InteraCtion KnowLedgebasE) meta-database [67].

### 2.9. Chemicals

L-3,4-dihydroxyphenylalanine (L-Dopa), and dopamine (DA), were obtained from Sigma–Aldrich (St. Louis, MO, USA). Both compounds were diluted in dimethyl sulfoxide (DMSO) in a 100 mM stock-solution and were preserved at −20 °C.

### 2.10. Measurement of Cellular ATP Content

For measurement of the intracellular ATP content, Vero E6 cells were seeded in a 96-well plate and were subsequently treated or not with three consecutive dilutions of L-Dopa or dopamine (25, 50 and 100 μΜ). At 72-h post-inoculation, the cells were lysed, and the intracellular ATP levels were evaluated using the ViaLight HS BioAssay kit (Lonza, Basel, Switzerland), according to the manufacturer’s instructions, in a GloMax 20/20 single-tube luminometer (Promega Corporation, Madison, WI, USA) for 1 s. The total amount of protein was used for normalization. The maximum non-cytotoxic concentrations were chosen to treat the infected cells.

### 2.11. Statistical Analysis

Normality tests were conducted to test the distribution of variables (parametric or non-parametric) to select the appropriate statistical analysis tool each time. The statistical analysis of the comparison of gene expression, dopamine levels and age (continuous parameters) between COVID-19 patients and control groups was performed with either ordinary One Way ANOVA for parametric values, or Kruskal–Wallis test for the non-parametric values, followed by the appropriate post-hoc analysis. Multiple comparisons between the different groups were conducted with Tukey’s test for ordinary ANOVA, and with Dunn’s test for the non-parametric variables. The diagnostic accuracy of gene expression was evaluated with regression analysis (ROC). The area under curve (AUC), and the cut-off value based on Youden’s index were calculated for each gene. The student’s *t*-test (unpaired) or the Mann–Whitney U test were performed to compare the gene expression between men and women. Additionally, sex distribution among the studied groups was determined with the chi-squared test. Pearson’s or Spearman’s correlation coefficient (*r*) was performed to evaluate the correlation of (a) the expression levels of the various genes studied, (b) the expression level of each studied gene and the viral RNA, (c) the expression level of each studied gene and age (continuous variable), and the clinical measurements of the patients. The comparison of COVID-19 patients’ dopamine levels with those of the control groups, in the presence of absence of comorbidities or mechanical ventilation was performed with Two Way ANOVA. GraphPad Prism 9.0 (GraphPad Software, Inc., San Diego, CA, USA) was used to perform all statistical analyses, and *p* < 0.05 (two-tailed) was considered statistically significant.

## 3. Results

We have previously shown that *DDC* mRNA levels were upregulated in the nasopharyngeal tissue of COVID-19 patients with mild or no symptoms (Mild CoV-2) compared to subjects not infected with SARS-CoV-2 (Negative) [19]. We also demonstrated that the regulation of this gene is SARS-CoV-2 infection-specific, as its mRNA levels were not altered in patients infected with another respiratory virus, compared to the Negative group. On the other hand, *DDC* expression negatively correlated with the SARS-CoV-2 viral load in the nasopharyngeal tissue of the same patients. Similarly, *DDC* was downregulated in cell culture upon SARS-CoV-2 infection. Additionally, the *DDC* gene displayed substantial co-expression with the *ACE2* locus [28], and specifically with the mRNA of the IFN-inducible *ACE2* truncated isoform *dACE2* [17,18], but not with the SARS-CoV-2 receptor-encoding *ACE2* mRNA, despite that both *ACE2* and *dACE2* genes were upregulated in these patients.

### 3.1. L-Dopa Decarboxylase as a COVID-19 Severity Marker in Nasopharyngeal Swab Samples

To elucidate whether the expression of *DDC*, *ACE2*, *dACE2*, or *EPO* are also altered in the nasopharyngeal tissue of COVID-19 patients with severe symptoms, we examined 21 samples obtained by SARS-CoV-2 infected patients admitted to the ICU and compared them to the Mild CoV-2 and Negative groups. The median age of the Severe CoV-2, Mild CoV-2, and Negative groups was 64 years (IQR = 61–75), 38 years (IQR = 31–52), and 47 years (IQR = 34–68), respectively (Table 2).

**Figure 1:**
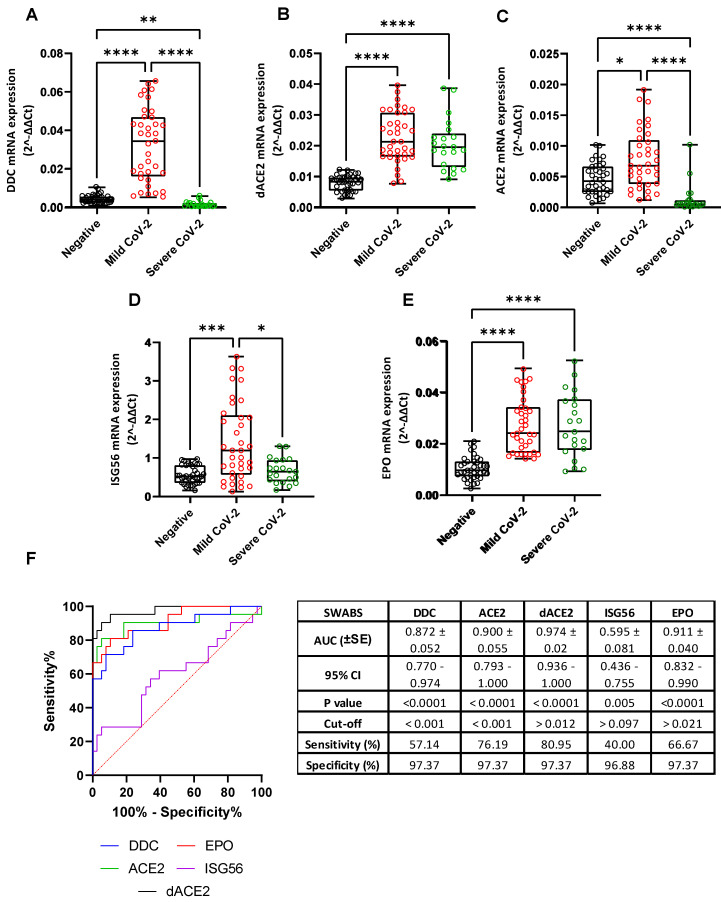
Comparison of *DDC* (**A**), *dACE2* (**B**), *ACE2* (**C**), *ISG56* (**D**), and *EPO* (**E**) expression in the nasopharyngeal swab samples of SARS-CoV-2 positive subjects with mild (Mild CoV-2) or severe (Severe CoV-2) symptoms and SARS-CoV-2 negative subjects (Negative). (**F**) ROC curve analysis of the expression of the aforementioned-genes in the Severe CoV-2 cohort. (**A**–**E**) Data are shown as box plots and dots; the line in the middle corresponds to the median value; box edges, 25th to 75th centiles; whiskers, range of values. The *p*-values were calculated with the Kruskal–Wallis test. Dunn’s test was performed for multiple comparisons. ROC curves were generated, and AUC, 95% CI, *p*-values, and cut-off points with their specificity and sensitivity were reported. The red dotted line denotes perfect chance (positive likelihood ratio = sensitivity/(1-specificity). * *p* < 0.05, ** *p* < 0.01, *** *p* < 0.001, **** *p* < 0.0001 vs. Negative or Mild CoV-2.

***DDC* mRNA expression:** Regarding the expression of *DDC* in the nasopharyngeal swabs, we observed a statistically significant reduction of 20 mean-fold (Table S1, *p* < 0.0001) in the Severe CoV-2 group (median = 0.0007, IQR = 0.0004–0.002) compared to the Mild CoV-2 group (median = 0.034, IQR = 0.016–0.047) (Figure 1A). Surprisingly, the mRNA levels of *DDC* in the Severe CoV-2 cohort were even lower (2 mean-fold, *p* < 0.0001) compared to the Negative control group (median = 0.0041, IQR = 0.0026–0.0057). In addition, *DDC* expression in this tissue was characterized by high diagnostic accuracy, as determined by the ROC curve analysis, with an AUC of 0.872 (95% confidence interval–CI = 0.770–0.974). The optimal cut-off value was 0.001, with a sensitivity of 57.14% and a specificity of 97.37%. In the Severe CoV-2 group, no statistically significant change in the expression of *DDC* was observed between men and women (Figure S1A), nor a correlation with age was found (Figure S2A), in accordance with our previous results in Negative and Mild CoV-2 subjects [19].

***dACE2* and *ACE2* mRNA levels**: The *dACE2* mRNA levels in the nasopharyngeal tissue of Severe CoV-2 subjects (median = 0.020, IQR = 0.013–0.024) were higher up to 2.5 mean-fold (Table S1, *p* < 0.0001) compared to the Negative group (median = 0.008, IQR = 0.006–0.01), while there was no difference compared to the Mild CoV-2 patients (median = 0.021, IQR = 0.017–0.031) (Figure 1Β). On the contrary, we detected diminished levels of *ACE2* mRNA (median = 0.00051, IQR = 0.0003–0.0012) in the Severe CoV-2 group compared to those obtained from the Negative subjects (median = 0.004, IQR = 0.003–0.007), and the Mild CoV-2 patients (median = 0.007, IQR = 0.004–0.011) (*p* < 0.0001) (Figure 1C). Moreover, in the Severe CoV-2 group, the ROC curve analysis revealed a high diagnostic accuracy of the expression of both *ACE2* (AUC = 0.900, 95% CI = 0.793–1.000) and *dACE2* (AUC = 0.974, 95% CI = 0.936–1.000). The optimal cut-off value for *ACE2* expression was at 0.001, with a sensitivity and a specificity of 76.19% and 97.37%, respectively. Regarding *dACE2* expression, the cut-off point value was 0.012, with 80.95% sensitivity and 97.37% specificity. Finally, *ACE2* and *dACE2* expression did not correlate with either sex (Figure S1B,C) or age (Figure S2B,C). Taking into account that *dACE2* is considered to be an ISG [17,18], we also examined the expression of *ISG56*, which is most widely studied gene, compared to *dACE2*. The mRNA levels of *ISG56* in the Severe CoV-2 group (median = 0.649, IQR = 0.399–0.940) were slightly increased (0.2 mean-fold, *p* = 0.0413) as compared to the ones of the Negative cohort (median = 0.521, IQR = 0.358–0.814), while they were lower (about 2 mean-fold, *p* = 0.0002) than in the Mild CoV-2 group (median = 1.194, IQR = 0.572–2.106) (Figure 1D). Furthermore, the ROC curve analysis of the expression of *ISG56* led to a much lower discriminating accuracy (AUC = 0.595, 95% CI = 0.436–0.755). At an optimal cut-off value of 0.097, the specificity and sensitivity were 96.88% and 40%, respectively. Finally, the mRNA levels of ISG56 in this tissue did not correlate with the patients’ demographic data (Figures S1D and S2D).

***EPO* mRNA levels**: We have previously shown upregulation of *EPO* expression in the nasopharyngeal swab samples of COVID-19 patients with mild symptoms [19]. Here, we determined EPO mRNA levels in the Severe CoV-2 cohort and compared them to the Negative and Mild CoV-2 groups. We observed that EPO mRNA levels were significantly upregulated (up to 2.7 mean-folds, *p* < 0.0001) in the Severe CoV-2 group (median = 0.025, IQR = 0.018–0.037) (Table S1) compared to the Negative one (median = 0.01, IQR = 0.007–0.013) (Figure 1E), whereas no significant difference was found between the Severe CoV-2 and the Mild CoV-2 (median = 0.025, IQR = 0.017–0.034) cohorts. In addition, the expression of *EPO* in the Severe CoV-2 patients was characterized by a high diagnostic accuracy, as determined by the ROC curve analysis (AUC = 0.911, 95% CI = 0.832–0.990), with a sensitivity of 66.67% and a specificity of 97.37%, at an optimal cut-off point of 0.021. The expression of *EPO* also did not correlate with either age or the sex of the patients (Figures S1E and S2E).

**Figure 2:**
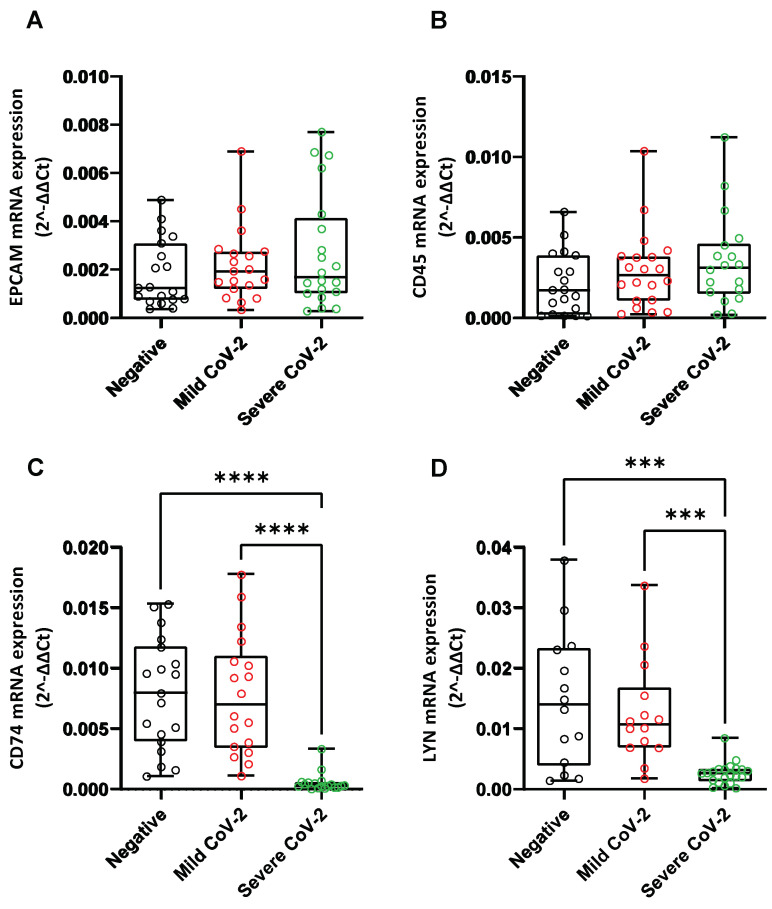
Comparison of the mRNA levels of *EPCAM* (**A**)**,** *CD45* (**B**), *CD74* (**C**) and *LYN* (**D**) in the nasopharyngeal swab samples of SARS-CoV-2 positive groups with mild (Mild CoV-2) or severe (Severe CoV-2) symptoms and SARS-CoV-2 negative cohort (Negative). Data are displayed as box plots and dots; the line in the middle corresponds to the median value; box edges, 25th to 75th centiles; whiskers, range of values. *p*-values were calculated with the non-parametric Kruskal–Wallis test. Dunn’s test was performed for multiple comparisons. *** *p* < 0.001, **** *p* < 0.0001.

Subsequently, we investigated the possibility that the observed changes in the expression of the above-mentioned genes in the nasopharyngeal swab samples of the Severe CoV-2 group, might be due to the fluctuation of cell populations related to inflammation. More specifically, we studied the expression of the epithelial cell marker *EPCAM* [68], and the nucleated hematopoietic cell marker *CD45* [69], which is also used as a marker of immune cell populations. Amongst the three studied groups, we did not observe any significant alterations in the mRNA levels of *EPCAM* (Figure 2A). The mRNA levels of *CD45* were slightly increased, albeit not significantly, in the Severe CoV-2 cohort compared to the Negative group (Figure 2B), which is in agreement with already published data [70]. These findings might possibly suggest that the observed alterations are not related to an enrichment of inflammation-related cell populations expressing these genes in the nasopharyngeal tissue of COVID-19 patients. In support of these results, concerning *DDC*, further analysis of the single cell gene expression data (RNA-sequencing) of Chua et al., derived from nasopharyngeal swabs of COVID-19 patients (available in Magellan COVID-19 Omics Explorer, https://digital.bihealth.org (accessed on 25 August 2022)), revealed that *DDC* is almost exclusively expressed in epithelial cells (secretory, ciliated and FOXN4) and not in immune cell populations [21].

As reports have shown that in severe cases of COVID-19 there is an impaired local intrinsic immunity against SARS-CoV-2 infection [70], we also examined the expression of *LYN* and *CD74* genes (markers of dendritic and Langerhans cells, respectively), which are normally expressed in the antigen-presenting cells) of the nasopharyngeal tissue. Interestingly, the mRNA levels of both CD74 and LYN were excessively lower in the nasopharyngeal swab samples of the Severe CoV-2 group as compared to the Mild CoV-2 and Negative groups (Figure 2C,D). The findings concerning *CD74* expression are in accordance with previous studies reporting that SARS-CoV-2 infection reduced the expression of *CD74* in the nose mucosal tissue [70].

### 3.2. Correlation of the mRNA Levels of DDC with dACE2 in the Nasopharyngeal Swabs of Severe CoV-2 Group

**Figure 3:**
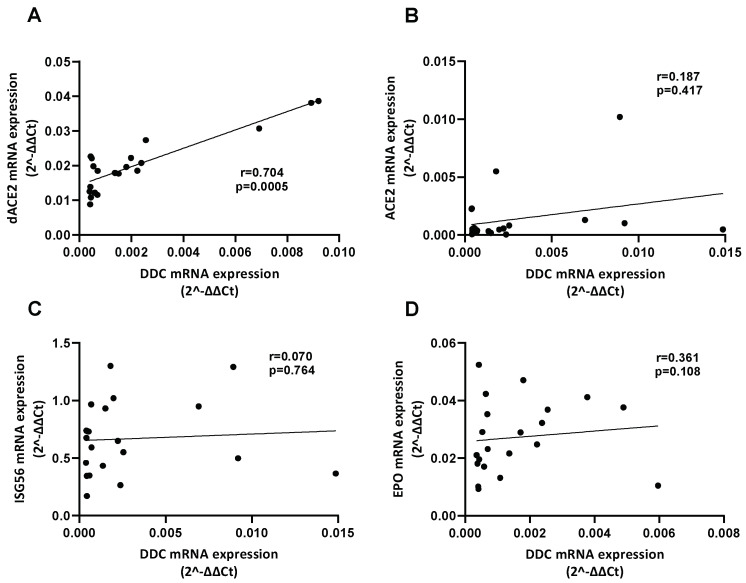
Correlation of the expression of *dACE2* (**A**), *ACE2* (**B**), *ISG56* (**C**) and *EPO* (**D**) with *DDC* in the nasopharyngeal swab samples of COVID-19 patients with severe symptoms (Severe CoV-2)**.** Data are presented as XY scatter plots with fitted linear regression lines. Pearson’s or Spearman’s correlation coefficient (r) and *p*-values (p) were calculated.

The previously observed correlation between the mRNA levels of *DDC* and *dACE2* in the SARS-CoV-2 infected subjects with mild or no symptoms [19], prompted us to examine this relationship in more severe cases of the disease. In the present study, we observed a strong positive correlation between *DDC* and *dACE2* expression in the Severe CoV-2 group (r = 0.704, *p* = 0.0005, Figure 3A), as opposed to a weaker correlation previously exhibited in the Negative group [19]. On the contrary, no correlation was detected between the mRNA levels of *DDC* and *ACE2*, *ISG56* or *EPO* (Figure 3B–D). Moreover, the expression of *dACE2* did not correlate with *ISG56*, *ACE2*, or *EPO* in this tissue (Figure S3A–C). Furthermore, because we previously detected a strong negative correlation of *DDC* and *dACE2* mRNA levels with the SARS-CoV-2 viral load in the nasopharyngeal tissue of mild COVID-19 cases [19], we also examined the correlation of these genes with SARS-CoV-2 titers in the Severe CoV-2 cohort. Overall, our results showed that among the genes studied, only the expression of *EPO* negatively correlated with SARS-CoV-2 viral load levels (r = −0.461, *p* = 0.353) (Figure S4). Interestingly, we detected significantly lower viral load levels in the Severe CoV-2 cohort (*p* = 0.0014) compared to the Mild CoV-2 group, possibly due to higher activation of the host innate immunity in the severe cases, restricting the replication of the virus [11] (Figure S5).

### 3.3. Reduced DDC and dACE2 Expression in Whole Blood Samples of Hospitalized COVID-19 Patients

**Figure 4:**
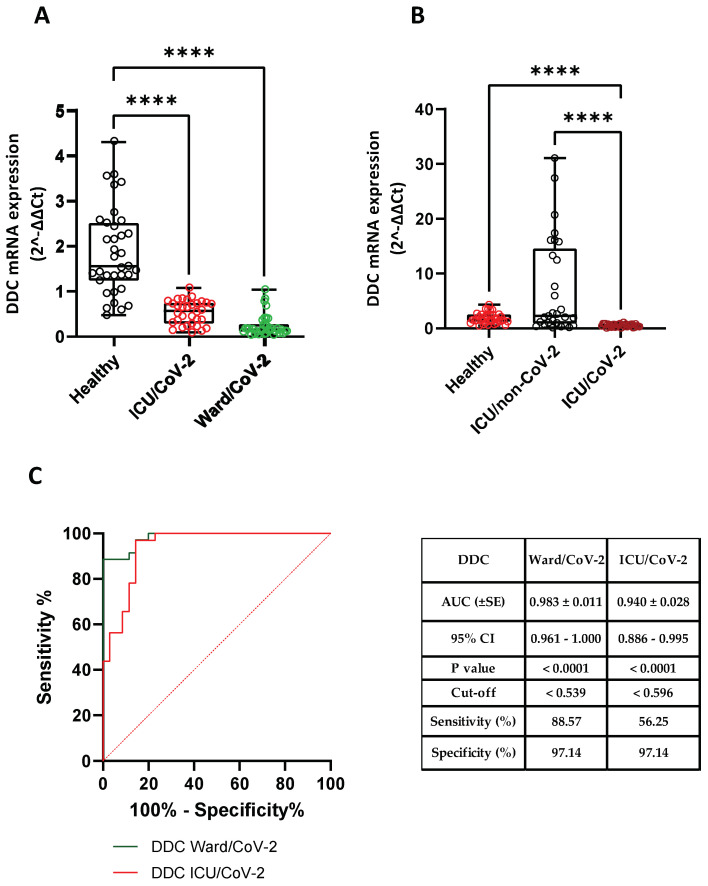
Comparison of the mRNA levels (mean 2^−ΔΔCt^) of *DDC* in whole blood samples in: (**A**) COVID-19 patients hospitalized in the intensive care unit (ICU/CoV-2, *n* = 32) or the ward (Ward/CoV-2, *n* = 30) and healthy subjects (Healthy, *n* = 30) (**Β**) Healthy subjects and hospitalized patients in the ICU either infected with SARS-CoV-2 (ICU/CoV-2) or not infected (ICU/non-CoV-2, *n* = 30) (**C**) ROC curve analysis of *DDC* expression in the ICU/CoV-2 and Ward/CoV-2 patient cohorts. Data are displayed as box plots and dots; the line in the middle corresponds for the median value; box edges, 25th to 75th centiles; whiskers, range of values. *p*-values were calculated with the Kruskal–Wallis test. Dunn’s test was performed for multiple comparisons. ROC curves were generated, and AUC, 95% CI, *p*-values, and cut-off points with their specificity and sensitivity were reported. The red dotted line denotes perfect chance (positive likelihood ratio = sensitivity/(1-specificity) =1). **** *p* < 0.0001.

Next, we investigated whether the expression of *DDC* was reduced in whole blood samples of severe COVID-19 cases, which would reinforce our data derived from the nasopharyngeal tissue. For this purpose, we used two SARS-CoV-2 positive hospitalized cohorts, one admitted to a specialized ward (*n* = 35, Ward/CoV-2) and one hospitalized in the ICU (*n* = 32, ICU/CoV-2). Moreover, a group of SARS-CoV-2 negative patients admitted to the ICU (*n* = 30, ICU/non-CoV-2) and a group of healthy subjects (*n* = 35, Healthy) were used as negative controls. The characteristics of the aforementioned-groups are presented in Table 3 and Table S2. We observed that the expression of *DDC* was significantly decreased in both ICU/CoV-2 (median = 0.571, IQR = 0.295–0.747) and Ward/CoV-2 (median = 0.158, IQR = 0.115–0.279) patients compared to the Healthy subjects (median = 1.567, IQR = 1.244–2.519) (Figure 4A), with the greatest decrease shown in the Ward/CoV-2 group (7.5 mean-fold, *p* < 0.0001; Table S3). The discriminating accuracy determined for DDC mRNA levels was high for both ICU/CoV-2 (AUC = 0.940, 95% CI = 0.886–0.995) and Ward/CoV-2 (AUC = 0.982, 95% CI = 0.961–1.000) cohorts. The optimal cut-off point for Ward-CoV-2 was calculated at 0.539, with a sensitivity of 88.57% and a specificity of 97.14%. The respective cut-off value for ICU/CoV-2 was 0.596, with a sensitivity and a specificity of 56.25% and 97.14%, respectively. Additionally, the expression of *DDC* did not correlate with either the sex or the age of the patients (Figures S6A and S7A). Because our data (Section 3.1) suggested DDC as a potential disease severity marker in the nasopharyngeal tissue, we aimed to investigate whether the observed alterations of its expression in the blood are SARS-CoV-2 infection-specific or are due to ICU admission. For this purpose, we determined the mRNA levels of *DDC* in the ICU/non-CoV-2 patients, as a negative control, and compared them to the ICU/CoV-2 and Healthy groups. The mRNA levels of *DDC* in the ICU/non-CoV-2 cohort were significantly higher than the ICU/CoV-2 group and did not differ from the healthy subjects. This indicates that the reduction in *DDC* expression is specific to SARS-CoV-2 infection and is not associated to ICU admission.

**Figure 5:**
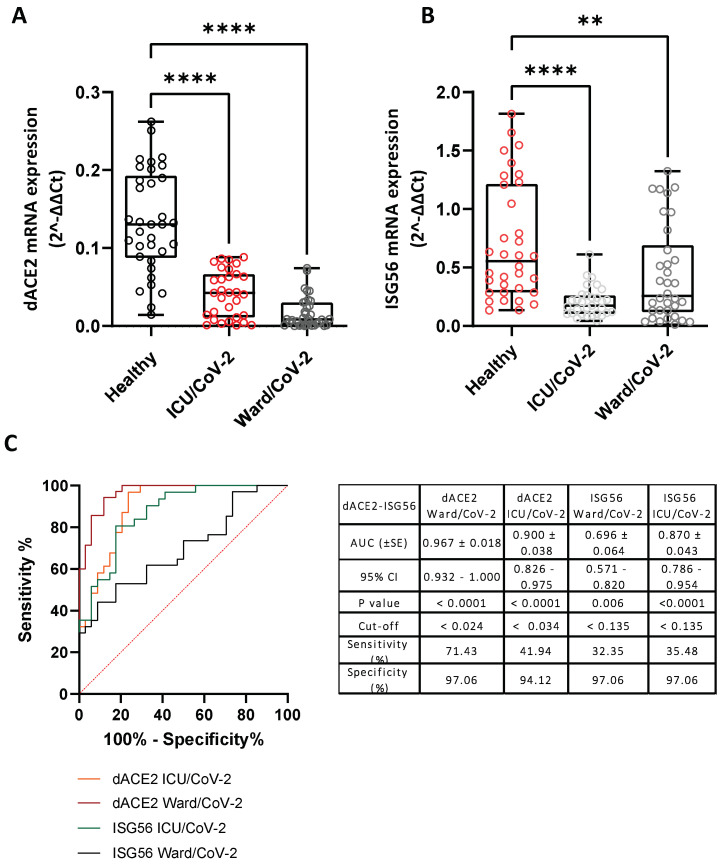
Comparison of the *dACE2* (**A**) and *ISG56* (**B**) expression in whole blood samples of healthy subjects (Healthy) and COVID-19 hospitalized patients (ICU/CoV-2, Ward/CoV-2 groups) and their ROC curve analysis (**C**). The mRNA expression data are presented as box plots and dots; line in the middle, median; box edges, 25th to 75th centiles; whiskers, range of values. *p*-values were calculated with the non-parametric Kruskal–Wallis test. Dunn’s test was carried out for multiple comparisons. ROC curves were generated and AUC, 95% CI and cut-off points with their specificity and sensitivity were calculated. *p*-value was ** *p* < 0.01, **** *p* < 0.0001.

Taking into account the correlation of *DDC* and *dACE2* expression that we observed in the nasopharyngeal tissues of both mild [19] and severe COVID-19 (present study) patients, we also evaluated *dACE2* mRNA levels, in the same whole blood samples that were used for *DDC* expression analysis. The *dACE2* mRNA levels were lower in both the ICU/CoV-2 (median = 0.042, IQR = 0.011–0.066) and the Ward/CoV-2 (median = 0.009, IQR = 0.001–0.030) cohorts, compared to the Healthy group (median = 0.130, IQR = 0.088–0.193), in accordance with the results obtained for *DDC.* The greatest decrease, of approximately 30 mean-fold (*p* < 0.0001, Table S3), was observed in the Ward/CoV-2 group (Figure 5A). In agreement to our findings on *dACE2* expression, a significant reduction in *ISG56* mRNA levels in both the Ward/CoV-2 (1.6 mean-fold, *p* = 0.0075) and ICU/CoV-2 (up to 3.4 mean-fold, *p* < 0.0001) cohorts was detected compared to the healthy subjects (Figure 5B). The ROC curve analysis of *dACE2* expression revealed a high diagnostic accuracy for both ICU/CoV-2 (AUC = 0.900, 95% CI = 0.826 to 0.975) and Ward/CoV-2 (AUC = 0.967, 95% CI = 0.932–1.000) patients. The optimal cut-off values for the two groups were 0.034 (sensitivity = 41.94% and specificity = 94.12%), and 0.024 (sensitivity = 71.42% and specificity = 97.06%), respectively. A lower diagnostic accuracy was detected for *ISG56* expression, especially in the Ward/CoV-2 group (AUC = 0.696, 95% CI = 0.571–0.820). The optimal cut-off points for the ICU/CoV-2 and Ward/CoV-2 patients were calculated at 0.135, with a sensitivity of 35.48% and a specificity of 97.06% for the ICU/CoV-2 cohort, and a sensitivity of 32.35% and a specificity of 97.06% for the Ward/CoV-2 one. Furthermore, there was no significant correlation between the expression of *dACE2* or *ISG56* with sex or age (Figures S6B,C and S7B,C).

To examine the possibility that the observed alterations in the expression of the above-mentioned genes might be due to a possible enrichment of inflammation-related cell populations in the blood samples of SARS-CoV-2 infected subjects exhibiting severe symptoms, the expression levels of the immune cell markers *CD45*, *CD74* and *LYN* were analyzed. In the blood, *CD45* is mainly considered a marker of myeloid and T-cells, while *CD74* and *LYN* constitute a marker of B-cells. A non-statistically significant decrease in CD45 expression was observed in the Ward/CoV-2 group, as compared to the healthy subjects. Previous studies have reported a reduction in the expression of this gene in COVID-19 patients’ blood samples [71]. We did not observe any significant alteration in the expressions of *CD74* and *LYN* in the whole blood of hospitalized COVID-19 patients compared to the Healthy group (Figure S8A–C).

### 3.4. Decreased Dopamine Levels in the Serum of Hospitalized COVID-19 Patients

**Figure 6:**
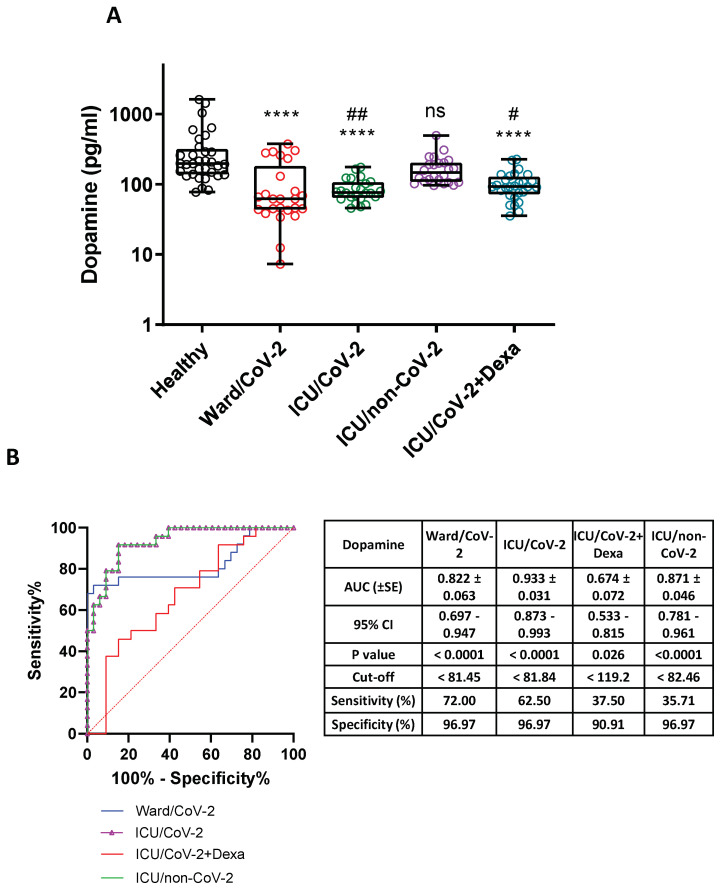
SARS-CoV-2 infection affects serum dopamine levels in COVID-19 hospitalized patients: (**A**) Comparison of dopamine absolute levels in healthy subjects (Healthy), COVID-19 patients hospitalized in the ward (Ward/CoV-2), ICU COVID-19 patients (ICU/CoV-2), SARS-CoV-2 negative ICU patients (ICU/non-CoV-2), and ICU COVID-19 patients who had received dexamethasone treatment (ICU/CoV-2 + Dexa). *p*-values were determined by the Kruskal–Wallis test. The Dunn’s test was performed for multiple comparisons. Data are displayed as box plots; the line in the middle, median; box edges, 25th to 75th centiles; whiskers, range of values. (**B**) ROC curve analysis of dopamine quantitation using the Healthy cohort as a control. ROC curves were generated, and AUC, 95% CI, *p*-values, and cut-off points with their specificity and sensitivity were reported. The red dotted line denotes perfect chance (positive likelihood ratio = sensitivity/(1-specificity) =1). **** *p* < 0.0001 vs. Healthy, ^#^ *p* = 0.029, ^##^ *p* = 0.0018 vs. ICU/non-CoV-2, ns: not significant vs. Healthy.

Based on our previous cell culture-derived data supporting that the infection of cells with SARS-CoV-2 reduced *DDC* expression [19] and the lower *DDC* mRNA levels detected in the blood of hospitalized SARS-CoV-2 infected subjects, we measured the dopamine levels in the serum of the COVID-19 severe/critical cases of the disease and compared them to two control groups. Specifically, we analyzed two SARS-CoV-2 infected cohorts; one group of patients hospitalized in the specialized COVID-19 ward (*n* = 27, Ward/CoV-2), and a second group that was admitted to the ICU (Table 4). The latter was further separated into patients receiving dexamethasone treatment (*n* = 29, ICU/CoV-2 + Dexa) or not (*n* = 33, ICU/CoV-2). The Healthy group (*n* = 33, Healthy), and a group of patients hospitalized in the ICU, tested negative for SARS-CoV-2 infection (*n* = 27, ICU/non-CoV-2), were used as controls. Decreased dopamine levels were observed in all cohorts of SARS-CoV-2 hospitalized patients, by about 3–3.8 mean-fold (*p* < 0.0001), as compared to the Healthy subjects (median = 197.8 pg/mL, IQR = 138.2–316.2 pg/mL) (Figure 6A). The most significant reduction was exhibited in the Ward/CoV-2 group (median = 62.22 pg/mL, IQR = 43.44–182.4 pg/mL). Interestingly, no significant alteration in the serum dopamine levels was observed between the Healthy and the ICU/non-CoV-2 groups. Moreover, lower dopamine levels were detected in both the ICU/CoV-2 (median = 76.43 pg/mL, IQR = 63.76–107.4 pg/mL) and the ICU/CoV-2 + Dexa (median = 93.06 pg/mL, IQR = 71.30–127.6 pg/mL) patients, compared to the ICU/non-CoV-2 subjects (median = 146.3 pg/mL, IQR = 107.9–202.4 pg/mL) (p_ICU/CoV-2_ = 0.002, p_ICU/CoV-2+Dexa_ = 0.029), indicating that our results on dopamine levels are SARS-CoV-2 infection-specific. The patients who were treated with dexamethasone (ICU/CoV-2 + Dexa) exhibited slightly higher levels of dopamine compared to the non-treated ones, which is in accordance with reported data showing that dexamethasone treatment positively affects the circulating dopamine [72]. Furthermore, the ROC curve analysis revealed a high diagnostic accuracy for the dopamine levels in all groups except from the ICU/CoV-2 + Dexa group (Figure 6B). All the above data combined, indicate that there is a possible negative association between serum dopamine levels and SARS-CoV-2 infection, independently of ICU admission. Therefore, the reduction in the mRNA levels of *DDC* in the whole blood samples of the hospitalized COVID-19 patients is accompanied by a significant decrease of serum dopamine levels, and both effects appeared to be SARS-CoV-2 infection-specific.

Next, we investigated whether the observed alterations in the serum dopamine levels in the COVID-19 patients could be related to underlying comorbidities. For this purpose, we tested the presence of underlying diseases as potential dopamine regulators in our three ICU cohorts. No significant correlation between dopamine levels and the underlying comorbidities was detected in the three patient groups (Figure S9A). Hypertension, the most common comorbidity in our patient cohorts, also had no significant impact on serum dopamine levels (Figure S9B). No effect was exerted by the application of mechanical ventilation in COVID-19 patients (Figure S9C). These data suggest that systemic dopamine is negatively influenced by SARS-CoV-2 infection independently of the presence of comorbidities.

### 3.5. Alteration of Catecholamine Pathway-Related Genes Expression in Whole Blood Samples of SARS-CoV-2-Infected Hospitalized Individuals

**Figure 7:**
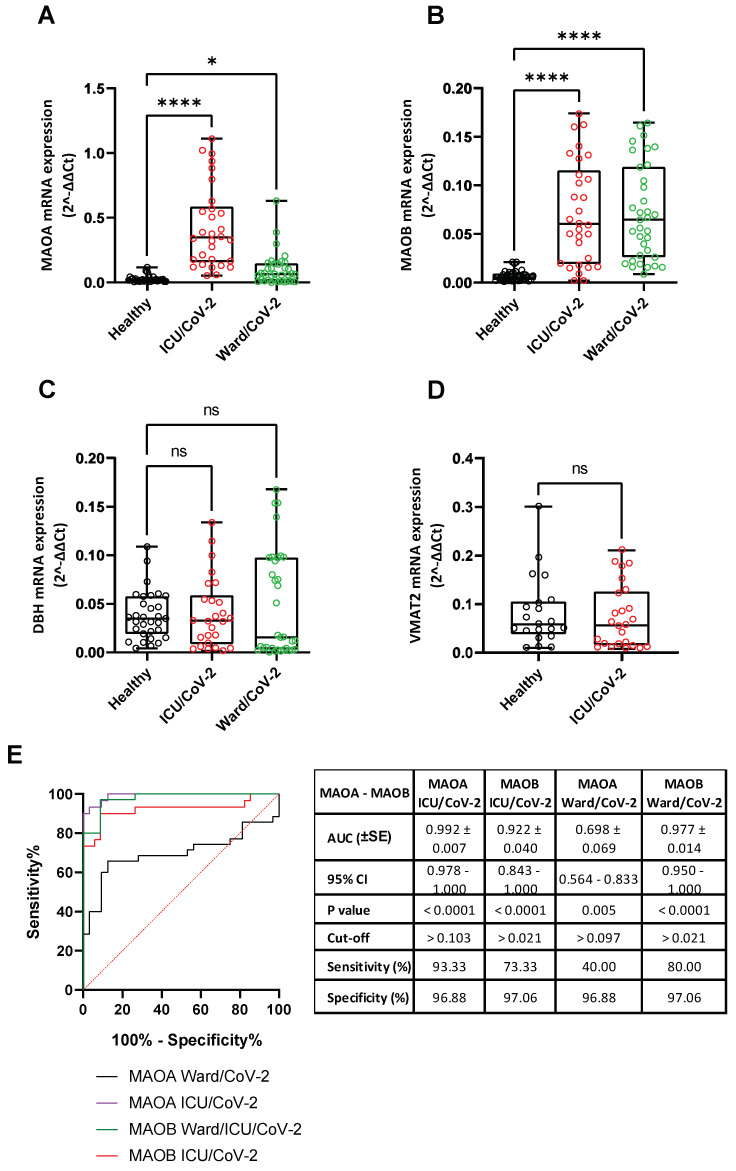
Comparison of the mRNA expression of (**A**) *MAOA*, (**B**) *MAOB*, (**C**) *DBH* and (**D**) *VMAT2* in hospitalized SARS-CoV-2 positive (ICU/CoV-2, Ward/CoV-2 groups) and healthy subjects (Healthy). The mRNA expression data are presented as box plots; line in the middle corresponds for the median value; box edges refer to the 25th and 75th centiles; whiskers, range of values. *p*-values were determined by the Kruskal–Wallis non-parametric test. The Dunns’s test was performed for multiple comparisons among the studied patient groups. * *p* < 0.05, **** *p* < 0.0001 vs. Healthy, ns: non-significant. The *p*-value for *VMAT-2* expression was evaluated with unpaired *t*-test. (**E**) ROC curve analysis for the aforementioned-genes expression. AUC, 95% CI, *p*-values, and cut-off points with their specificity and sensitivity were reported. The red dotted line denotes perfect chance (positive likelihood ratio = sensitivity/(1-specificity) =1).

Our aforementioned-results on whole blood DDC expression and serum dopamine levels in hospitalized COVID-19 patients indicated that the activity of the catecholamine pathway is affected by SARS-CoV-2 infection. To further investigate the association between SARS-CoV-2 infection and the function of the catecholamine pathway, the expression of other catecholamine pathway-related genes in blood was examined. These genes include *MAOA* and *MAOB*, which catalyze the oxidative deamination of catecholamines in the cytosol, the vesicular membrane-bound dopamine-beta-hydroxylase (*DBH*), which synthesizes norepinephrine, and the vesicular monoamine transporter (*VMAT2*), which is responsible for the transfer of catecholamines into the storage vesicles. Significantly higher *MAOA* mRNA levels (up to 15 mean-fold, Table S3) (*p* < 0.0001) were detected in the whole blood samples of the ICU/CoV-2 group (median = 0.348, IQR = 0.159–0.586) compared to the Healthy group (median = 0.020, IQR = 0,011–0.030), with a high discriminating accuracy (AUC = 0.992, 95% CI = 0.978–1.000) (Figure 7A). The cut-off value was calculated at 0.103, with a sensitivity of 93.33% and a specificity of 96.88%. In addition, a slight increase of *MAOA* expression (up to 3.6 mean-fold, Table S3) (*p* = 0.046) was detected in the Ward/CoV-2 patients (median = 0.066, IQR= 0.012–0.147) compared to the healthy subjects. However, our results on the Ward/CoV-2 cohort were characterized by a lower diagnostic accuracy (AUC = 0.698, 95% CI = 0.564–0.833), and an optimal cut-off value at 0.097, with a sensitivity and a specificity of 40% and 96.88%, respectively. Interestingly, *MAOB* expression was similarly elevated in both the ICU/CoV-2 (median = 0.060, IQR = 0.019–0.115) and the Ward/CoV-2 groups (median = 0.065, IQR = 0.026–0.119) (up to 10 mean-fold, *p* < 0.0001), compared to the healthy subjects (median = 0.005, IQR = 0.003–0.009) (Figure 7B). The ROC curve analysis showed a high diagnostic accuracy for *MAOB* expression in the ICU/CoV-2 (AUC = 0.922, 95% CI = 0.843–1.000) and the Ward/CoV-2 (AUC = 0.977, 95% CI = 0.951–1.000) cohorts. For the ICU/CoV-2 group, the optimal cut-off point was estimated at 0.021 (73.33% sensitivity and 97.06 specificity). The cut-off for the Ward/CoV-2 group was 0.021 (80% sensitivity and 97.06% specificity). The elevated expression of *MAOA* and *MAOB* in the whole blood samples is in agreement with the reduced serum dopamine levels in these patients. On the contrary, the expression of *DBH* and *VMAT2* in the SARS-CoV-2 infected cohorts did not exhibit any significant alteration in comparison to the healthy subjects (Figure 7C,D). Finally, the expression of these genes did not correlate either with sex or age (Figures S6D–G and S10).

### 3.6. Correlation between the Expression of DDC with dACE2 and MAOs in Whole Blood Samples of Hospitalized COVID-19 Patients

**Figure 8:**
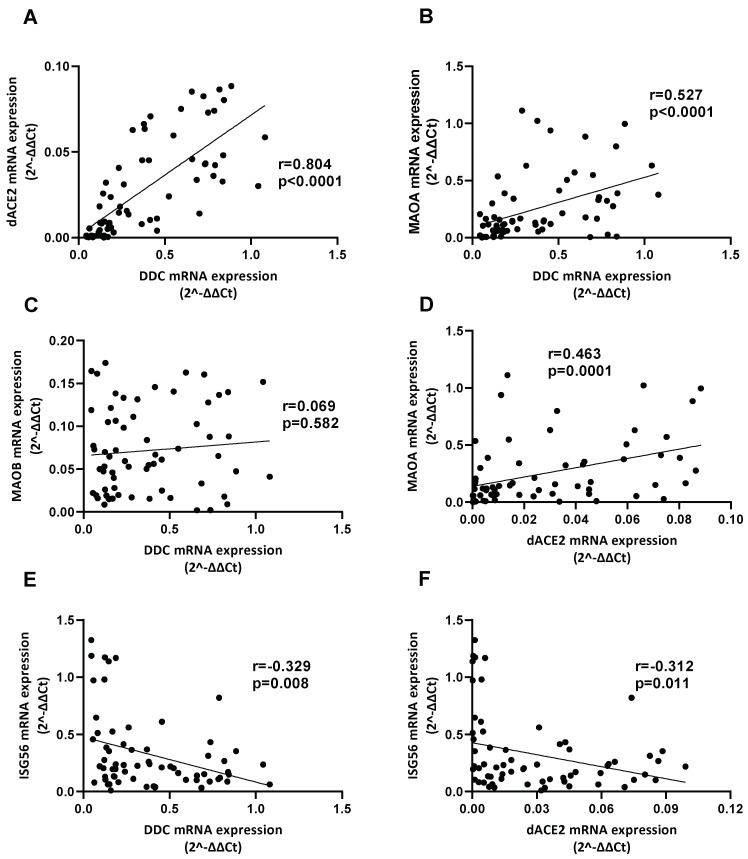
Correlation between the mRNA levels of (**A**) *DDC-dACE2*, (**B**) *DDC-MAOA*, (**C**) *DDC-MAOB*, (**D**) *dACE2-MAOA*, (**E**) *DDC-ISG56* and (**F**) *dACE2-ISG56* in whole blood samples of hospitalized COVID-19 patients. Data are presented as XY scatter plots and fitted linear regression lines. Pearson’s or Spearman’s correlation coefficient (r) and *p*-values (p) were calculated.

The two hospitalized SARS-CoV-2 patient cohorts (Ward/CoV-2 and ICU/CoV-2) were merged, and co-expression correlations for all studied genes were examined in their whole blood samples. A strong positive correlation between the expression of *DDC* and *dACE2* was detected in the SARS-CoV-2 infected patients and the healthy subjects (r = 0.804, *p* < 0.0001 and r = 0.689, *p* < 0.0001, respectively) (Figure 8A and Figure S11A), as determined by the Spearman’s correlation coefficient. This is in agreement with our previous data from the nasopharyngeal swab samples of COVID-19 patients with mild/no [19] or severe symptoms (Section 3.2). Moreover, a moderate positive correlation between the expression of *DDC* and *MAOA* (r = 0.527, *p* < 0.0001) was identified in the SARS-CoV-2 infected group, while there was no correlation in the Healthy group (Figure 8B and Figure S11B). No correlation was found between *DDC* and *MAOB* mRNA levels in either group (r = 0.069, *p* = 0.582) (Figure 8C and Figure S11C). Similarly, we observed that the expression of *dACE2* had a moderately strong positive correlation with *MAOA* (r = 0.4631, *p* = 0.0001) in the SARS-CoV-2 infected group (Figure 8D), and no correlation in the Healthy group (Figure S11D). No significant *dACE2* and *MAOB* co-expression was detected in neither the patients nor the healthy subjects (Figure S11E,F). Both *DDC* and *dACE2* negatively correlated with *ISG56* (r = −0.329, *p* = 0.0075 and r = −0.312, *p* = 0.0114, respectively) only in the COVID-19 cohort (Figure 8E,F and Figure S11G,H).

To exclude the possibility that our results in the whole blood expression of the aforementioned-genes in the hospitalized COVID-19 patients are affected by the presence of comorbidities, we grouped the SARS-CoV-2 infected subjects according to the presence of underlying diseases. Our results showed that the mRNA levels of the catecholamine pathway-related genes (*DDC*, *MAOA*, and *MAOB*) were not affected (Figure S12A–C) by the presence of comorbidities, while *dACE2* expression was negatively affected (*p* = 0.0083) (Figure S12D). Thus, the observed alterations in the whole blood expression of the catecholamine biosynthesis/metabolism genes in hospitalized COVID-19 patients are due to SARS-CoV-2 infection.

### 3.7. The effect of SARS-CoV-2 Infection on Catecholamine Pathway-Related Gene Expression in Cultured Epithelial Cells

**Figure 9:**
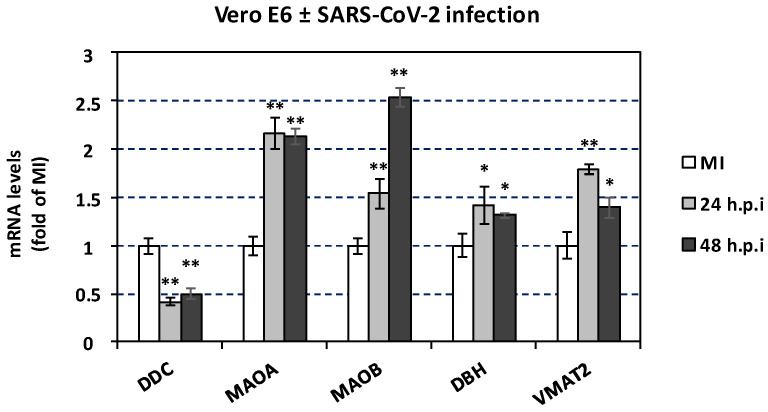
Effect of SARS-CoV-2 infection on *DDC*, *MAOA*, *MAOB*, *DBH* and *VMAT2* gene expression in infected cultured cells. Vero E6 cells were infected or mock-infected (MI) with virions of the SARS-CoV-2 isolate 30–287 of lineage B1. The cells were lysed at 24 and 48 h post-infection. The mRNA levels of *DDC*, *MAOA*, *MAOB*, *DBH* and *VMAT2* were quantified by RT-qPCR and the mRNA levels of the housekeeping gene YWHAZ was used for normalization. Bars represent mean values from three independent experiments in triplicates. Error bars indicate standard deviations. *p*-values were determined by the Student’s *t*-test. (MI: mock-infected cells) * *p* < 0.01 vs. MI, ** *p* < 0.0001 vs. MI.

**Figure 10:**
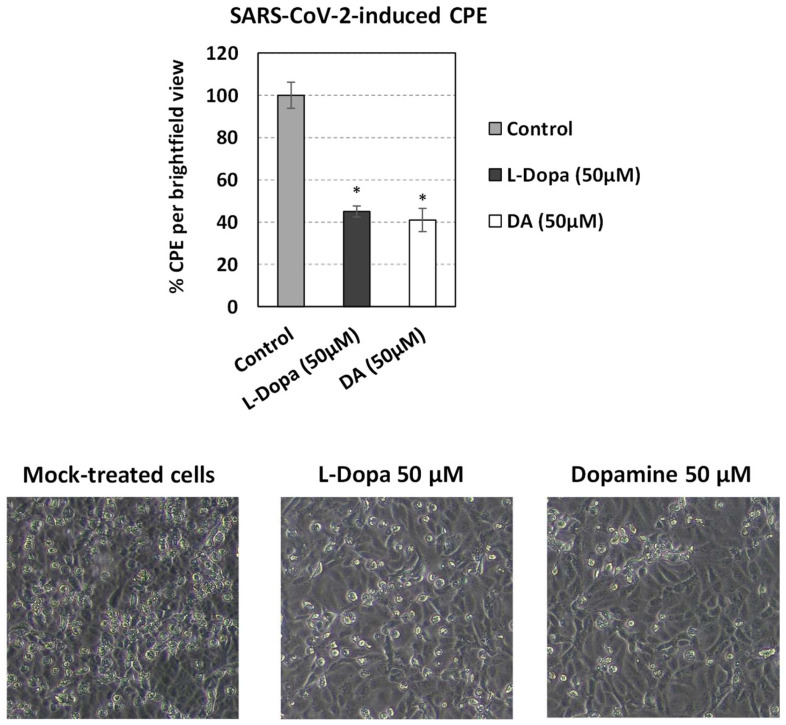
Effect of the DDC biosynthetic product (dopamine) and the DDC substrate (L-Dopa) on the SARS-CoV-2-induced cytopathic effect (CPE) in cell culture. Vero E6 cells were inoculated with SARS-CoV-2 (m.o.i. = 0.1) for 1 h and were subsequently treated, or not-treated (Control), with non-cytotoxic concentrations of L-Dopa or dopamine (DA). The virus-induced CPE was determined using an inverted microscope and representative images are shown in the lower panel. The observed CPE in the infected non-treated cells was set to 100%. Bars represent mean values from three independent experiments in triplicates. Error bars indicate standard deviations. *p*-values were determined by the Student’s *t*-test. * *p* < 0.0001 vs. Control.

We have previously shown that SARS-CoV-2 infection significantly reduces *DDC* expression in Vero E6 infected cells at 24 and 48 h post-infection (h.p.i.) [19]. This was also observed in A549 cells, as indicated by data derived from the RNA-sequencing Skyline database (http://rstats.immgen.org/Skyline_COVID-19/skyline.html (accessed on 28 August 2022)) [73]. To support our aforementioned in vivo results on the effect of SARS-CoV-2 infection on the expression of catecholamine-related genes with cell culture data, the expression of *MAOA*, *MAOB*, *DBH*, and *VMAT2* in Vero E6 cells infected for 24 and 48 h was studied. As shown in Figure 9, SARS-CoV-2 infection significantly increased the expression of *MAOA* (up to 2-fold) at both time points. A moderate increase in *MAOB* expression at 24 h post-infection, followed by a substantial increase (up to 2.5-fold) at 48 h post-infection was detected. Slightly enhanced mRNA levels of *DBH* were detected at both time points compared to the mock-infected control (Figure 9). Moreover, the expression of *VMAT2* was significantly enhanced especially at 24 h post-infection. To complement these results with data from human epithelial cells, we studied the impact of SARS-CoV-2 on the expression of catecholamine-related genes in A549 cells, by processing data from the Skyline database. Higher mRNA levels of *MAOA*, *MAOB* and *VMAT2* were observed upon infection in these cells also, while *DBH* and *COMT* expression was not significantly altered (Figure S13). To conclude, SARS-CoV-2 negatively altered the mRNA levels of *DDC*, whereas *MAOA* and *MAOB* positively, possibly to reduce the intracellular dopamine levels. In turn, non-cytotoxic concentrations of dopamine or L-Dopa (Figure S14) in SARS-CoV-2 infected Vero E6 cells decreased the virus-related cytopathic effect, by 55% and 59%, respectively (Figure 10). This suggests that there is a reverse relationship between dopamine levels and SARS-CoV-2 replication in vitro, possibly mediated through dopamine or L-Dopa signaling and/or elevation of the intracellular levels of dopamine. Thus, the effects on the expression of the catecholamine pathway-related genes observed upon infection of Vero E6 cells, which possibly result in lower dopamine levels, favor the propagation of SARS-CoV-2 virus. Therefore, these are possibly caused by the virus itself and not as a cell antiviral response.

### 3.8. Effect of SARS-CoV-2 Infection on the Expression of Hypoxia-Response Genes That Are Correlated with the Catecholamine Pathway, in the Blood of Hospitalized COVID-19 Patients and in Cultured Epithelial Cells

**Figure 11:**
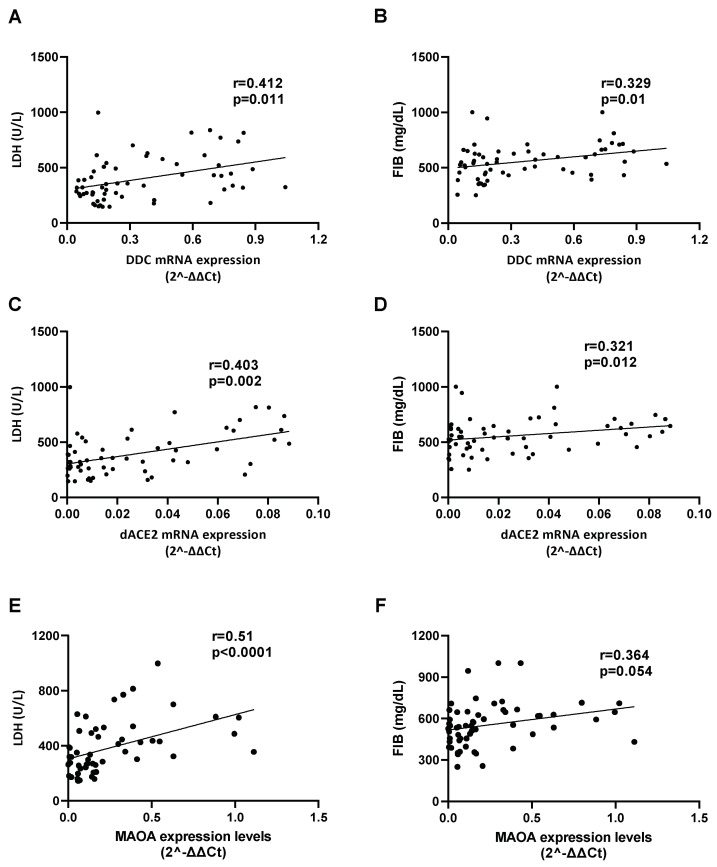
Correlation between the mRNA levels of (**A,B**) *DDC*, (**C,D**) *dACE2*, and (**E,F**) *MAOA* with the protein levels of LDH and FIB respectively, in whole blood samples of hospitalized COVID-19 patients. Data are presented as XY scatter plots and fitted linear regression lines. Pearson’s or Spearman’s correlation coefficient (r) and *p*-values (p) were calculated.

**Figure 12:**
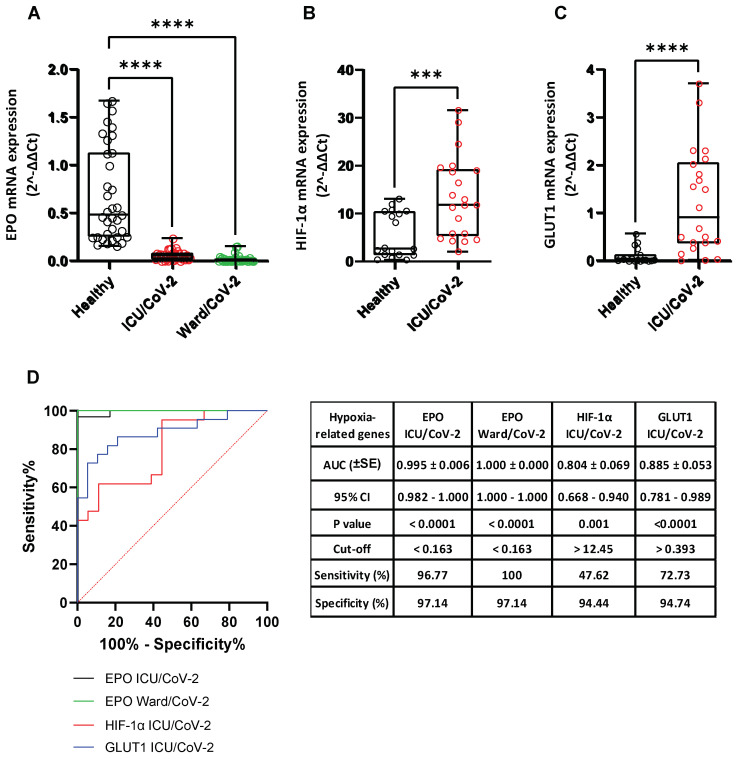
Box plot diagram of the mRNA expression of *EPO* (**A**), *HIF-1α* (**B**) and *GLUT1* (**C**) in whole blood samples of healthy subjects and hospitalized SARS-CoV-2 patients (ICU/CoV-2, Ward/CoV-2). *p*-values were determined by the non-parametric Kruskal–Wallis test, and multiple comparisons were conducted with Dunn’s post hoc analysis. *** *p* < 0.001, **** *p* < 0.0001 vs. Healthy. (**D**) ROC curve analysis for the aforementioned-genes expression. AUC, 95% CI, *p*-values, and cut-off points with their specificity and sensitivity were reported. The red dotted line denotes perfect chance (positive likelihood ratio = sensitivity/(1-specificity) = 1).

**Figure 13:**
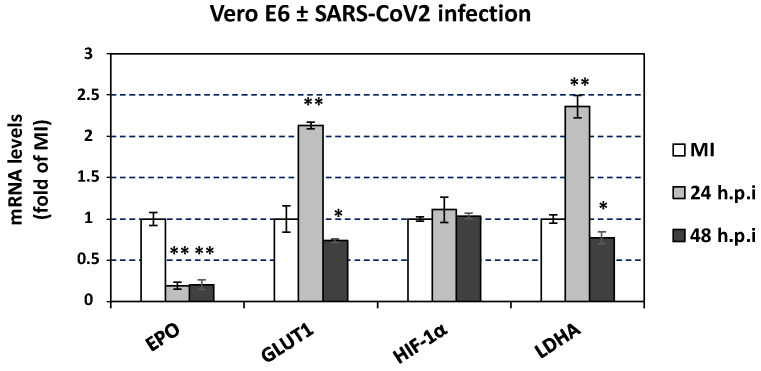
Effect of SARS-CoV-2 infection in *DDC*, *EPO*, *GLUT1*, *HIF-1α* and *LDHA* gene expression in infected cultured cells. Vero E6 cells were infected or mock-infected (MI) with virions of the SARS -CoV-2 isolate 30–287 of lineage B1. The cells were lysed at 24 and 48 h post-infection. The mRNA levels of *DDC*, *EPO*, *GLUT1*, *HIF-1α*, and *LDHA* were quantified by RT-qPCR and the mRNA levels of the housekeeping gene *YWHAZ* was used for normalization. Bars represent mean values from three independent experiments in triplicates. Error bars indicate standard deviations. *p*-values were determined by Student’s *t*-test. * *p* < 0.001, ** *p* < 0.0001 vs. MI.

Finally, we examined whether any of the laboratory parameters of the COVID-19 hospitalized patients, that were affected by SARS-CoV-2 infection, are related to the expression of the catecholamine pathway genes under study and *dACE2* in the blood. Interestingly, as depicted in Figure 11, the mRNA levels of *DDC*, *dACE2*, and *MAOA* showed a moderate correlation with the protein levels of lactate dehydrogenase (LDH) (r_DDC_ = 0.412, p_DDC_ = 0.0011; r_dACE2_ = 0.403, p_dACE2_ = 0.002; r_MAOA_ = 0.51, p_MAOA_ < 0.0001, respectively) and of fibrinogen (FIB) (r_DDC_ = 0.329, p_DDC_ = 0.01; r_dACE2_ = 0.321, p_dACE2_ = 0.012; r_MAOA_ = 0.364, p_MAOA_ = 0.005, respectively). Because both *LDH* and *FIB* are hypoxia-regulated genes, we also investigated the expression of the hypoxia marker genes *EPO*, *GLUT1,* and *HIF-1α* in whole blood samples of the two COVID-19 patient cohorts (Ward/CoV-2 and ICU/CoV-2) and compared them to the healthy subjects. The mRNA levels of *EPO* were significantly decreased in both ICU/CoV-2 (median = 0.056, IQR = 0.015–0.084) and Ward/CoV-2 (median = 0.010, IQR = 0.0016–0.022) groups, as compared to the healthy individuals (median = 0.480, IQR = 0.250–1.131) (Figure 12A); the highest reduction (up to 30 mean-fold, *p* < 0.0001) was measured in the Ward/CoV-2 group. The expression of *EPO* in the ICU/CoV-2 and Ward/CoV-2 cohorts was characterized by a high diagnostic accuracy, as determined by the ROC curve analysis (AUC = 0.995, 95% CI = 0.982–1.000, *p* < 0.0001 and AUC = 1.000, 95% CI = 1.000–1.000, *p* < 0.0001, respectively). The optimal cut-off value for both ICU/CoV-2 and Ward/CoV-2 was calculated at 0.163. For the ICU/CoV-2 patients the sensitivity was 96.77% and the specificity was 97.14%, while for the Ward/CoV-2 the values were 100% and 97.14%, respectively. In contrast, *HIF-1α* and *GLUT1* mRNA levels were excessively higher in the ICU/CoV-2 group (median_HIF-1α_ = 11.79, IQR = 5.301–19.28; median_GLUT1_ = 0.906, IQR = 0.359–2.054) than in the Healthy group (median_HIF-1α_ = 2.691, IQR = 1.359–10.50; median_GLUT1_ = 0.029, IQR = 0.017–0.132) (Figure 12B,C). More specifically, we observed an up to 2.5 mean-fold (*p* < 0.001) increase for *HIF-1α* and a 12 mean-fold (*p* < 0.0001) increase for *GLUT1* expression. Both *HIF-1α* (AUC = 0.804, 95% CI = 0.668–0.940, *p* < 0.0001) and *GLUT1* (AUC = 0.885, 95% CI = 0.781–0.989, *p* < 0.0001) were characterized by a high diagnostic accuracy, as evaluated by the ROC curve analysis. Furthermore, we examined the expression of *GLUT1*, *HIF-1α* and *LDHA* in our SARS-CoV-2 infected Vero E6 cells. While *EPO* was downregulated, *GLUT1* and *LDHA* expression was significantly induced upon infection especially at 24 h post infection. The mRNA levels of *HIF-1α* (Figure 13) were not affected. The observed upregulation of hypoxia target genes (*HIF-1α*, *GLUT1*) and the correlation of hypoxia-related proteins (LDH, FIB) with the decreased DDC and increased MAOA mRNA levels in the patient group, combined with the cell culture data, suggest that SARS-CoV-2 triggers hypoxia-like conditions to suppress dopamine levels, favoring its replication.

### 3.9. Meta-Analysis of the Direct Protein-Protein Interactions of the Catecholamine Biosynthesis/Metabolism and Hypoxia Pathways with Specific SARS-CoV-2 Proteins

**Figure 14:**
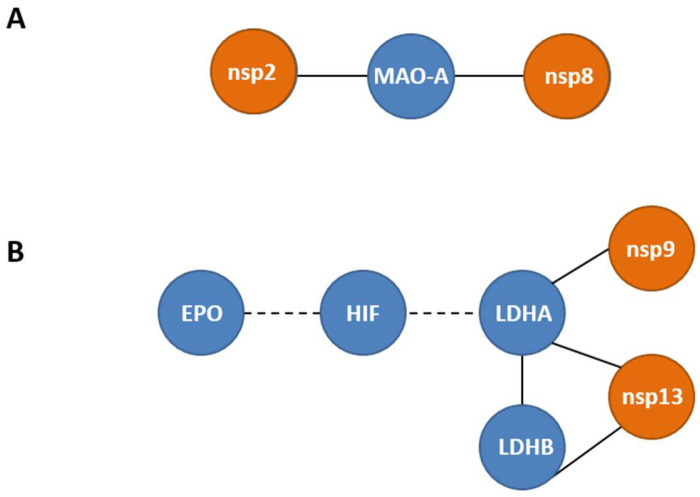
PPI sub-networks between (**A**) MAOA and SARS-CoV-2 non-structural proteins nsp2 and nsp8. (**Β**) PPI sub-networks between, LDHA and LDHB and the viral nsp9 and nsp13 proteins. Dashed lines depict the transcriptional regulation of *LDHA* and *EPO* genes by the HIF transcription factor.

Finally, by performing meta-analysis of published protein-protein interaction (PPIs) data, we further investigated the association of specific SARS-CoV-2 proteins with the main components of the catecholamine biosynthesis/metabolism and hypoxia pathways. Two main sub-networks of experimentally supported PPIs were identified for the proteins whose gene expression was described above, in both COVID-19 patients and infected cultured epithelial cells. The first involves direct PPIs of the SARS-CoV-2 non-structural proteins 2 and 8 (nsp2, nsp8) with the dopamine catabolizing enzyme MAOA (Figure 14A). In the second sub-network (Figure 14B), experimental PPIs were identified between the viral non-structural proteins 9 and 13 (nsp9, nsp13) and L-lactate dehydrogenase A (LDHA) and B chain (LDHB), respectively. Moreover, a bridging interconnection between three human host factors (Figure 14B), HIF that transcriptionally regulates both *EPO* and *LDHA* genes, and a three-member clique constructed by two host genes (*LDHA*, *LDHB*) and one viral (*nsp13*), were revealed.

## 4. Discussion

Our group has previously identified the impact of SARS-CoV-2 infection on the gene expression of the dopamine biosynthetic enzyme L-Dopa decarboxylase *(DDC)*, and of other genes associated with *DDC* and SARS-CoV-2 infection, such as the *ACE2*/*dACE2* locus, and the HIF-target gene *EPO*, in the nasopharyngeal swab samples of COVID-19 patients with mild or no symptoms. These genes were selected based on their role in the infection cycle of SARS-CoV-2 (*ACE2*) [13], as markers of interferon signaling (*ISG56*, *dACE2*) [17,18,74], or survival (*EPO*) [19]—especially under hypoxic conditions—and based on previous data showing the co-expression link between *DDC* and *ACE2* locus [19,28], and their regulation under hypoxic conditions [39,46,47,48,49,51]. The expression of these genes was found excessively enhanced in the nasopharyngeal tissue of COVID-19 patients, and interestingly, the mRNA levels of *DDC* and *dACE2* had a reverse correlation with the viral load. The *DDC* expression regulation appeared to be SARS-CoV-2 infection-specific, as no effect was observed in respective samples of flu-infected patients. On the other hand, based on our cell culture-derived results, SARS-CoV-2 infection downregulated the expression of the aforementioned-genes in Vero E6 cells. Consequently, our previous data from human samples and in vitro studies, suggest that the expression of these genes is affected by SARS-CoV-2-infection, and for the first time reveal that the virus influences the expression of *DDC*, the product of which catalyzes the production of dopamine, the first catecholamine of the respective pathway. The difference observed in gene expression regulation in the nasopharyngeal swab samples of SARS-CoV-2 infected subjects with mild/no symptoms, compared to the in vitro infection studies, could be attributed to the antiviral response of the host attempting to restrict the virus propagation. Moreover, a strong correlation between the expression of *DDC* and *dACE2* was identified in the patient group, reinforcing the possibility of the involvement of DDC in this antiviral response mechanism against the SARS-CoV-2 virus.

In the present study, we aimed to expand our previous findings, by analyzing the mRNA levels of the same genes in nasopharyngeal swab and whole blood samples of COVID-19 patients with severe symptoms of the disease.

Our results from the three swab cohorts (Mild CoV-2, Severe CoV-2, Negative) identified *DDC* expression as a potential marker of COVID-19 severity. In contrast to the *DDC* expression data obtained from the nasopharyngeal swabs of COVID-19 patients with mild/no symptoms [19], the severe cases of the disease exhibited lower *DDC* mRNA levels compared to the SARS-CoV-2-negative subjects. Additionally, in the blood of hospitalized COVID-19 patients (Ward/CoV-2 group and ICU/CoV-2 group), *DDC* mRNA levels were reduced, while no alteration was observed in the group of ICU non-COVID-19 patients (ICU/non-CoV-2), compared to the group of healthy subjects (Healthy). Notably, the presence of underlying comorbidities, with hypertension being the most frequent, did not seem to have an impact on the effect of SARS-CoV-2 infection on *DDC* expression in the hospitalized COVID-19 patients, as similar values were observed in patients with and without comorbidities. Moreover, *DDC* whole blood mRNA levels in both the ward and ICU groups of COVID-19 patients had a high diagnostic accuracy. The expression of *ACE2* in the nasopharyngeal swab samples of COVID-19 patients exhibited the same pattern of changes as *DDC*; its expression was induced in patients with mild symptoms, while reduced in patients with severe symptoms. Nonetheless, the mRNA levels of this gene in both groups were only slightly altered compared to the non-COVID-19 group (Negative). Additionally, we were unable to detect the mRNA of *ACE2* in the whole blood samples and thus, we could not examine whether its expression was affected by SARS-CoV-2 infection, or by the presence of underlying diseases. For the remaining genes, the expression of both *dACE2* and *EPO* was induced in the nasopharyngeal swabs of COVID-19 patients, failing, however, to distinguish patients with mild from the ones with severe symptoms. In the whole blood samples of hospitalized COVID-19 patients, *dACE2* and *EPO* mRNA levels were diminished, compared to healthy subjects. The expression of *dACE2* seemed to be influenced by underlying comorbidities. Finally, the expression of *ISG56* in the nasopharyngeal swab samples of COVID-19 patients exhibited a lower induction than that of *DDC* in patients with mild symptoms, whereas the group of patients with severe symptoms had equal *ISG56* mRNA levels with the non-COVID-19 group (Negative). The *ISG56* expression had a much lower diagnostic accuracy compared to *DDC* expression. On the other hand, in the whole blood samples, *ISG56* was significantly downregulated in both hospitalized COVID-19 patient cohorts, in accordance with previous reports on the downregulation of IFN-1 signaling by SARS-CoV-2 [75,76]. Therefore, although *dACE2* and *ISG56* are considered interferon-stimulated genes, in the nasopharyngeal tissue of the severe COVID-19 cases, their mRNA levels were differentially regulated upon infection. This is in agreement with our observations in SARS-CoV-2 infected Vero E6 cells at 24 h.p.i. [19], suggesting that *dACE2* expression may be also subjected to other regulation mechanisms in viral infection. Moreover, in the blood of hospitalized COVID-19 patients, the mRNA levels of *DDC* and of *dACE2* negatively correlated with *ISG56*. This finding could suggest that *DDC* and *dACE2* expression is associated with the excessive activation of the host immune response against SARS-CoV-2 observed in severe cases. More importantly, in the nasopharyngeal tissue, which is the most easily accessible and most commonly obtained sample from COVID-19 patients, the expression of *DDC* could serve as the most reliable marker of COVID-19 severity among the genes studied. Hence, it might be able to readily distinguish patients requiring hospitalization.

Viral infection has been shown, by our previous studies on HCV and DENV, to affect, in addition to *DDC*, the expression of other genes encoding enzymes of the catecholamine biosynthesis and metabolism, or catecholamine transporters [37,38]. Therefore, in the whole blood samples of hospitalized COVID-19 patients, we examined the gene expression of *MAOA* and *MAOB*, the products of which perform the oxidative deamination of biogenic amines, including dopamine, norepinephrine, and serotonin, *DBH*, which converts dopamine to norepinephrine, and of the catecholamine transporter-gene *VMAT2*. Moreover, the expression of these genes was analyzed in SARS-CoV-2-infected Vero E6 cells. Our results showed that SARS-CoV-2 infection did not affect the expression of *DBH* and *VMAT2*, but elevated, however, the mRNA levels of *MAOA* and *MAOB* in infected cells and in the whole blood samples of hospitalized COVID-19 patients. Interestingly, the MAOA enzyme has been shown to directly interact with the SARS-CoV-2 non-structural proteins nsp2 and nsp8 [41], which reinforces the association of the catecholamine degradation pathway with viral infection. Similar to the *DDC* mRNA expression, the presence of comorbidities did not affect the mRNA levels of *MAOA* and *MAOB* in the whole blood samples of hospitalized COVID-19 patients. Therefore, SARS-CoV-2 infection negatively affects the biosynthetic part and positively the catabolizing part of the dopamine biosynthesis/catabolism pathway both in vitro and in the blood tissue of infected patients with severe symptoms of the disease. At the cellular level, this SARS-CoV-2-mediated regulation of the catecholamine biosynthesis/metabolism pathway could diminish the levels of catecholamines in the cytosol. However, the mRNA levels of *MAOA* positively correlated with *DDC* in the blood samples of hospitalized COVID-19 patients, raising a question on the role of serum dopamine levels in these patients, as dopamine is a product of DDC and a substrate of MAOA enzymatic activities. Diminished dopamine levels were found in the hospitalized COVID-19 patients compared to the two control groups, the ICU non-COVID-19 and the Healthy ones. This is in agreement with the results obtained in the same tissue on the mRNA levels of *DDC* and *MAOA*/*-B*. Moreover, neither the underlying comorbidities nor mechanical ventilation seemed to influence dopamine levels. Therefore, the increased expression of *MAOA*/*-B* in the blood were not due to enhanced levels of dopamine, but possibly contribute to its reduction. Interestingly, the reduction in blood dopamine levels in COVID-19 patients was predicted first by Nataf et al. based on the effects of SARS-CoV-2 on the catecholamine biosynthesis/metabolism pathway in vitro in infected enterocytes [35].

In turn, exogenous administration of L-Dopa and dopamine in SARS-CoV-2-infected epithelial cells negatively influenced the virus-derived cytopathic effect. Dopamine treatment has been shown to negatively affect SARS-CoV-2 replication, by inducing Type-I interferons and ISG genes, while downregulating the gene expression of pro-inflammatory mediators [36]. Another explanation, supported by our previous data on Huh7 cells, could be a negative effect on the virus exerted by L-Dopa and dopamine uptake by the cells, which then convert L-Dopa to dopamine. Therefore, similarly to HCV and Dengue viruses [37,38], the enhancement of the dopamine intracellular levels could also be responsible for the reduced cytopathic effect observed in SARS-CoV-2 infection. These highlight that L-Dopa or dopamine treatment could be a potential antiviral therapeutic approach especially in cases where interferon response is impaired leading to increased viral titers of SARS-CoV-2 [77]. The dopamine concentrations used in the above in vitro studies are significantly higher than those detected in human blood; however, dopamine levels in many organs/tissues differ substantially from those of blood. Interestingly, in the rodent kidney the detected dopamine levels are comparable to those used in our study to treat the kidney-derived Vero E6 cells [78]. Moreover, we showed that the expression of *DDC* and *dACE2* maintain their strong positive correlation in both swab and blood samples, even though their mRNA levels were reversely regulated in the swab samples of COVID-19 severe cases. In the nasopharyngeal swab and blood samples, there are no indications that the gene expression results are attributed to an enrichment of inflammation related cell populations, as no significant differences were observed in the expression of the studied markers of the immune populations among the different groups of patients.

Blood samples from the hospitalized SARS-CoV-2-infected patients, before infection were not available. Therefore, we cannot exclude the possibility that these subjects might have lower dopamine and *DDC* expression levels compared to the general population, which could render them more susceptible to severe forms of COVID-19. In this case, dopamine levels could serve as a prognostic marker of disease severity. Moreover, dopamine intake to restore normal levels would probably have significant preventive and/or therapeutic value in these patients. Single nucleotide polymorphisms (SNPs) in the coding region of *DDC*, which have been reported to be associated with neurological disorders and response to treatment, may be related to reduced activity of the enzyme [79,80]. The frequency of two such polymorphisms in *DDC* (SNP ID: rs188088947 and SNP ID: rs11575542), which produce missense mutations, was compared between the group of COVID-19 patients with mild/no symptoms and the severe/critical. We did not detect any enrichment of these alleles in the group of COVID-19 patients with severe symptoms (data not shown). However, we cannot exclude the possibility of enrichment, in the group of COVID-19 patients with severe symptoms, of other *DDC* alleles that may affect for example the expression of the gene.

The levels of LDH and FIB proteins in the blood samples of the hospitalized COVID-19 patients were the only in-hospital routinely measured laboratory parameters that had a moderate positive correlation with the expression of *DDC*, *dACE2*, and *MAOA* genes in this tissue. Furthermore, SARS-CoV-2 severity has been previously linked with higher levels of LDH and FIB proteins [81,82,83,84,85,86,87]. Consistently, increased levels of these proteins were detected in the whole blood samples of the hospitalized groups of COVID-19 patients of the present study, compared to healthy subjects. The expression of the *LDH* gene was also enhanced in the SARS-CoV-2-infected Vero E6 cells at 24 h.p.i. Although LDH protein levels are usually indicative of tissue/cell damage, the expression of this gene is positively regulated by hypoxia, as the encoded product is a key enzyme of the anaerobic glycolysis pathway the activity of which is induced under low oxygen conditions. Interestingly, the mRNA levels of *HIF-1α* and *GLUT1* genes were also found significantly elevated in the whole blood samples of the ICU COVID-19 patients. Gene *HIF-1α* encodes the subunit of the HIF transcription factor that is stabilized by hypoxia, while *GLUT1* is one of the many genes that the HIF factor upregulates. Gene *GLUT1* was also induced in SARS-CoV-2-infected Vero E6 cells at 24 h.p.i. The increased levels of the LDH protein and the induction of the expression of *HIF-1α* and *GLUT1* in the blood samples of the hospitalized COVID-19 patients, are in agreement with several studies highlighting that severe clinical manifestations of COVID-19 are linked with induction of anaerobic glycolysis [88,89,90], and generation of hypoxia-like conditions [91,92]. All the above data, combined with our previous results from Vero E6 and A549 epithelial cells [19], suggest that hypoxia signaling constitutes a possible mechanism of co-regulation of the dopamine biosynthesis/catabolism genes (DDC, MAOA) and dACE2 during SARS-CoV-2 infection. Additionally, a weak positive correlation between the expression of the aforementioned-genes and fibrinogen (FIB) protein levels was detected in the same patient samples. Fibrinogen is known for its chemotactic activities and its role in homeostasis and antimicrobial host defense [93]. The SARS-CoV-2 infection has been reported to elevate fibrinogen levels in more severe cases of the disease [94], which in turn activates HIF-1α and thus induces anaerobic glycolysis [95]. This could suggest that our previously reported data from the in vitro studies, showing that the virus triggers hypoxia-like conditions and manipulates the glycolytic flux towards anaerobic glycolysis to favor its replication, may also reflect the in vivo situation. Additionally, the mRNA levels of *EPO* were significantly reduced in the SARS-CoV-2-infected Vero E6 cells and in the A549 cells cultured under low oxygen tension, even though the expression of *EPO* is known to be strongly induced by hypoxia. Both treatments resulted in extensive cell death, therefore it is possible that the expression of *EPO* is positively regulated by hypoxia, only when cell death mechanisms have not been activated. The *EPO* expression remained elevated in the nasopharyngeal swabs of hospitalized COVID-19 patients, yet it was severely downregulated in the whole blood samples of the respective group, compared to the healthy subjects. The latter is in agreement with previous reports [8,55,56]. In the nasopharyngeal tissue, the expression of *EPO* seems to be associated with the activation of cellular survival mechanisms [96,97]. On the other hand, in the whole blood samples, the expression pattern of *EPO* could either reflect the massive death of many cell populations observed in severe cases of SARS-CoV-2 infection [98], or it could be related to a possible virus-produced dysfunction in hypoxia signaling previously described as a putative mechanism of the ‘’hypoxia-paradox’’. According to the latter, in severe COVID-19 cases, reduced levels of the EPO protein are observed, despite the presence of low levels of hemoglobin in these patients [55,99]. Interestingly, recombinant human EPO was used successfully as a treatment in more severe cases of COVID-19 disease [100,101,102]. The observed elevated levels of LDH protein and increased expression of *HIF-1α* and *GLUT1* in the ICU COVID-19 patients likely correlate with an enhanced glucose utilization that could be evidence of the hypoxic conditions caused by the virus infection. Moreover, the interaction of LDHA and LDHB with the SARS-CoV-2 nsp9 and nsp13 proteins has been reported in previous studies. The enzymes LDHA and LDHB have been reported to be implicated in glycolysis and in TCA/oxidative phosphorylation, respectively [103]. These data may suggest that the virus regulates the cell energy-production pathways to favor its propagation. Meanwhile, EPO, which is also transcriptionally regulated by hypoxia (HIF), has not been shown to directly interact with viral proteins.

The present study has some limitations. Although the rapid breakdown of dopamine in most aqueous solutions is a disadvantage of ELISA, this method has been used elsewhere for dopamine measurement in biological fluids such as serum and urine [104,105,106] and seems to have similar results to HPLC [64]. Moreover, accumulated cytosolic-located dopamine, because of its administration in high concentrations, can undergo auto-oxidation, producing toxic quinones that can also generate ROS [38,107]. Consequently, we cannot exclude the implication of ROS on the observed dopamine-mediated reduction in virus-derived CPE. Interestingly, DENV, another virus that causes cell lysis, even though is favored by ROS production [108], is negatively affected by exogenously applied dopamine [38]. Finally, another limitation of this study is the relatively small numbers of patients in a few groups, and that age and sex were not well distributed between some cohorts.

## 5. Conclusions

This study suggests that *DDC* expression levels in the nasopharyngeal tissue could serve as the most reliable marker, amongst the genes studied, of SARS-CoV-2 disease severity. Hence, *DDC* expression levels could be used as a prognostic tool to avoid the occurrence of critical manifestations. Additionally, our study reveals a generalized regulation of the catecholamine biosynthesis and catabolism pathway upon SARS-CoV-2 infection in the whole blood samples of hospitalized COVID-19 patients and infected cultured epithelial cells, providing novel knowledge on the viral pathogenesis. Furthermore, in the current study, we provide evidence that the ISG *dACE2*, which is co-expressed with *DDC* and *MAOA*, is possibly regulated by other factors, apart from interferon response, upon SARS-CoV-2 infection. Moreover, we demonstrated that the exogenous administration of non-cytotoxic concentrations of dopamine and L-Dopa in SARS-CoV-2-infected Vero E6 cells led to significantly lower levels of virus-derived cytopathic effect. Finally, we confirmed that SARS-CoV-2 infection induces hypoxia-like conditions in the cell, affecting the expression of catecholamine biosynthesis and catabolism genes, a condition that favors SARS-CoV-2 replication. Considering the above, further investigation is needed to address the impact of the products and precursors of the catecholamine biosynthesis pathway in the viral replication to possibly use them as a therapeutic approach.

## Figures and Tables

**Table 1:** Priming oligonucleotides used in the RT-qPCR analysis.

Gene.	Orientation	Sequence (5′-3′)	Reference
*DDC*	Forward	AGAGGGAAGGAGATGGTGGATTA	[33]
	Reverse	GGGGCTGTGCCAGTGCGT	
*DDC*	Forward	ACGCACTGGCACAGCCCC	
	Reverse	CACTCCTCCCCCTTCTCC	
*EPO*	Forward	GCCCCACCACGCCTCATCTGT	[59]
	Reverse	CTTCCAGGCATAGAAATTAAC	
*ISG56*	Forward	GGACAGGAAGCTGAAGGAG	[61]
	Reverse	AGTGGGTGTTTCCTGCAA	
*ACE2*	Forward	CGAAGCCGAAGACCTGTTCTA	[60]
	Reverse	GGGCAAGTGTGGACTGTTCC	
*dACE2*	Forward	GTGAGAGCCTTAGGTTGGATTC	[18]
	Reverse	TAAGGATCCTCCCTCCTTTGT	
*MAOA*	Forward	GGGCTGCTACACGGCCTACT	
	Reverse	GACCTCCCTAGCTGCTCGTTCT	
*MAOB*	Forward	GGAGCCAGTGCATTATGAAGA	
	Reverse	GCCTGCAAAGTAAATCCTGTC	
*VMAT2*	Forward	CGGATGTGGCATTTTGTATGG	
	Reverse	TTCTTCTTTGGCAGGTGGACTTC	
*DBH*	Forward	GCCATCCATTTCCAGCTCCT	
	Reverse	TCCAGGCGTCCGCAAAATAG	
*HIF-1α*	Forward	CATAAAGTCTCAACATGGAAGGT	
	Reverse	ATTTGATGGGTGAGGAATGGGTT	
*GLUT1*	Forward	CAGTTTGGCTACAACACTGGAGT	
	Reverse	ATAGCGGTGGACCCATGTCT	
*LDHA*	Forward	TTCACCCATTAAGCTGTCATGG	
	Reverse	GACACCAGCAACATTCATTCCA	
*EPCAM*	Forward	CGCAGCTCAGGAAGAATGTG	
	Reverse	TGAAGTACACTGGCATTGACG	
*CD45*	Forward	AAAAGTGCTCCTCCAAGCCA	
	Reverse	TGGGAGGCCTACACTTGACA	
*CD74*	Forward	CCGGCTGGACAAACTGACA	
	Reverse	GGTGCATCACATGGTCCTCTG	
*LYN*	Forward	TTCTGGTCTCCGAGTCACTCA	
	Reverse	GCCGTCCACTTAATAGGGAACT	
*YWHAZ*	Forward	GCTGGTGATGACAAGAAAGG	
	Reverse	GGATGTGTTGGTTGCATTTCCT	

**Table 2:** Demographic data of subjects whose nasopharyngeal swab samples were used.

	SARS-CoV-2			
Characteristics	Negative	Mild CoV-2	Severe CoV-2	*p* Value
**Total Number**	38	37	21	
**Median age in years (IQR)**	47 (34–68)	38 (31–52)	64 (61–75)	0.001 ^a^
**Sex**				
**Male**	14 (36.8%)	14 (37.8%)	17 (81%)	0.0065 ^b^
**Female**	23 (60.6%)	14 (37.8%)	4 (19%)	
**NR**	1 (2.6%)	9 (24.4%)	0	

IQR: inter-quartile range; ^a^: Kruskal–Wallis test; ^b^: Chi- square test; ΝR: Not recorded.

**Table 3:** Demographic data and in-hospital measured laboratory parameters of subjects whose blood samples were used.

	SARS-CoV-2			
Characteristics	Healthy	Ward/CoV-2	ICU/CoV-2	*p* Value
Total number	*n* = 35	*n* = 35	*n* = 32	
Median age in years (IQR)	49 (42–55)	59 (38–70)	62 (56–70)	*p* < 0.0001 ^a^
Sex				
Male	20 (57.2%)	25 (71.4%)	25 (78.1%)	0.2231 ^b^
Female	15 (42.8%)	10 (28.6%)	7 (21.9%)	
Comorbidities				
Hypertension (%)	-	5.71 (25.71 *)	9.38 (43.75 *)	
Diabetes (%)	-	2.86 (5.71 *)	0 (9.38 *)	
Other Comorbidities (%)	-	20	9.38	
>1 comorbidity (%)	-	31.43	43.75	
None (%)	100	40	37.5	
Clinical Measurements Median (IQR)				
Creatinine (mg/dL)	0.700–1.300	0.900 (0.700–1.300)	1.000 (0.800–1.200)	
CRP (mg/L)	8–10	5.300 (1.475–31.250)	14.400 (6.750–26.250)	
Urea (mg/dL)	6–24	25.500 (21.250–33.750)	31 (24–47.500)	
White blood cell count (per µL)	4500–11,000	5185 (4265–6565)	10,300 (5250–11,170) *	0.008 ^c^
Neutrophils (%)	70	67.500 (53.130–81.880)	83 (78–88) *	<0.0001 ^c^
Lymphocytes (%)	20–40	28.400 (16.500–37.450)	12.600 (8–18.500) *	0.0002 ^c^
Platelets (per µL)	150,000–450,000	184,500 (147,250–246,000)	206,000 (139,500–255,500)	
D-dimer (pg/mL)	<0.5	0.915 (0.505–8.750)	0.540 (0.210–1.590)	
AST (IU/L)	8–33	29.500 (20.500–50.500)	46 (36.500–61)	0.0029 ^c^
ALT (IU/L)	4–36	27 (18.250–41.500)	38 (26.500–49.500)	0.0179 ^c^
LDH (U/L)	105–333	266.500 (199–322.500)	489 (355.500–682.300)	<0.0001 ^c^
Fibrinogen (mg/dL)	200–400	480 (383.200–548)	627 (550.500–709)	<0.0001 ^c^

IQR: inter-quartile ranges; ^a^: Kruskal–Wallis test; ^b^: Chi-square test; ^c^: Mann–Whitney U test; * the percentages were calculated including patients with >1 comorbidity for each category; CRP: C-reactive protein; AST: aspartate aminotransferase; ALT: alanine transaminase; LDH: lactate dehydrogenase.

**Table 4:** Demographic data and in-hospital measured laboratory parameters of subjects whose sera were used.

	SARS-CoV-2					
Characteristics	Healthy	Ward/CoV-2	ICU/CoV-2	ICU/non-CoV-2	ICU/CoV-2 + Dexa	*p* Value
Total number, N	33	28	33	27	29	
Dopamine levels						
Median (IQR) (pg/mL)	197.8 (138.2–316.2)	62.22 (43.44–182.4)	76.43 (63.76–107.4)	146.3 (107.9–202.4)	93.06 (71.3–127.6)	<0.0001 ^a^
Median age in years (IQR)	57 (52–60)	55 (42–75)	62 (54–71)	67 (50–75)	61 (55–72)	0.041 ^b^
Sex						
Male	22	21	27	17	21	0.514 ^c^
Female	11	7	6	10	8	
Comorbidities						
Hypertension (%)		0 (17.86 *)	12.12 (45.45 *)	7.40 (14.81 *)	3.45 (37.93 *)	
Diabetes (%)	-	7.14 (10.71 *)	3.03 (18.18 *)	-	0 (10.34 *)	
Lipids (%)	-	0 (7.14 *)	6.06 (15.15 *)	0 (14.81 *)	3.45 (13.79 *)	
Other comorbidities (%)	-	10.71	6.06	7.41	10.34	
>1 comorbidity (%)	-	39.29	45.45	25.93	65.52	
None (%)	100%	42.86	27.28	59.26	17.24	

^a^: *p* value was calculated with the Kruskal–Wallis test; ^b^: *p* value was calculated with ordinary One Way ANOVA; ^c^ *p* value was determined with Chi squared test; * The percentages were calculated including patients with >1 comorbidity for each category; CRP: C-reactive protein; AST: aspartate aminotransferase; ALT: alanine transaminase; LDH: lactate dehydrogenase.

## Data Availability

All relevant data are within the manuscript and its Supporting information files.

## Supplementary Materials

The following supporting information can be downloaded at https://www.mdpi.com/article/10.3390/cells12010012/s1. **Table S1**: Mean values of the studied genes’ expression in nasopharyngeal tissue of SARS-CoV-2 positive or negative subjects. **Table S2:** Demographic data of individuals whose blood samples were used. **Table S3:** Mean values of the studied genes expression in whole blood samples of SARS-CoV-2 positive or negative subjects. **Figure S1**: Comparison of *DDC* (A), *ACE2* (B), *dACE2* (C), *ISG56* (D), or *EPO* (E) expression in the nasopharyngeal swab samples of COVID-19 patients between men and women. **Figure S2**: Correlation among the expression of *DDC* (A), *ACE2* (B), *dACE2* (C), *ISG56* (D), or *EPO* (E) with patients’ age (continuous parameter) in nasopharyngeal swab samples of COVID-19 patients with severe symptoms. **Figure S3**: Correlation among the expression of *ISG56* (A), *ACE2* (B), or *EPO* (C), with that of *dACE2* in the nasopharyngeal swab samples of COVID-19 patients with severe symptoms. **Figure S4**: Correlation among the expression of *DDC* (A), *ACE2* (B), *dACE2* (C), *ISG56* (D), or *EPO* (E) with SARS-CoV-2 viral titers in the nasopharyngeal tissue of severe COVID-19 cases. **Figure S5**: Comparison of SARS-CoV-2 viral load levels between COVID-19 patients with mild or no symptoms with more severe cases. **Figure S6**: Comparison of *DDC* (A), *dACE2* (B), *ISG56* (C), *MAOA* (D), *MAOB* (E), *DBH* (F), or *VMAT2* (G) expression between men and women, in whole samples of COVID-19 patients with severe symptoms. **Figure S7**: XY scatter plot with fitted linear regression lines of *DDC* (A), *dACE2* (B), or *ISG56* (C) expression versus age (continuous parameter) in whole blood samples of COVID-19 patients with severe symptoms (Left panels) or healthy subjects (Right panels). **Figure S8**: Box plot analysis comparing the mRNA expression of *CD45* (A), *CD74* (B) and *LYN* (C) in whole blood samples among Healthy individuals and COVID-19 hospitalized patients (ICU/CoV-2, Ward/CoV-2 groups). **Figure S9:** Serum dopamine levels of SARS-CoV-2 infected subjects in the presence of comorbidities. (A) Absolute dopamine levels in the serum of ICU COVID-19 (receiving DEXA or not) or ICU non-COVID-19 patients, with (Yes) or without (No) comorbidities. **Figure S10:** XY scatter plot with fitted linear regression lines of *MAOA* (A), *MAOB* (B), *DBH* (C), or *VMAT2* (D) expression versus age (continuous parameter) in whole samples of COVID-19 patients with severe symptoms (Left panels) or healthy subjects (Right panels). **Figure S11**: Correlation of the expression of all studied genes in the blood of COVID-19 severe cases and healthy individuals. **Figure S12**: Box plot analysis of the mRNA expression of *DDC* (A), *MAOA* (B), *MAOB* (C) and *dACE2* (D) in whole blood samples among the hospitalized COVID-19 patients, in the presence (Yes) or absence (No) of comorbidities. **Figure S13**: SARS-CoV-2 infection of A549 cells upregulated the expression of MAOA, MAOB, and VMAT2 24 h post-infection. **Figure S14**: Effect of DDC substrate (L-Dopa, A) and product (dopamine, B) on intracellular ATP levels.

## References

[1] World Health Organization (2022). Coronavirus Disease (COVID-19). https://www.who.int/emergencies/diseases/novel-coronavirus-2019/question-and-answers-hub/q-a-detail/coronavirus-disease-covid-19.

[2] Wu D., Wu T., Liu Q., Yang Z. (2020). The SARS-CoV-2 outbreak: What we know. Int. J. Infect. Dis..

[3] Huang C., Wang Y., Li X., Ren L., Zhao J., Hu Y., Zhang L., Fan G., Xu J., Gu X. (2020). Clinical features of patients infected with 2019 novel coronavirus in Wuhan, China. Lancet.

[4] Rahmani B., Ghashghayi E., Zendehdel M., Baghbanzadeh A., Khodadadi M. (2022). Molecular mechanisms highlighting the potential role of COVID-19 in the development of neurodegenerative diseases. Physiol. Int..

[5] (2022). Clinical Spectrum of SARS-CoV-2 Infection. https://www.covid19treatmentguidelines.nih.gov/overview/clinical-spectrum/.

[6] Teuwen L.A., Geldhof V., Pasut A., Carmeliet P. (2020). Author Correction: COVID-19: The vasculature unleashed. Nat. Rev. Immunol..

[7] Laforge M., Elbim C., Frere C., Hemadi M., Massaad C., Nuss P., Benoliel J.J., Becker C. (2020). Author Correction: Tissue damage from neutrophil-induced oxidative stress in COVID-19. Nat. Rev. Immunol..

[8] Jahani M., Dokaneheifard S., Mansouri K. (2020). Hypoxia: A key feature of COVID-19 launching activation of HIF-1 and cytokine storm. J. Inflamm..

[9] Del Valle D.M., Kim-Schulze S., Huang H.H., Beckmann N.D., Nirenberg S., Wang B., Lavin Y., Swartz T.H., Madduri D., Stock A. (2020). An inflammatory cytokine signature predicts COVID-19 severity and survival. Nat. Med..

[10] Hu B., Huang S., Yin L. (2021). The cytokine storm and COVID-19. J. Med. Virol..

[11] Diamond M.S., Kanneganti T.D. (2022). Innate immunity: The first line of defense against SARS-CoV-2. Nat. Immunol..

[12] Shakir S. (2020). Cytokine Storms: A Major Killer in Patients with Severe COVID-19 Infection. https://www.europeanpharmaceuticalreview.com/article/118067/cytokine-storms-a-major-killer-in-patients-with-severe-covid-19-infection.

[13] Hoffmann M., Kleine-Weber H., Krüger N., Müller M., Drosten C., Pöhlmann S. (2020). The novel coronavirus 2019 (2019-nCoV) uses the SARS-coronavirus receptor ACE2 and the cellular protease TMPRSS2 for entry into target cells. BioRxiv.

[14] Rodrigues R., Costa de Oliveira S. (2021). The Impact of Angiotensin-Converting Enzyme 2 (ACE2) Expression Levels in Patients with Comorbidities on COVID-19 Severity: A Comprehensive Review. Microorganisms.

[15] Choi J.H., Choi S.H., Yun K.W. (2022). Risk Factors for Severe COVID-19 in Children: A Systematic Review and Meta-Analysis. J. Korean Med. Sci..

[16] Gao Y.D., Ding M., Dong X., Zhang J.J., Azkur A.K., Azkur D., Gan H., Sun Y.L., Fu W., Li W. (2021). Risk factors for severe and critically ill COVID-19 patients: A review. Allergy.

[17] Onabajo O.O., Banday A.R., Stanifer M.L., Yan W., Obajemu A., Santer D.M., Florez-Vargas O., Piontkivska H., Vargas J.M., Ring T.J. (2020). Interferons and viruses induce a novel truncated ACE2 isoform and not the full-length SARS-CoV-2 receptor. Nat. Genet..

[18] Blume C., Jackson C.L., Spalluto C.M., Legebeke J., Nazlamova L., Conforti F., Perotin J.M., Frank M., Butler J., Crispin M. (2021). A novel ACE2 isoform is expressed in human respiratory epithelia and is upregulated in response to interferons and RNA respiratory virus infection. Nat. Genet..

[19] Mpekoulis G., Frakolaki E., Taka S., Ioannidis A., Vassiliou A.G., Kalliampakou K.I., Patas K., Karakasiliotis I., Aidinis V., Chatzipanagiotou S. (2021). Alteration of L-Dopa decarboxylase expression in SARS-CoV-2 infection and its association with the interferon-inducible ACE2 isoform. PLoS ONE.

[20] Zhou Y., Wang M., Li Y., Wang P., Zhao P., Yang Z., Wang S., Zhang L., Li Z., Jia K. (2021). SARS-CoV-2 Spike protein enhances ACE2 expression via facilitating Interferon effects in bronchial epithelium. Immunol. Lett..

[21] Chua R.L., Lukassen S., Trump S., Hennig B.P., Wendisch D., Pott F., Debnath O., Thurmann L., Kurth F., Volker M.T. (2020). COVID-19 severity correlates with airway epithelium-immune cell interactions identified by single-cell analysis. Nat. Biotechnol..

[22] Williams T.L., Strachan G., Macrae R.G.C., Kuc R.E., Nyimanu D., Paterson A.L., Sinha S., Maguire J.J., Davenport A.P. (2021). Differential expression in humans of the viral entry receptor ACE2 compared with the short deltaACE2 isoform lacking SARS-CoV-2 binding sites. Sci. Rep..

[23] Liou T.G., Adler F.R., Cahill B.C., Cox D.R., Cox J.E., Grant G.J., Hanson K.E., Hartsell S.C., Hatton N.D., Helms M.N. (2021). SARS-CoV-2 innate effector associations and viral load in early nasopharyngeal infection. Physiol. Rep..

[24] Busnadiego I., Fernbach S., Pohl M.O., Karakus U., Huber M., Trkola A., Stertz S., Hale B.G. (2020). Antiviral Activity of Type I, II, and III Interferons Counterbalances ACE2 Inducibility and Restricts SARS-CoV-2. mBio.

[25] Patel S.K., Juno J.A., Lee W.S., Wragg K.M., Hogarth P.M., Kent S.J., Burrell L.M. (2021). Plasma ACE2 activity is persistently elevated following SARS-CoV-2 infection: Implications for COVID-19 pathogenesis and consequences. Eur. Respir. J..

[26] Reindl-Schwaighofer R., Hodlmoser S., Eskandary F., Poglitsch M., Bonderman D., Strassl R., Aberle J.H., Oberbauer R., Zoufaly A., Hecking M. (2021). ACE2 Elevation in Severe COVID-19. Am. J. Respir. Crit. Care Med..

[27] Vassiliou A.G., Zacharis A., Keskinidou C., Jahaj E., Pratikaki M., Gallos P., Dimopoulou I., Kotanidou A., Orfanos S.E. (2021). Soluble Angiotensin Converting Enzyme 2 (ACE2) Is Upregulated and Soluble Endothelial Nitric Oxide Synthase (eNOS) Is Downregulated in COVID-19-induced Acute Respiratory Distress Syndrome (ARDS). Pharmaceuticals.

[28] Nataf S. (2020). An alteration of the dopamine synthetic pathway is possibly involved in the pathophysiology of COVID-19. J. Med. Virol..

[29] Dimitra Florou A.S., Vassilacopoulou D., Fragoulis E.G. (2011). DDC (dopa decarboxylase (aromatic L-amino acid decarboxylase)). Atlas Genet. Cytogenet. Oncol. Haematol..

[30] Arreola R., Alvarez-Herrera S., Perez-Sanchez G., Becerril-Villanueva E., Cruz-Fuentes C., Flores-Gutierrez E.O., Garces-Alvarez M.E., de la Cruz-Aguilera D.L., Medina-Rivero E., Hurtado-Alvarado G. (2016). Immunomodulatory Effects Mediated by Dopamine. J. Immunol. Res..

[31] Roumier A., Béchade C., Maroteaux L. (2019). Maroteaux, Serotonin and the Immune System. Paul M Pilowsky. Serotonin. The Mediator That Spans Evolution.

[32] Blows W.T. (2000). Neurotransmitters of the brain: Serotonin, noradrenaline (norepinephrine), and dopamine. J. Neurosci. Nurs..

[33] Vassiliou A.G., Siaterli M.Z., Frakolaki E., Gkogkosi P., Paspaltsis I., Sklaviadis T., Vassilacopoulou D., Vassilaki N. (2019). L-Dopa decarboxylase interaction with the major signaling regulator RhoIota3Kappa in tissues and cells of neural and peripheral origin. Biochimie.

[34] Chalatsa I., Arvanitis N., Arvanitis D., Tsakou A.C., Kalantzis E.D., Vassiliou A.G., Sideris D.C., Frakolaki E., Vassilaki N., Vassilacopoulou D. (2020). Human L-Dopa decarboxylase interaction with annexin V and expression during apoptosis. Biochimie.

[35] Nataf S., Pays L. (2021). Molecular Insights into SARS-CoV2-Induced Alterations of the Gut/Brain Axis. Int. J. Mol. Sci..

[36] Limanaqi F., Zecchini S., Dino B., Strizzi S., Cappelletti G., Utyro O., Vanetti C., Garziano M., Saulle I., Clerici M. (2022). Dopamine Reduces SARS-CoV-2 Replication In Vitro through Downregulation of D2 Receptors and Upregulation of Type-I Interferons. Cells.

[37] Mpekoulis G., Tsopela V., Panos G., Siozos V., Kalliampakou K.I., Frakolaki E., Sideris C.D., Vassiliou A.G., Sideris D.C., Vassilacopoulou D. (2021). Association of Hepatitis C Virus Replication with the Catecholamine Biosynthetic Pathway. Viruses.

[38] Mpekoulis G., Tsopela V., Chalari A., Kalliampakou K.I., Panos G., Frakolaki E., Milona R.S., Sideris D.C., Vassilacopoulou D., Vassilaki N. (2022). Dengue Virus Replication Is Associated with Catecholamine Biosynthesis and Metabolism in Hepatocytes. Viruses.

[39] Frakolaki E., Kalliampakou K.I., Kaimou P., Moraiti M., Kolaitis N., Boleti H., Koskinas J., Vassilacopoulou D., Vassilaki N. (2019). Emerging Role of l-Dopa Decarboxylase in Flaviviridae Virus Infections. Cells.

[40] Laurent E.M.N., Sofianatos Y., Komarova A., Gimeno J.-P., Tehrani P.S., Kim D.-K., Abdouni H., Duhamel M., Cassonnet P., Knapp J.J. (2020). Global BioID-based SARS-CoV-2 proteins proximal interactome unveils novel ties between viral polypeptides and host factors involved in multiple COVID19-associated mechanisms. BioRxiv.

[41] Stukalov A., Girault V., Grass V., Karayel O., Bergant V., Urban C., Haas D.A., Huang Y., Oubraham L., Wang A. (2021). Multilevel proteomics reveals host perturbations by SARS-CoV-2 and SARS-CoV. Nature.

[42] Perrin-Cocon L., Diaz O., Jacquemin C., Barthel V., Ogire E., Ramiere C., Andre P., Lotteau V., Vidalain P.O. (2020). The current landscape of coronavirus-host protein-protein interactions. J. Transl. Med..

[43] Liu X., Huuskonen S., Laitinen T., Redchuk T., Bogacheva M., Salokas K., Pohner I., Ohman T., Tonduru A.K., Hassinen A. (2021). SARS-CoV-2-host proteome interactions for antiviral drug discovery. Mol. Syst. Biol..

[44] Gordon D.E., Jang G.M., Bouhaddou M., Xu J., Obernier K., White K.M., O’Meara M.J., Rezelj V.V., Guo J.Z., Swaney D.L. (2020). A SARS-CoV-2 protein interaction map reveals targets for drug repurposing. Nature.

[45] Hoffmann H.H., Schneider W.M., Sanchez-Rivera F.J., Luna J.M., Ashbrook A.W., Soto-Feliciano Y.M., Leal A.A., le Pen J., Ricardo-Lax I., Michailidis E. (2020). Functional interrogation of a SARS-CoV-2 host protein interactome identifies unique and shared coronavirus host factors. Cell Host Microbe.

[46] Brown S.T., Kelly K.F., Daniel J.M., A C. (2009). Nurse. Hypoxia inducible factor (HIF)-2 alpha is required for the development of the catecholaminergic phenotype of sympathoadrenal cells. J. Neurochem..

[47] Joshi S., Wollenzien H., Leclerc E., Jarajapu Y.P. (2019). Hypoxic regulation of angiotensin-converting enzyme 2 and Mas receptor in human CD34(+) cells. J. Cell Physiol..

[48] Clarke E.N., Belyaev N.D., Lambert D.W., Turner A.J. (2014). Epigenetic regulation of angiotensin-converting enzyme 2 (ACE2) by SIRT1 under conditions of cell energy stress. Clin. Sci..

[49] Zhang R., Wu Y., Zhao M., Liu C., Zhou L., Shen S., Liao S., Yang K., Li Q., Wan H. (2009). Role of HIF-1alpha in the regulation ACE and ACE2 expression in hypoxic human pulmonary artery smooth muscle cells. Am. J. Physiol. Lung Cell Mol. Physiol..

[50] Park K.T., Han J.K., Kim S.J., Lim Y.H. (2020). Gamma-Aminobutyric Acid Increases Erythropoietin by Activation of Citrate Cycle and Stimulation of Hypoxia-Inducible Factors Expression in Rats. Biomolecules.

[51] Wing P.A.C., Keeley T.P., Zhuang X., Lee J.Y., Prange-Barczynska M., Tsukuda S., Morgan S.B., Harding A.C., Argles I.L.A., Kurlekar S. (2021). Hypoxic and pharmacological activation of HIF inhibits SARS-CoV-2 infection of lung epithelial cells. Cell Rep..

[52] Semenza G.L. (2000). Hypoxia, clonal selection, and the role of HIF-1 in tumor progression. Crit. Rev. Biochem. Mol. Biol..

[53] Moolamalla S.T.R., Balasubramanian R., Chauhan R., Priyakumar U.D., Vinod P.K. (2021). Host metabolic reprogramming in response to SARS-CoV-2 infection: A systems biology approach. Microb. Pathog..

[54] Mustroph J., Hupf J., Hanses F., Evert K., Baier M.J., Evert M., Meindl C., Wagner S., Hubauer U., Pietrzyk G. (2020). Decreased GLUT1/NHE1 RNA expression in whole blood predicts disease severity in patients with COVID-19. ESC Heart Fail..

[55] Yağcı S., Serin E., Acicbe Ö., Zeren M.I., Odabaşı M.S. (2021). The relationship between serum erythropoietin, hepcidin, and haptoglobin levels with disease severity and other biochemical values in patients with COVID-19. Int. J. Lab. Hematol..

[56] Tian M., Liu W., Li X., Zhao P., Shereen M.A., Zhu C., Huang S., Liu S., Yu X., Yue M. (2021). HIF-1alpha promotes SARS-CoV-2 infection and aggravates inflammatory responses to COVID-19. Signal Transduct. Target. Ther..

[57] Lionis C., Karakasiliotis I., Petelos E., Linardakis M., Diamantakis A., Symvoulakis E., Panopoulou M., Kampa M., Pirintsos S.A., Sourvinos G. (2021). A mixture of essential oils from three Cretan Aromatic Plants (thyme, Greek sage and Cretan dittany, CAPeo) inhibitsits SARS-CoV-2 proliferation: In vitro evidence and a Proof of Concept intervention study in mild ambulatory COVID-19-positive patients. medRxiv.

[58] Reed L.J., Muench H. (1938). A simple method of estimating fifty per cent endpoints. Am. J. Epidemiol..

[59] Mitjavila M.T., le Couedic J.P., Casadevall N., Navarro S., Villeval J.L., Dubart A., Vainchenker W. (1991). Autocrine stimulation by erythropoietin and autonomous growth of human erythroid leukemic cells in vitro. J. Clin. Investig..

[60] Jia H.P., Look D.C., Shi L., Hickey M., Pewe L., Netland J., Farzan M., Wohlford-Lenane C., Perlman S., McCray P.B. (2005). ACE2 receptor expression and severe acute respiratory syndrome coronavirus infection depend on differentiation of human airway epithelia. J. Virol..

[61] Arnaud N., Dabo S., Akazawa D., Fukasawa M., Shinkai-Ouchi F., Hugon J., Wakita T., Meurs F.E. (2011). Hepatitis C virus reveals a novel early control in acute immune response. PLoS Pathog..

[62] Adamopoulos P.G., Tsiakanikas P., Kontos C.K., Panagiotou A., Vassilacopoulou D., Scorilas A. (2019). Identification of novel alternative splice variants of the human L-DOPA decarboxylase (DDC) gene in human cancer cells, using high-throughput sequencing approaches. Gene.

[63] Livak K.J., Schmittgen T.D. (2001). Analysis of relative gene expression data using real-time quantitative PCR and the 2(-Delta Delta C(T)) Method. Methods.

[64] Dopamine ELISA (IBL Instructions for Use). https://www.ibl-international.com/media/mageworx/downloads/attachment/file/r/e/re59161_ifu_eu_en_dopamine_elisa_2014-12_sym9.pdf.

[65] Zhou Y., Liu Y., Gupta S., Paramo M.I., Hou Y., Mao C., Luo Y., Judd J., Wierbowski S., Bertolotti M. (2022). A comprehensive SARS-CoV-2-human protein-protein interactome reveals COVID-19 pathobiology and potential host therapeutic targets. Nat. Biotechnol..

[66] Terracciano R., Preiano M., Fregola A., Pelaia C., Montalcini T., Savino R. (2021). Mapping the SARS-CoV-2-Host Protein-Protein Interactome by Affinity Purification Mass Spectrometry and Proximity-Dependent Biotin Labeling: A Rational and Straightforward Route to Discover Host-Directed Anti-SARS-CoV-2 Therapeutics. Int. J. Mol. Sci..

[67] Gioutlakis A., Klapa M.I., Moschonas N.K. (2017). PICKLE 2.0: A human protein-protein interaction meta-database employing data integration via genetic information ontology. PLoS ONE.

[68] (2022). EPCAM Single Cell Expression, The Human Protein Atlas. https://www.proteinatlas.org/ENSG00000119888-EPCAM/single+cell+type.

[69] (2022). CD45/PTPRC Single Cell Expression, The Human Protein Atlas. https://www.proteinatlas.org/ENSG00000081237-PTPRC/single+cell+type.

[70] Ziegler C.G.K., Miao V.N., Owings A.H., Navia A.W., Tang Y., Bromley J.D., Lotfy P., Sloan M., Laird H., Williams H.B. (2021). Impaired local intrinsic immunity to SARS-CoV-2 infection in severe COVID-19. Cell.

[71] Jin M., Shi N., Wang M., Shi C., Lu S., Chang Q., Sha S., Lin Y., Chen Y., Zhou H. (2020). CD45: A critical regulator in immune cells to predict severe and non-severe COVID-19 patients. Aging.

[72] Rothschild A.J., Langlais P.J., Schatzberg A.F., Walsh F.X., Cole J.O., Bird E.D. (1984). Dexamethasone increases plasma free dopamine in man. J. Psychiatr. Res..

[73] Blanco-Melo D., Nilsson-Payant B.E., Liu W.C., Uhl S., Hoagland D., Moller R., Jordan T.X., Oishi K., Panis M., Sachs D. (2020). Imbalanced Host Response to SARS-CoV-2 Drives Development of COVID-19. Cell.

[74] Li Y., Li C., Xue P., Zhong B., Mao A.P., Ran Y., Chen H., Wang Y.Y., Yang F., Shu H.B. (2009). ISG56 is a negative-feedback regulator of virus-triggered signaling and cellular antiviral response. Proc. Natl. Acad. Sci. USA.

[75] Lei X., Dong X., Ma R., Wang W., Xiao X., Tian Z., Wang C., Wang Y., Li L., Ren L. (2020). Activation and evasion of type I interferon responses by SARS-CoV-2. Nat. Commun..

[76] Ramasamy S., Subbian S. (2021). Critical Determinants of Cytokine Storm and Type I Interferon Response in COVID-19 Pathogenesis. Clin. Microbiol. Rev..

[77] Chiale C., Greene T.T., Zuniga E.I. (2022). Interferon induction, evasion, and paradoxical roles during SARS-CoV-2 infection. Immunol. Rev..

[78] Matt S.M., Gaskill P.J. (2020). Where Is Dopamine and how do Immune Cells See it?: Dopamine-Mediated Immune Cell Function in Health and Disease. J. Neuroimmune Pharmacol..

[79] Tunbridge E.M., Narajos M., Harrison C.H., Beresford C., Cipriani A., Harrison P.J. (2019). Which Dopamine Polymorphisms Are Functional? Systematic Review and Meta-analysis of COMT, DAT, DBH, DDC, DRD1-5, MAOA, MAOB, TH, VMAT1, and VMAT2. Biol. Psychiatry.

[80] Papatsirou M., Adamopoulos P.G., Artemaki P.I., Georganti V.P., Scorilas A., Vassilacopoulou D., Kontos C.K. (2021). Next-generation sequencing reveals alternative L-DOPA decarboxylase (DDC) splice variants bearing novel exons, in human hepatocellular and lung cancer cells. Gene.

[81] Long W., Yang J., Li Z., Li J., Chen S., Chen D., Wang S., Li Q., Hu D., Huang J. (2021). Abnormal Fibrinogen Level as a Prognostic Indicator in Coronavirus Disease Patients: A Retrospective Cohort Study. Front. Med..

[82] Sui J., Noubouossie D.F., Gandotra S., Cao L. (2021). Elevated Plasma Fibrinogen Is Associated With Excessive Inflammation and Disease Severity in COVID-19 Patients. Front. Cell Infect. Microbiol..

[83] Rostami M., Khoshnegah Z., Mansouritorghabeh H. (2021). Hemostatic System (Fibrinogen Level, D-Dimer, and FDP) in Severe and Non-Severe Patients With COVID-19: A Systematic Review and Meta-Analysis. Clin. Appl. Thromb. Hemost..

[84] El Horri M., Touati S.N., Khachaa I., Chikh Khelifa A., Berrah A., Benmahdi L., Belakehal S.E. (2021). Fibrinogen and D-dimer Are Significantly Associated with Severity of COVID-19 Disease. Res. Pract. Thromb. Haemost..

[85] Szarpak L., Ruetzler K., Safiejko K., Hampel M., Pruc M., Kanczuga-Koda L., Filipiak K.J., Jaguszewski M.J. (2021). Lactate dehydrogenase level as a COVID-19 severity marker. Am. J. Emerg. Med..

[86] Akdogan D., Guzel M., Tosun D., Akpinar O. (2021). Diagnostic and early prognostic value of serum CRP and LDH levels in patients with possible COVID-19 at the first admission. J. Infect. Dev. Ctries..

[87] Li C., Ye J., Chen Q., Hu W., Wang L., Fan Y., Lu Z., Chen J., Chen Z., Chen S. (2020). Elevated Lactate Dehydrogenase (LDH) level as an independent risk factor for the severity and mortality of COVID-19. Aging.

[88] Balachandran S., Roberts P.C., Kipperman T., Bhalla K.N., Compans R.W., Archer D.R., Barber G.N. (2000). Alpha/beta interferons potentiate virus-induced apoptosis through activation of the FADD/Caspase-8 death signaling pathway. J. Virol..

[89] Balachandran S., Kim C.N., Yeh W.C., Mak T.W., Bhalla K., Barber G.N. (1998). Activation of the dsRNA-dependent protein kinase, PKR, induces apoptosis through FADD-mediated death signaling. EMBO J..

[90] Bader S.M., Cooney J.P., Pellegrini M., Doerflinger M. (2022). Programmed cell death: The pathways to severe COVID-19?. Biochem. J..

[91] Danta C.C. (2021). SARS-CoV-2, Hypoxia, and Calcium Signaling: The Consequences and Therapeutic Options. ACS Pharmacol. Transl. Sci..

[92] Veleri S. (2022). Neurotropism of SARS-CoV-2 and neurological diseases of the central nervous system in COVID-19 patients. Exp. Brain Res..

[93] (2022). FGB Gene (Fibrinogen), Genecards. https://www.genecards.org/cgi-bin/carddisp.pl?gene=FGB.

[94] Helms J., Tacquard C., Severac F., Leonard-Lorant I., Ohana M., Delabranche X., Merdji H., Clere-Jehl R., Schenck M., Fagot Gandet F. (2020). High risk of thrombosis in patients with severe SARS-CoV-2 infection: A multicenter prospective cohort study. Intensive Care Med..

[95] Vassilaki N., Frakolaki E. (2017). Virus-host interactions under hypoxia. Microbes Infect..

[96] Gassmann M., Heinicke K., Soliz J., Ogunshola O.O. (2003). Non-erythroid functions of erythropoietin. Adv. Exp. Med. Biol..

[97] Ghezzi P., Brines M. (2004). Erythropoietin as an antiapoptotic, tissue-protective cytokine. Cell Death Differ..

[98] Li S., Zhang Y., Guan Z., Li H., Ye M., Chen X., Shen J., Zhou Y., Shi Z.L., Zhou P. (2020). SARS-CoV-2 triggers inflammatory responses and cell death through caspase-8 activation. Signal Transduct. Target. Ther..

[99] Begemann M., Gross O., Wincewicz D., Hardeland R., Gastaldi V.D., Vieta E., Weissenborn K., Miskowiak K.W., Moerer O., Ehrenreich H. (2021). Addressing the ‘hypoxia paradox’ in severe COVID-19: Literature review and report of four cases treated with erythropoietin analogues. Mol. Med..

[100] Ehrenreich H., Weissenborn K., Begemann M., Busch M., Vieta E., Miskowiak K.W. (2020). Erythropoietin as candidate for supportive treatment of severe COVID-19. Mol. Med..

[101] Hadadi A., Mortezazadeh M., Kolahdouzan K., Alavian G. (2020). Does recombinant human erythropoietin administration in critically ill COVID-19 patients have miraculous therapeutic effects?. J. Med. Virol..

[102] Sahebnasagh A., Mojtahedzadeh M., Najmeddin F., Najafi A., Safdari M., Ghaleno H.R., Habtemariam S., Berindan-Neagoe I., Nabavi S.M. (2020). A Perspective on Erythropoietin as a Potential Adjuvant Therapy for Acute Lung Injury/Acute Respiratory Distress Syndrome in Patients with COVID-19. Arch. Med. Res..

[103] Urbanska K., Orzechowski A. (2019). Unappreciated Role of LDHA and LDHB to Control Apoptosis and Autophagy in Tumor Cells. Int. J. Mol. Sci..

[104] Hamed E.A., Sayyed H.G., Abbas A.M., Gaber M.M.A., Aleem H. (2022). Nesfatin-1, Dopamine, and NADPH levels in Infertile Women with Polycystic Ovary Syndrome: Is There a Relationship Between Their Levels and Metabolic and Hormonal Variables. J. Reprod. Infertil..

[105] Katar M., Deveci H., Deveci K. (2022). Evaluation of clinical relationship of serum niacin and dopamine levels in patients with fibromyalgia syndrome. Turk. J. Phys. Med. Rehabil..

[106] Kim J., Jung H., Choi J.Y., Lee J.W., Yoon M. (2022). Plasma concentration of dopamine varies depending on breed, sex, and the genotype of DRD4 in horses. J. Anim. Sci. Technol..

[107] Bolton J.L., Trush M.A., Penning T.M., Dryhurst G., Monks T.J. (2000). Role of quinones in toxicology. Chem. Res. Toxicol..

[108] Tian Y., Jiang W., Gao N., Zhang J., Chen W., Fan D., Zhou D., An J. (2010). Inhibitory effects of glutathione on dengue virus production. Biochem. Biophys. Res. Commun..

