# Supplementary material for: Significance of Catecholamine Biosynthetic/Metabolic Pathway in SARS-CoV-2 Infection and COVID-19 Severity
Source: Cells. 2022 Dec 20;12(1):12. doi: 10.3390/cells12010012 (PMC9818320; doi:10.3390/cells12010012)
Supplement: Supplementary file 1 [file cells-12-00012-s001.zip › cells-2050602-supplementary/Mpekoulis et. al Supplementary Files Revision 9.12.22.pdf]

cells

MDPI

Supplementary Data

Significance of Catecholamine biosynthetic/metabolic pathway in SARS-CoV-2 infection and COVID-19 severity

George Mpekoulis [1], Katerina I. Kalliampakou[1], Rafaela S. Milona[1], Despoina Lagou[1], Anastasios Ioannidis[2], Edison Jajaj[3], Christos T. Chasapis[4], Dionysios Kefallinos[5], Ioannis Karakasiliotis[6], Anastasia Kotanidou[3], Stylianos Chatzipanagiotou[7], Dido Vassilacopoulou[8], Alice G. Vassiliou[3,#], Emmanouil Angelakis[9,10,#], Niki Vassilaki[1,*]

1 Laboratory of Molecular Virology, Hellenic Pasteur Institute, Athens 11521, Greece
2 Department of Nursing, University of Peloponnese, Sparti 23100, Greece
3 First Department of Critical Care Medicine & Pulmonary Services, GP Livanos and M Simou Laboratories, National and Kapodistrian University of Athens Medical School, Evangelismos Hospital, Athens 10676, Greece
4 Institute of Chemical Biology, National Hellenic Research Foundation, 11635 Athens, Greece
5 School of Electrical Engineering and Computer Science, National Technical University of Athens, 9 Iroon Polytechniou Street, Zografou, Athens 15773, Greece
6 Laboratory of Biology, Department of Medicine, Democritus University of Thrace, Alexandroupolis 68100, Greece
7 Department of Medical Biopathology, Medical School, University of Athens, Eginition Hospital, Athens 11528, Greece
8 Section of Biochemistry and Molecular Biology, Faculty of Biology, National and Kapodistrian University of Athens, Athens 15772, Greece
9 Department of Diagnostics, Hellenic Pasteur Institute, Athens 11521, Greece
10 Aix Marseille Univ, IRD, IHU Méditerranée Infection, VITROME, Marseille 13005, France
# Equal contribution
* Corresponding author: nikiv@pasteur.gr (N.V.)

Citation: Mpekoulis, G.; Kalliampakou, K.I.; Milona, R.S.; Lagou, D.; Ioannidis, A.; Jahaj, E.; Chasapis, C.T.; Kefallinos, D.; Karakasiliotis, I.; Kotanidou, A.; et al. Significance of Catecholamine Biosynthetic/ Metabolic Pathway in SARS-CoV-2 Infection and COVID-19 Severity. *Cells* **2023**, *12*, 12. https://doi.org/ 10.3390/cells12010012

Academic Editors: Mara Biasin and Fiona Limanaqi

Received: 7 November 2022
Revised: 11 December 2022
Accepted: 13 December 2022
Published: 20 December 2022

Copyright: © 2022 by the authors. Licensee MDPI, Basel, Switzerland. This article is an open access article distributed under the terms and conditions of the Creative Commons Attribution (CC BY) license (https://creativecommons.org/licenses/by/4.0/).

Supplementary Tables

Table S1. Mean values of the studied genes' expression in nasopharyngeal tissue of SARS-CoV-2 positive or negative subjects.

		SARS-CoV-2	
**Characteristics**	**Negative**	**Mild- CoV-2**	**Severe-CoV-2**
***DDC* (2^-ΔΔCt)**	0.004 ± 0.002	0.033 ± 0.019	0.002 ± 0.0015
***ACE2* (2^-ΔΔCt)**	0.005 ± 0.003	0.008 ± 0.005	0.001 ± 0.002
***dACE2* (2^-ΔΔCt)**	0.008 ± 0.003	0.023 ± 0.008	0.021 ± 0.008
***ISG56* (2^-ΔΔCt)**	0.561 ± 0.225	1.414 ± 1.014	0.668 ± 0.318
***EPO* (2^-ΔΔCt)**	0.011 ± 0.004	0.027 ± 0.011	0.027 ± 0.013

*The 2^-ΔΔCt mean value for each gene was used to evaluate the fold-differences between the studied groups.

Table S2. Demographic data of individuals whose blood samples were used.

Characteristics	Healthy	ICU/CoV-2	ICU/non-CoV-2	p-value
**Total number**	n=35	n=32	n=30	
**Median age in years (IQR)**	49 (42-55)	57 (62-70)	57 (35 - 73)	0.0024[a]
**Sex**				
**Male**	20 (57.2%)	25 (78.1%)	17 (78.1%)	0.1437[b]
**Female**	15 (42.8%)	7 (21.9%)	13 (21.9%)	

[a]: p-value was calculated with Kruskal-Wallis test, followed by Dunn's test.
[b]: p-value was calculated with Chi-square test.

Table S3. Mean values of the studied genes expression in whole blood samples of SARS-CoV-2 positive or negative subjects.

SARS-CoV-2 (Blood samples)				
**Characteristics**		**Healthy**	**ICU/CoV-2**	**Ward/CoV-2**
**Gene expression**				
***DDC* (2^-ΔΔCt)**	Mean ± SD	1.876 + 0.964	0.538 + 0.265	0.246 + 0.243
***dACE2* (2^-ΔΔCt)**	Mean ± SD	0.134 + 0.066	0.041 + 0.0301	0.017 + 0.02
***ISG56* (2^-ΔΔCt)**	Mean ± SD	0.704 + 0.499	0.206 + 0.127	0.438 + 0.411
***MAOA* (2^-ΔΔCt)**	Mean ± SD	0.028 + 0.026	0.427 + 0.315	0.102 + 0.126
***MAOB* (2^-ΔΔCt)**	Mean ± SD	0.007 + 0.006	0.072 + 0.053	0.072 + 0.049
***DBH* (2^-ΔΔCt)**	Mean ± SD	0.038 + 0.025	0.041 + 0.036	0.049 + 0.054
***EPO* (2^-ΔΔCt)**	Mean ± SD	0.688 + 0.498	0.059 + 0.053	0.023 + 0.037

***The 2^-ΔΔCt mean value for each gene was used to evaluate the fold-differences between the studied groups.**

Supplementary Figures

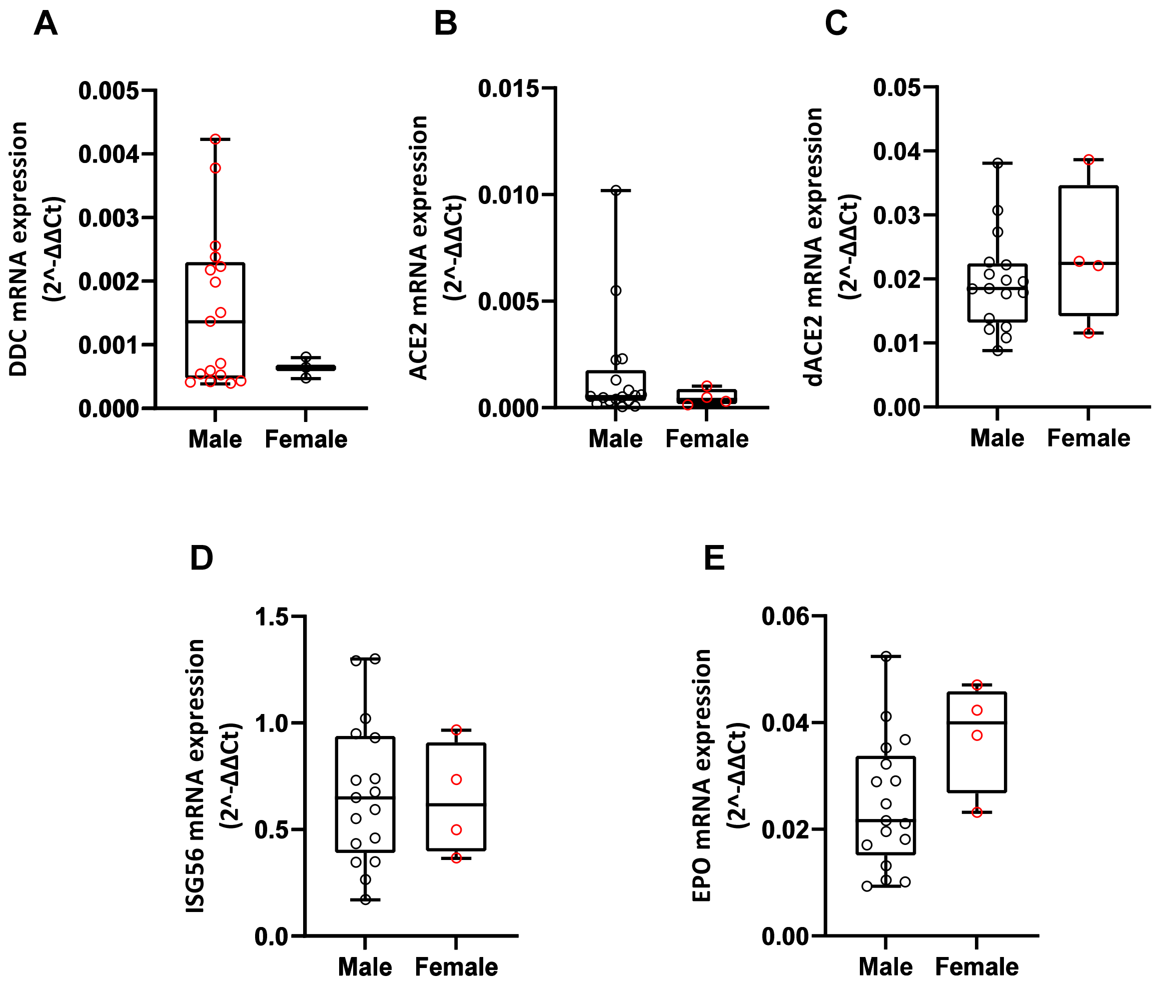

Figure S1. Comparison of *DDC* (**A**), *ACE2* (**B**), *dACE2* (**C**), *ISG56* (**D**), or *EPO* (**E**) expression in the nasopharyngeal swab samples of COVID-19 patients between men and women. Data are displayed as box plots and dots; the line in the middle corresponds to the median value; box edges, 25th to 75th centiles; whiskers, range of values. p-values were calculated with the Mann-Whitney U test.

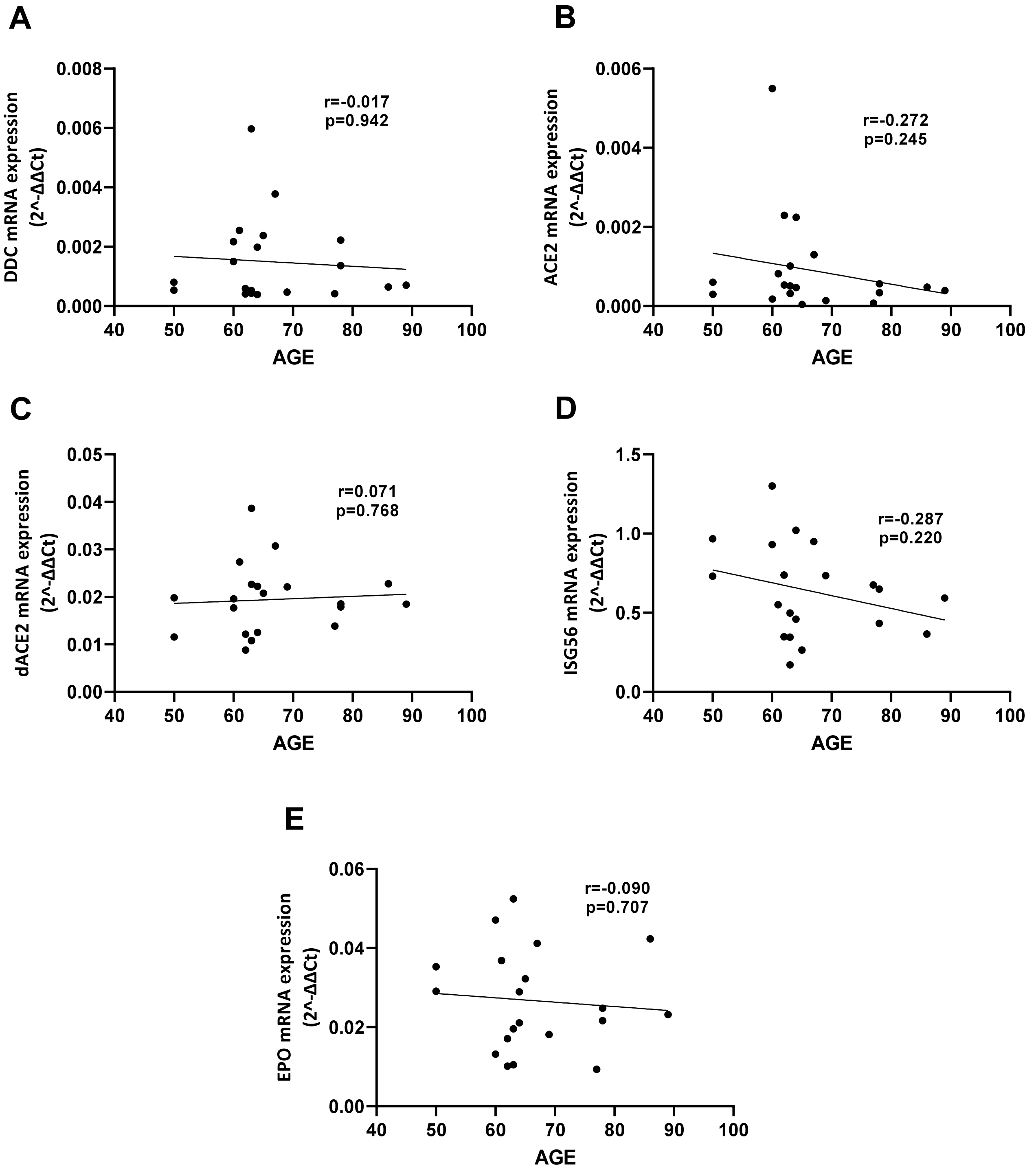

Figure S2. Correlation among the expression of *DDC* (**A**), *ACE2* (**B**), *dACE2* (**C**), *ISG56* (**D**), or *EPO* (**E**) with patients' age (continuous parameter) in nasopharyngeal swab samples of COVID-19 patients with severe symptoms. Data are presented as XY scatter plot with fitted linear regression lines. Pearson's or Spearman's correlation coefficient (r) and p-values (p) were calculated.

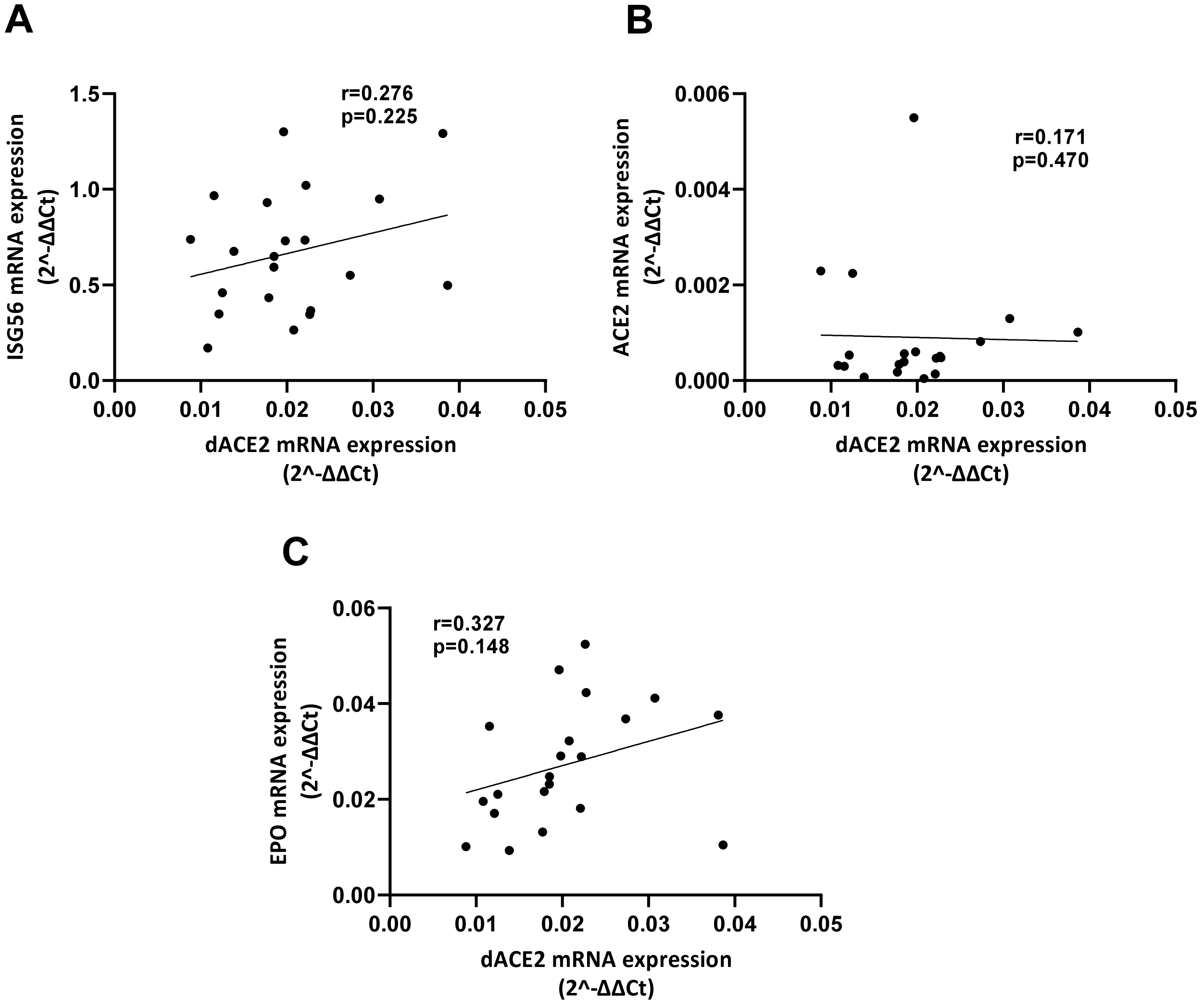

Figure S3. Correlation among the expression of *ISG56* (**A**), *ACE2* (**B**), or *EPO* (**C**), with that of *dACE2* in the nasopharyngeal swab samples of COVID-19 patients with severe symptoms. Data are presented as XY scatter plot with fitted linear regression lines. Pearson's or Spearman's correlation coefficient (r) and p-values (p) were calculated.

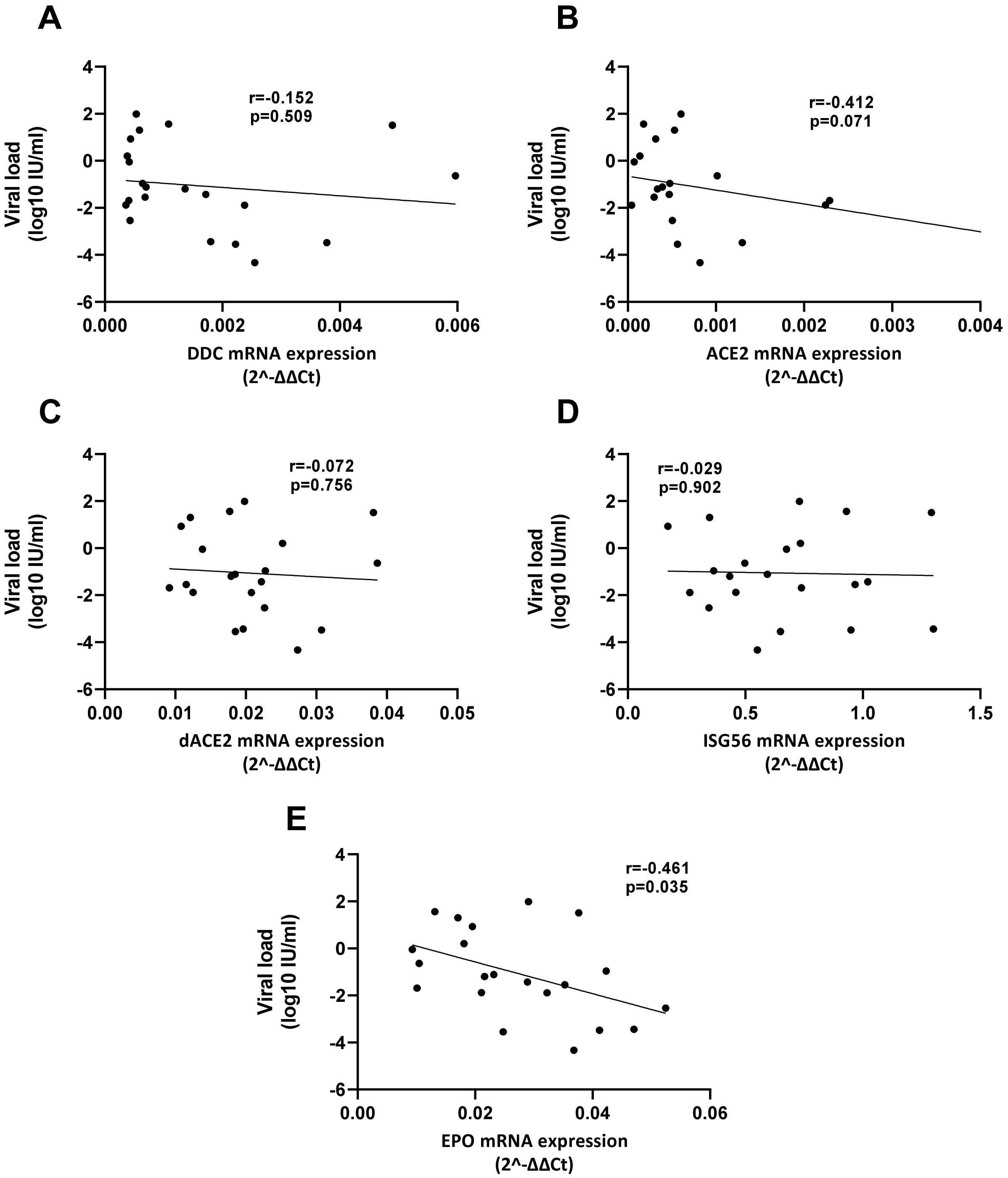

Figure S4. Correlation among the expression of *DDC* (**A**), *ACE2* (**B**), *dACE2* (**C**), *ISG56* (**D**), or *EPO* (**E**) with SARS-CoV-2 viral titers in the nasopharyngeal tissue of severe COVID-19 cases. Data are presented as XY scatter plot with fitted linear regression lines. Pearson's or Spearman's correlation coefficient (r) and p-values (p) were calculated.

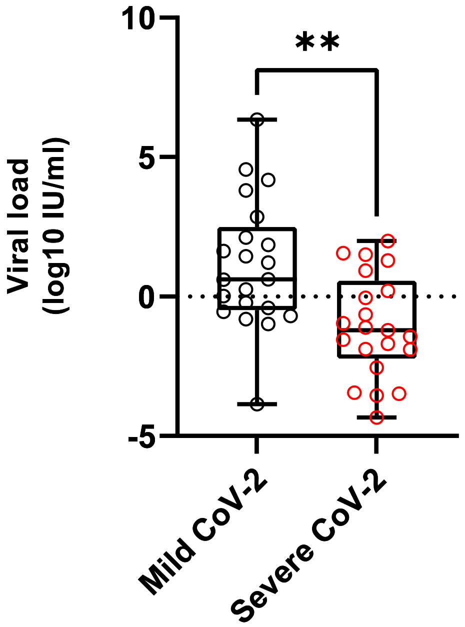

Figure S5. Comparison of SARS-CoV-2 viral load levels between COVID-19 patients with mild or no symptoms with more severe cases. Data are displayed as box plots and dots; the line in the middle corresponds to the median value; box edges, 25th to 75th centiles; whiskers, range of values. p-values were calculated with the unpaired t-test. ** $p=0.0014$.

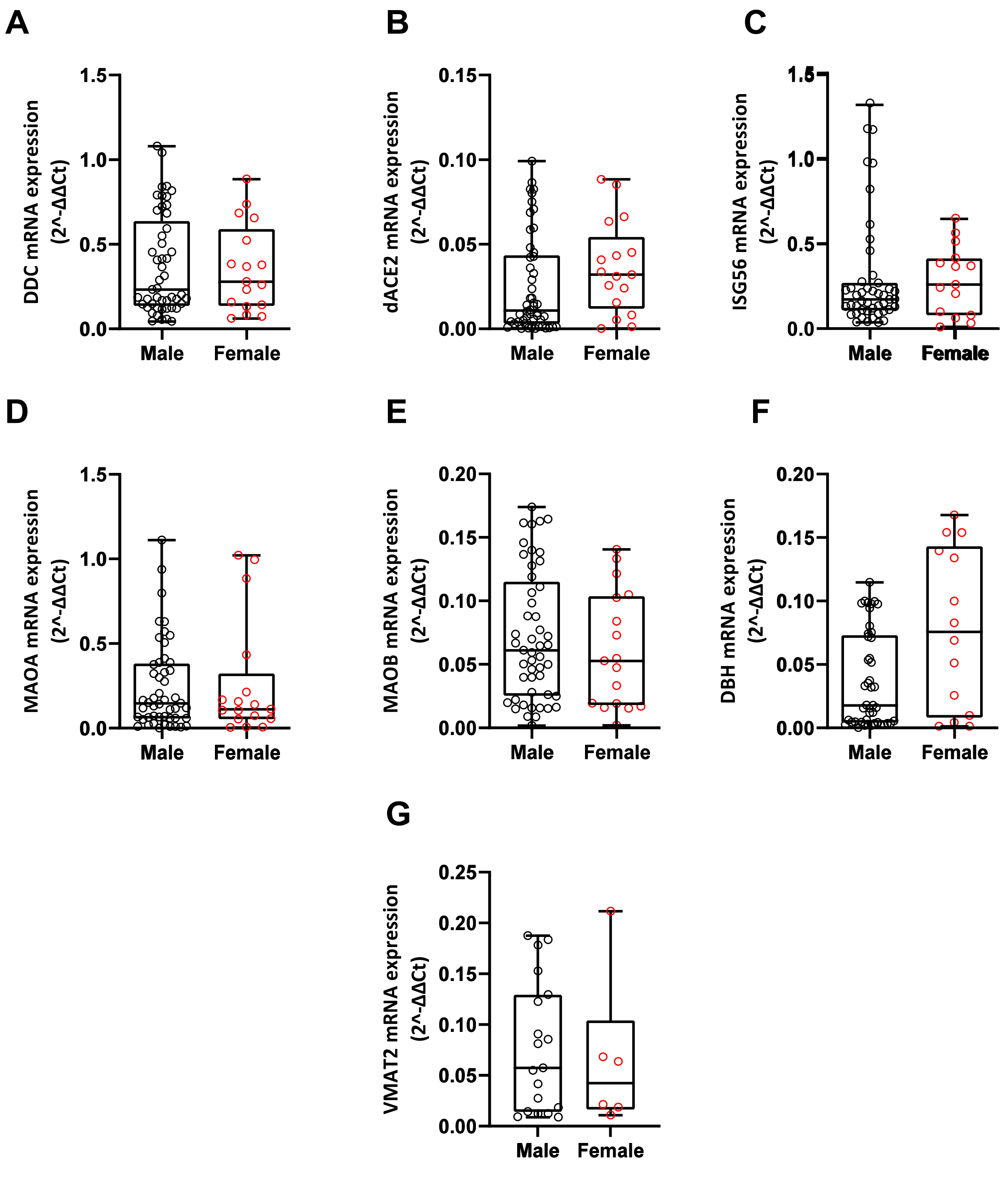

Figure S6. Comparison of *DDC* (**A**), d*ACE2* (**B**), *ISG56* (**C**), *MAOA* (**D**), *MAOB* (**E**), *DBH* (**F**), or *VMAT2* (**G**) expression between men and women, in whole samples of COVID-19 patients with severe symptoms. Data are displayed as box plots and dots; the line in the middle corresponds to the median value; box edges, 25th to 75th centiles; whiskers, range of values. p-values were calculated with the Mann-Whitney U test.

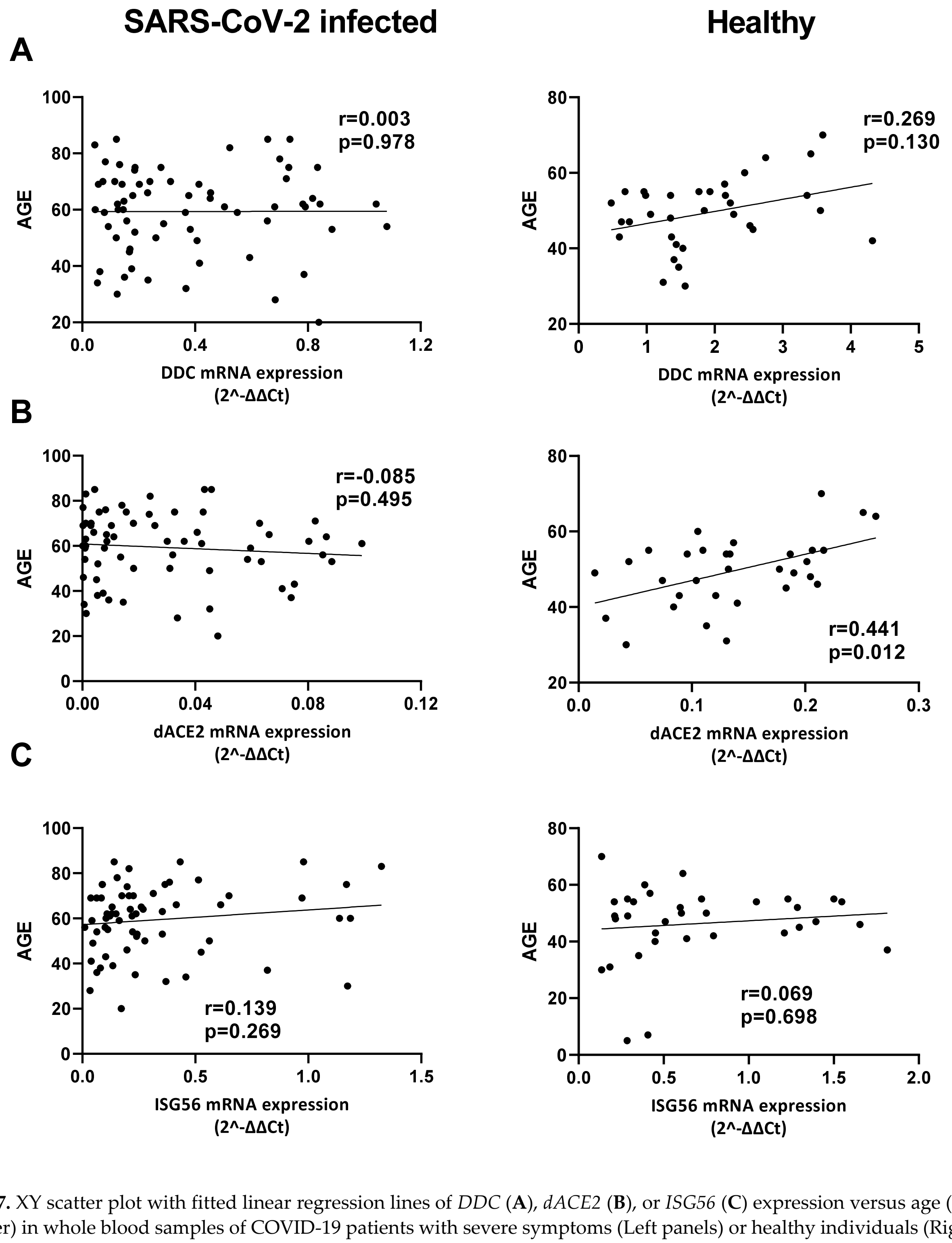

Figure S7. XY scatter plot with fitted linear regression lines of *DDC* (**A**), *dACE2* (**B**), or *ISG56* (**C**) expression versus age (continuous parameter) in whole blood samples of COVID-19 patients with severe symptoms (Left panels) or healthy individuals (Right panels). Pearson's or Spearman's correlation coefficient (r) and p-values (p) were calculated.

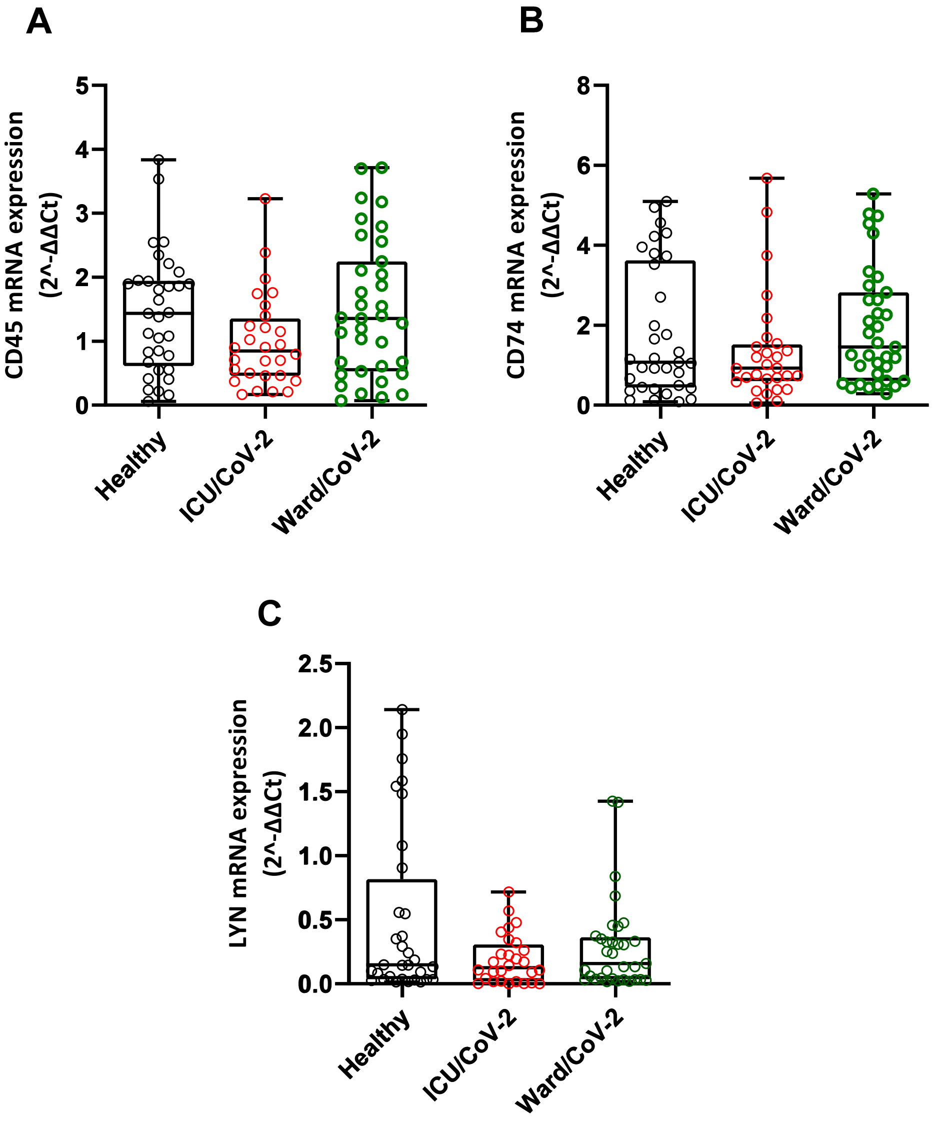

Figure S8. Box plot analysis comparing the mRNA expression of *CD45* **(A)**, *CD74* **(B)** and *LYN* **(C)** in whole blood samples among Healthy individuals and COVID-19 hospitalized patients (ICU/CoV-2, Ward/CoV-2 groups). Data are presented as box plots and dots, line in the middle corresponds for the median; box edges, 25th to 75th centiles; whiskers, range of values. p-values were determined with the non-parametric Kruskal-Wallis test. Multiple comparisons among the studied groups were performed with Dunn's test.

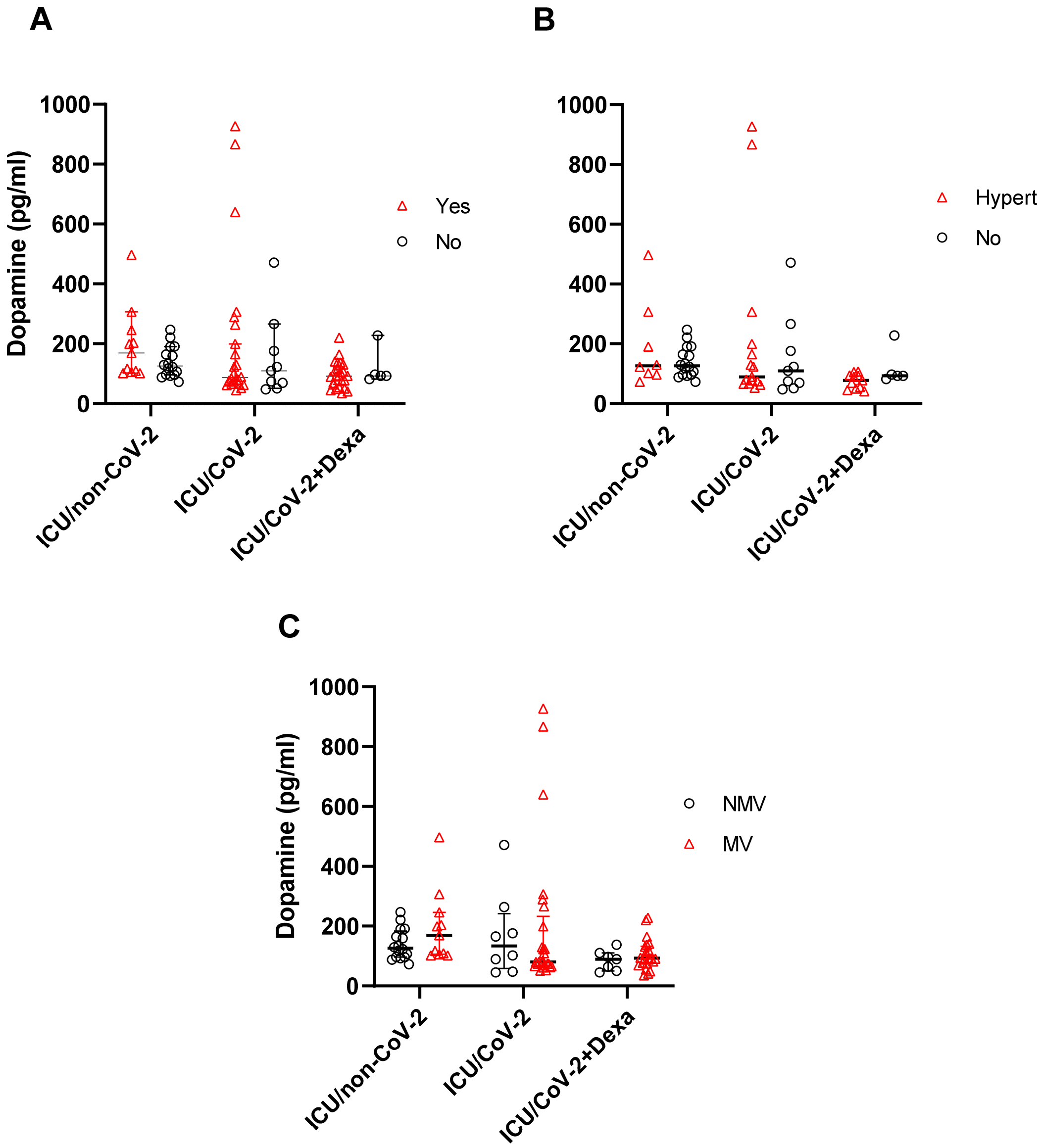

Figure S9. Serum dopamine levels of SARS-CoV-2 infected subjects in the presence of comorbidities. **(A)** Absolute dopamine levels in the serum of ICU COVID-19 (receiving DEXA or not) or ICU non-COVID-19 patients, with (Yes) or without (No) comorbidities. **(B)** Absolute dopamine levels in the same patient cohorts, with (Hypert) or without (No) hypertension. **(C)** Effect of mechanical ventilation on dopamine levels of the aforementioned patient groups. p-values were determined with Two-Way ANOVA.

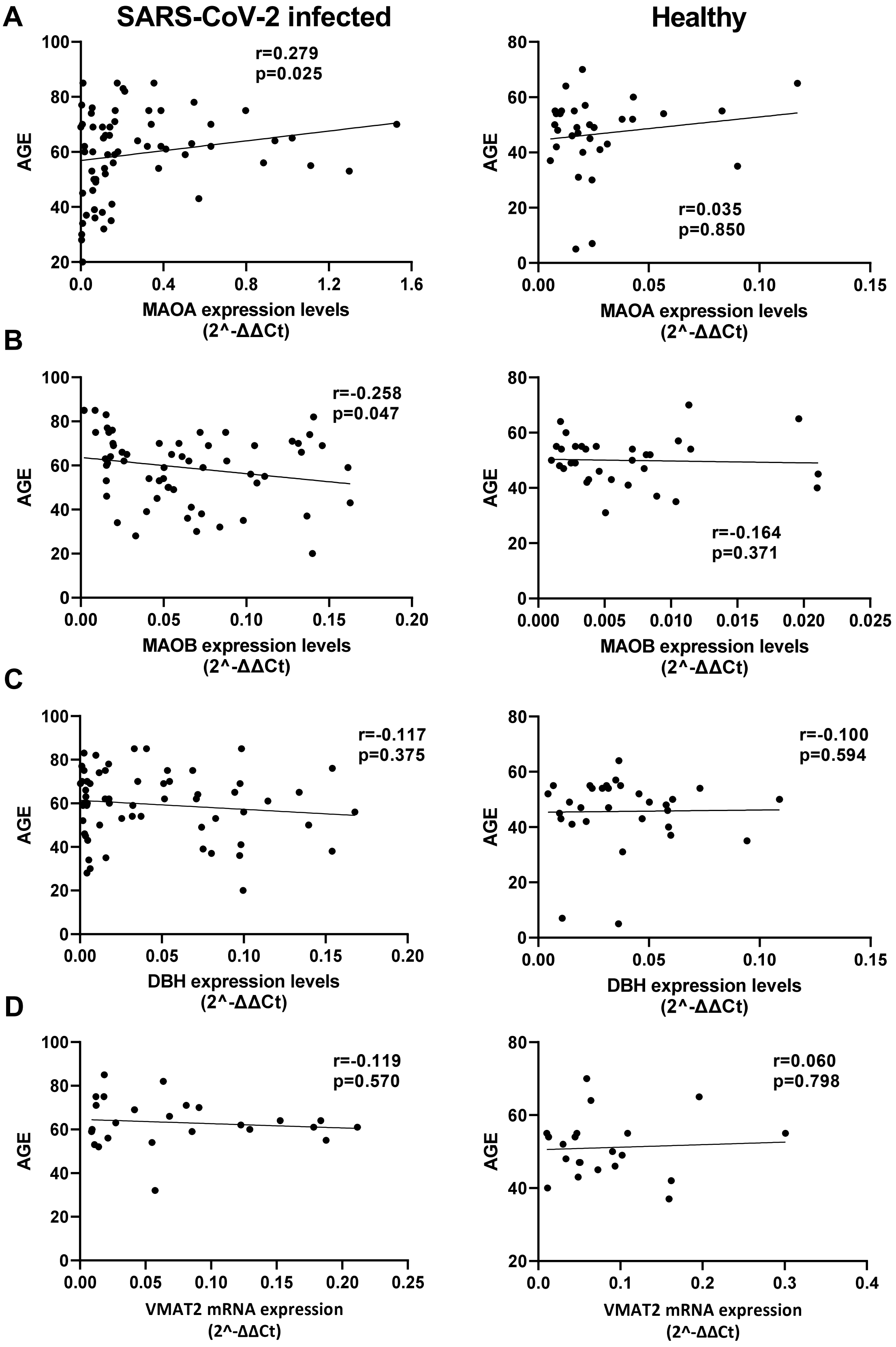

Figure S10. XY scatter plot with fitted linear regression lines of *MAOA* (**A**), *MAOB* (**B**), *DBH* (**C**), or *VMAT2* (**D**) expression versus age (continuous parameter) in whole samples of COVID-19 patients with severe symptoms (Left panels) or healthy individuals (Right panels). Pearson's or Spearman's correlation coefficient (r) and p-values (p) were calculated.

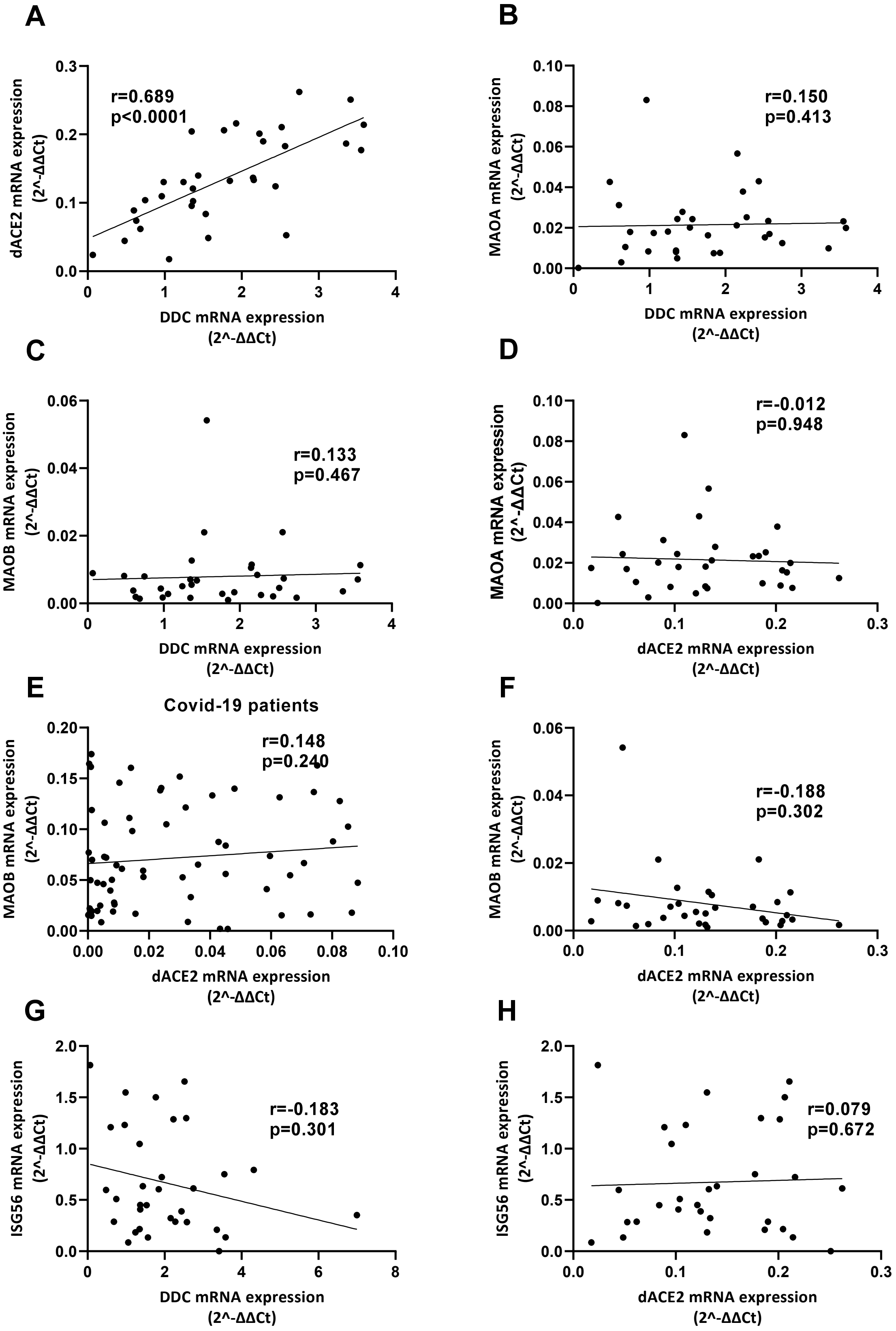

Figure S11. Correlation of the expression of all studied genes in the blood of COVID-19 severe cases and healthy individuals. (**A-C**) Correlation among the expression of *dACE2* (**A**), *MAOA* (**B**), or *MAOB* (**C**), with that of *DDC* in the whole blood samples of healthy individuals. (**D-F**) Correlation among the expression of *MAOA* and *MAOB* with that of *dACE2* in whole blood samples of COVID-19 patients with severe symptoms (**E**), or healthy individuals (**D,F**). (**G,H**) Correlation among the expression of *DDC* or *dACE2* with that

of *ISG56* in the blood of healthy individuals. Data are presented as XY scatter plot with fitted linear regression lines. Pearson's or Spearman's correlation coefficient (r) and p-values (p) were calculated.

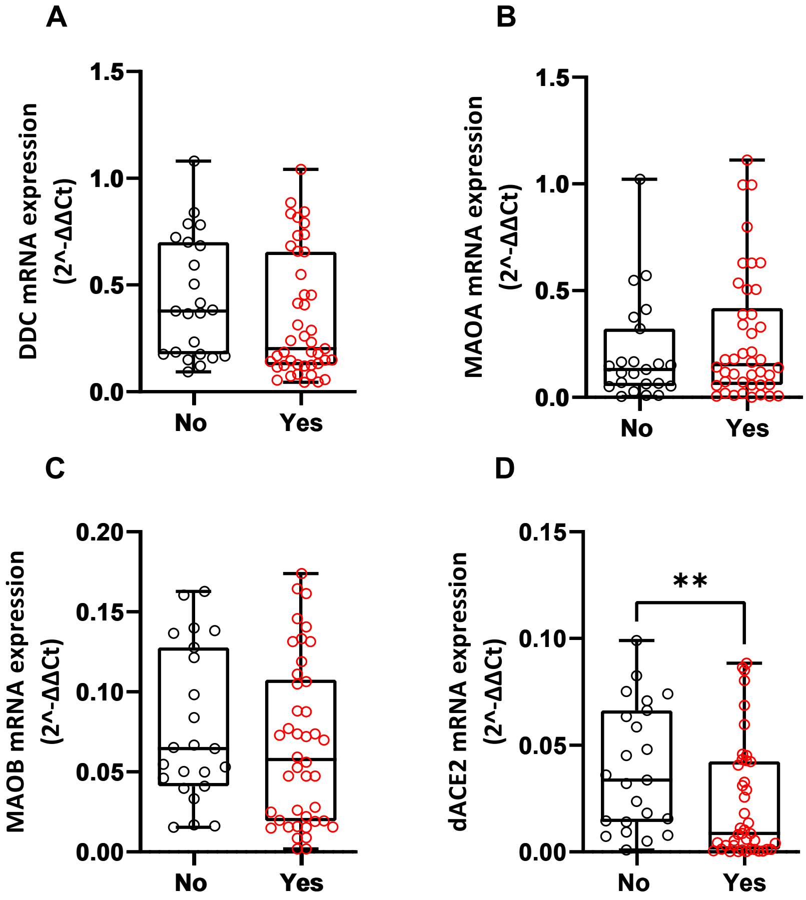

Figure S12. Box plot analysis of the mRNA expression of *DDC* **(A)**, *MAOA* **(B)**, *MAOB* **(C)** and *dACE2* **(D)** in whole blood samples among the hospitalized COVID-19 patients, in the presence (Yes) or absence (No) of comorbidities. Each patient's mRNA expression value is represented as a dot; line in the middle, median; box edges, 25th to 75th centiles; whiskers, range of values. p-values were calculated using the non-parametric Mann-Whitney U test. **p<0.01.

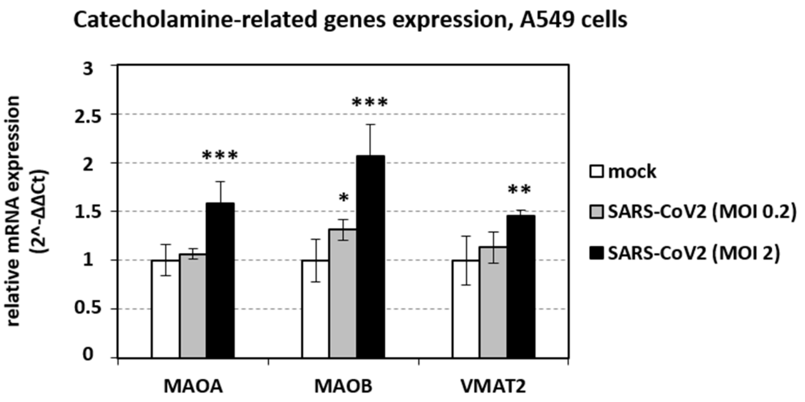

Figure S13. SARS-CoV-2 infection of A549 cells upregulated the expression of *MAOA*, *MAOB*, and *VMAT2* 24 hours post-infection. Further analysis of data derived from the RNA-sequencing Skyline database (http://rstats.immgen.org/Skyline_COVID-19/skyline.html) [67]. * p<0.05, ** p<0.01, ***p<0.0001 vs mock. p values were calculated with Student's t-test.

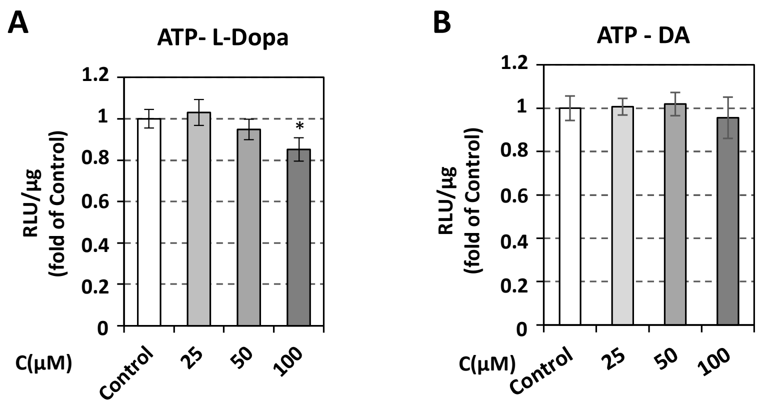

Figure S14. Effect of DDC substrate (L-Dopa, A) and product (dopamine, B) on intracellular ATP levels**.** VeroE6 cells were inoculated with the indicated concentrations of **(A)** L-Dopa, **(B)** DA, or were mock-treated (Control) for 72 h. A chemiluminescence-based assay was performed to determine the intracellular ATP levels, which were expressed as RLU/μg of total protein amount. Values derived from the Control cells were set to one. Bars represent mean values from three independent experiments in triplicates. Error bars indicate standard deviations. *p<0.05 vs Control.
