# Supplementary figures and images for: Significance of Catecholamine Biosynthetic/Metabolic Pathway in SARS-CoV-2 Infection and COVID-19 Severity
Source: Cells. 2022 Dec 20;12(1):12. doi: 10.3390/cells12010012 (PMC9818320; doi:10.3390/cells12010012)

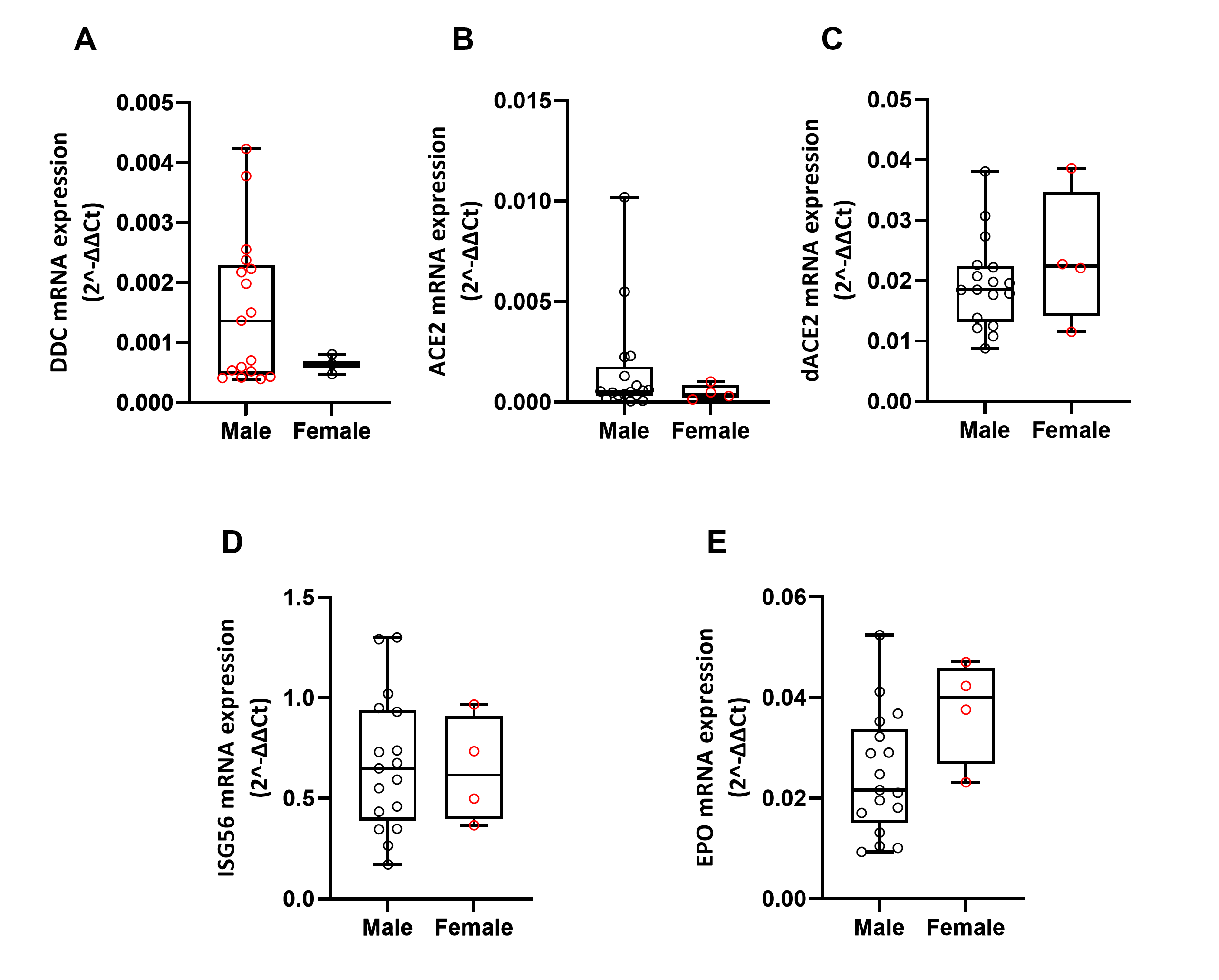

Supplement: Supplementary file 1 [file cells-12-00012-s001.zip › cells-2050602-supplementary/Figure S1.tif]

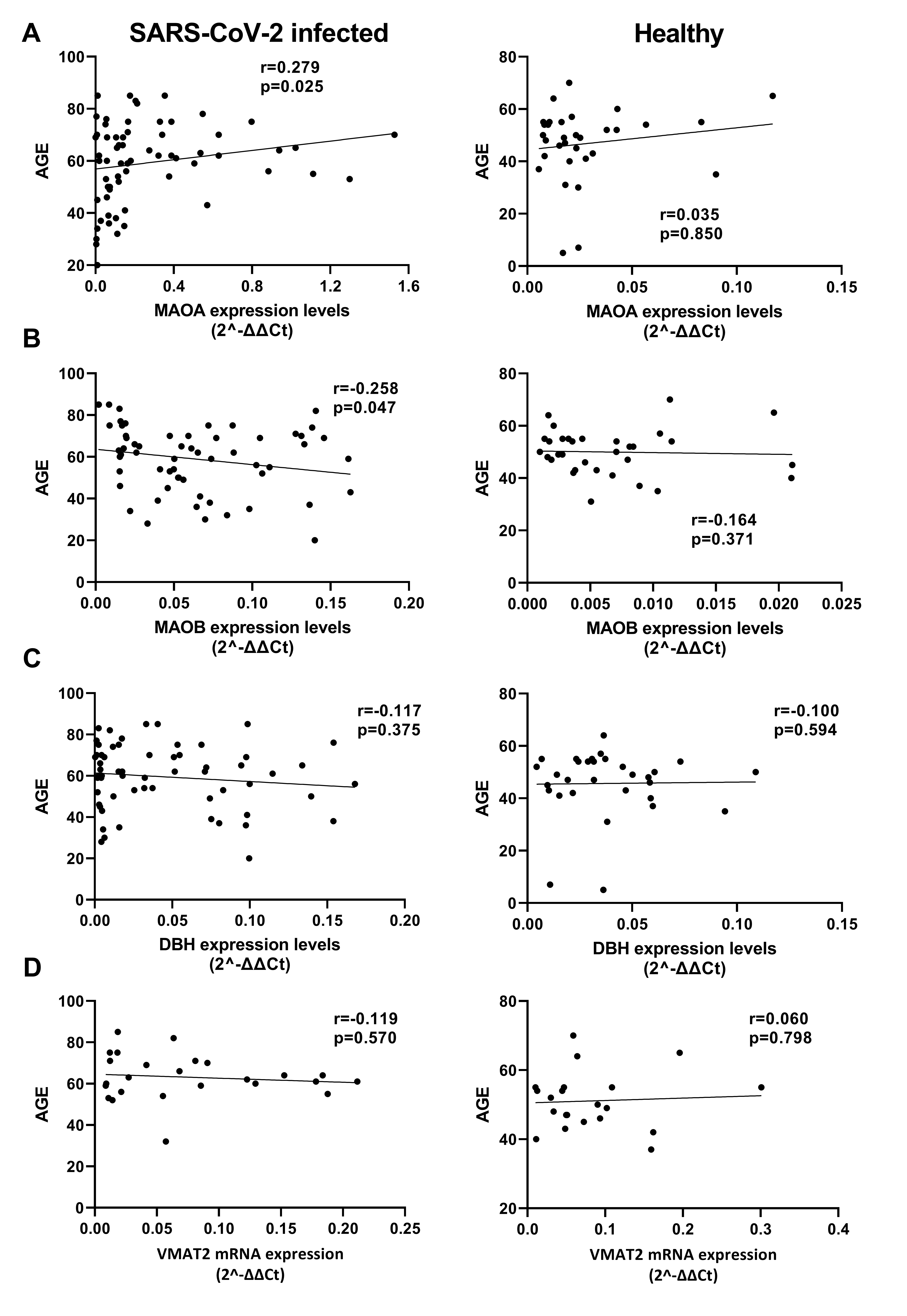

Supplement: Supplementary file 1 [file cells-12-00012-s001.zip › cells-2050602-supplementary/Figure S10.tif]

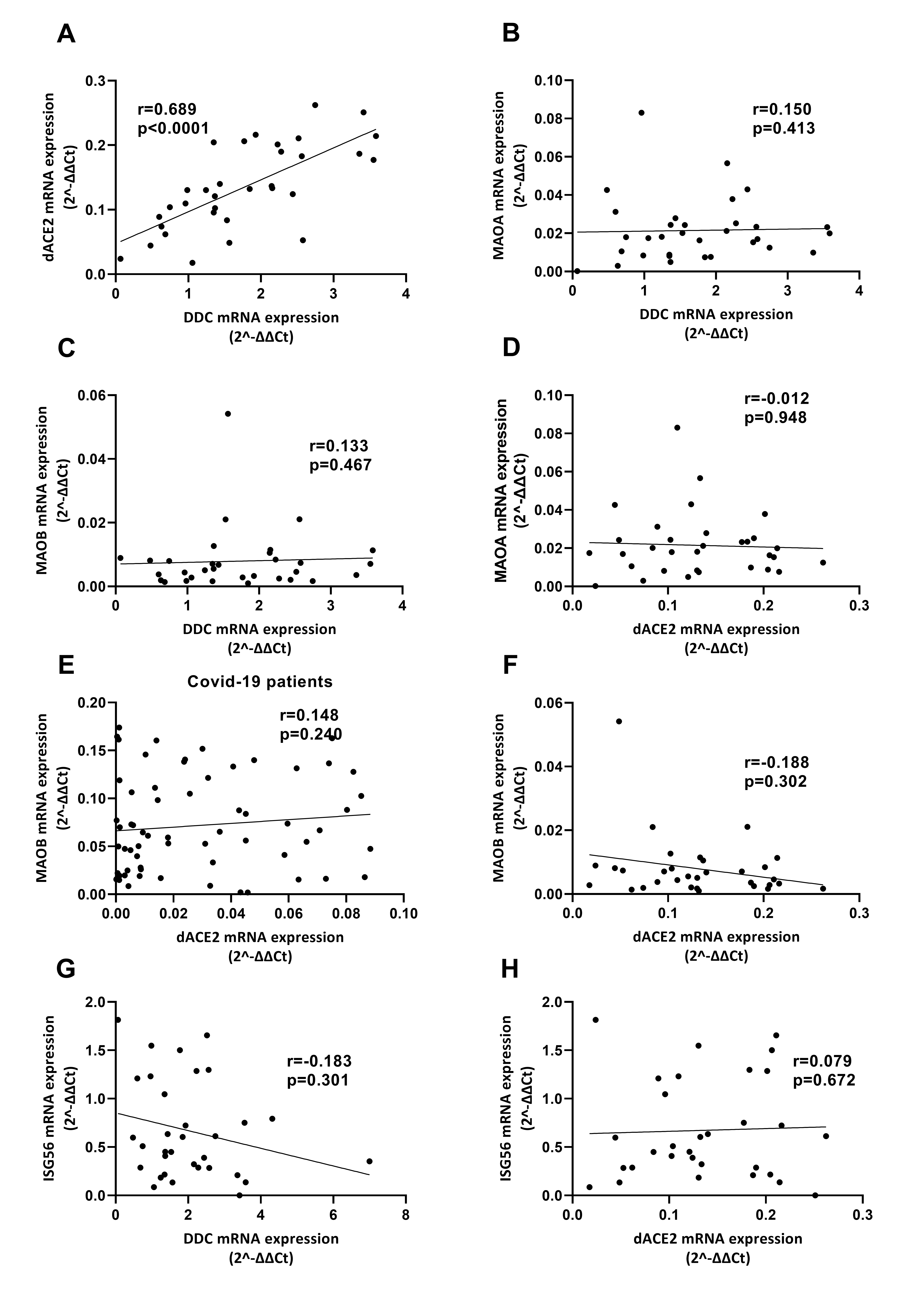

Supplement: Supplementary file 1 [file cells-12-00012-s001.zip › cells-2050602-supplementary/Figure S11.tif]

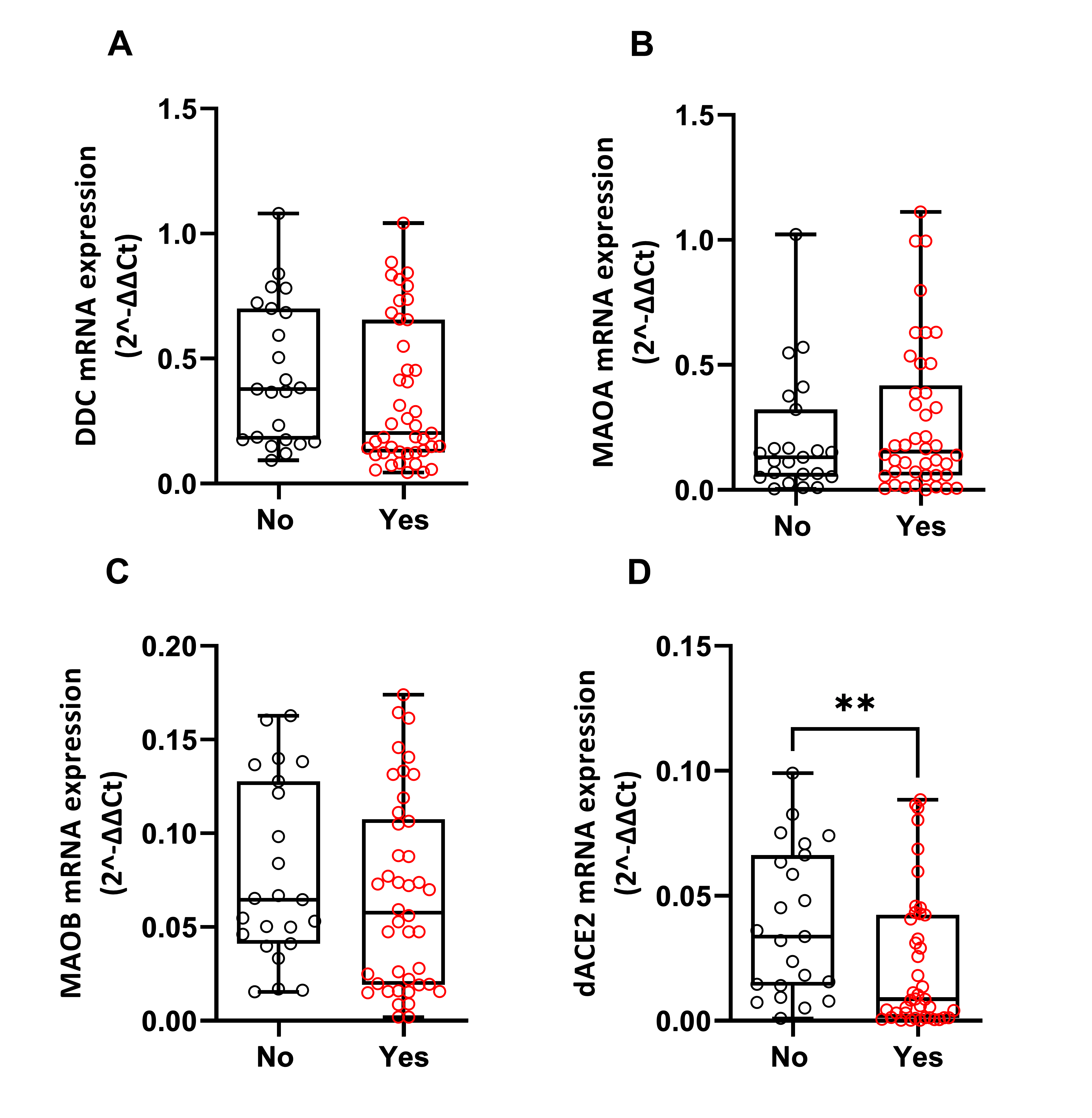

Supplement: Supplementary file 1 [file cells-12-00012-s001.zip › cells-2050602-supplementary/Figure S12.tif]

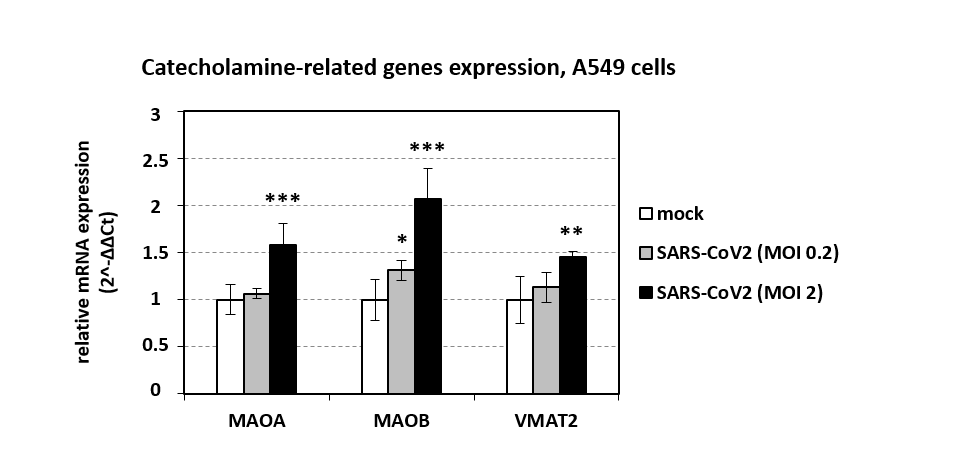

Supplement: Supplementary file 1 [file cells-12-00012-s001.zip › cells-2050602-supplementary/Figure S13.tif]

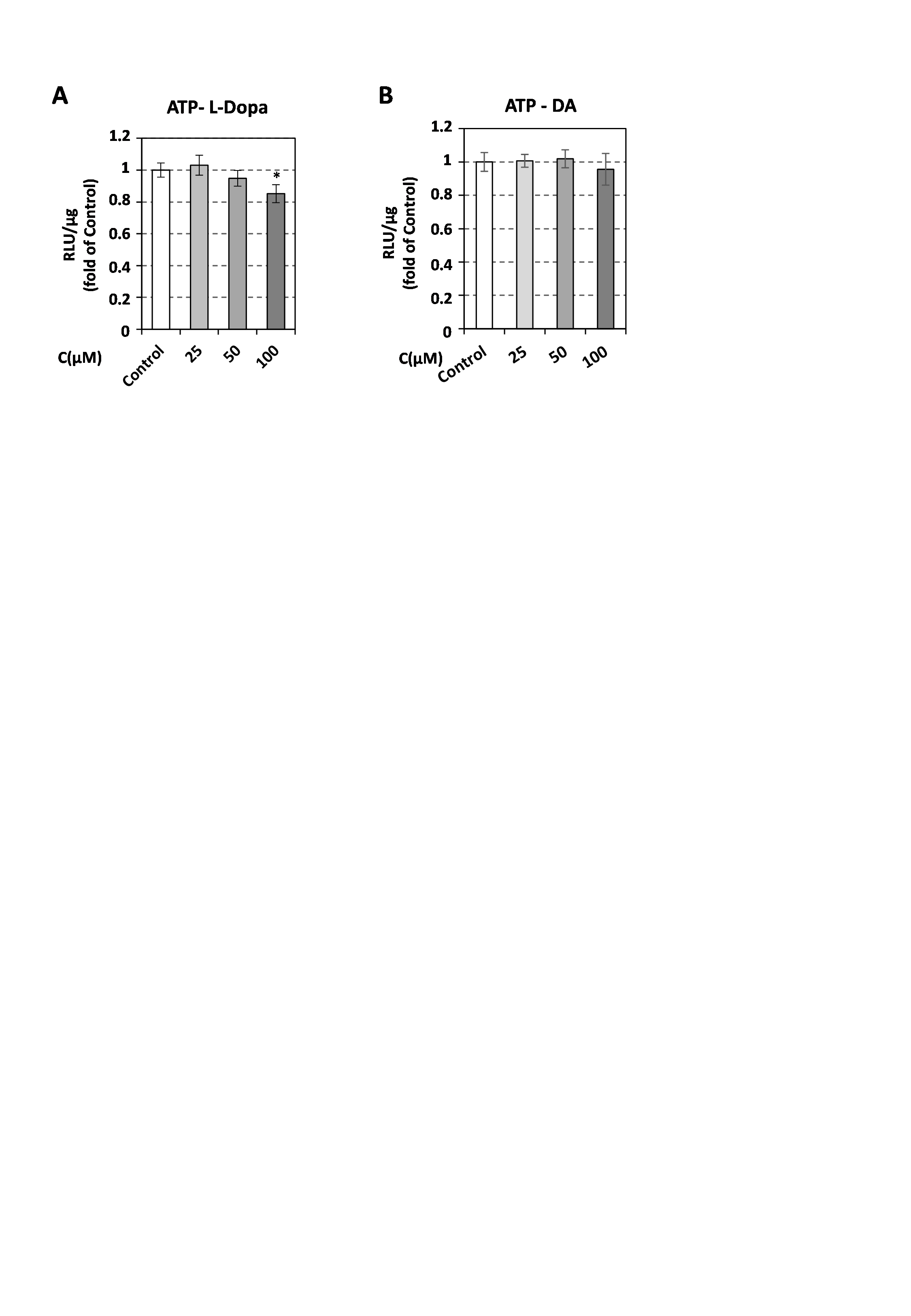

Supplement: Supplementary file 1 [file cells-12-00012-s001.zip › cells-2050602-supplementary/Figure S14.tif]

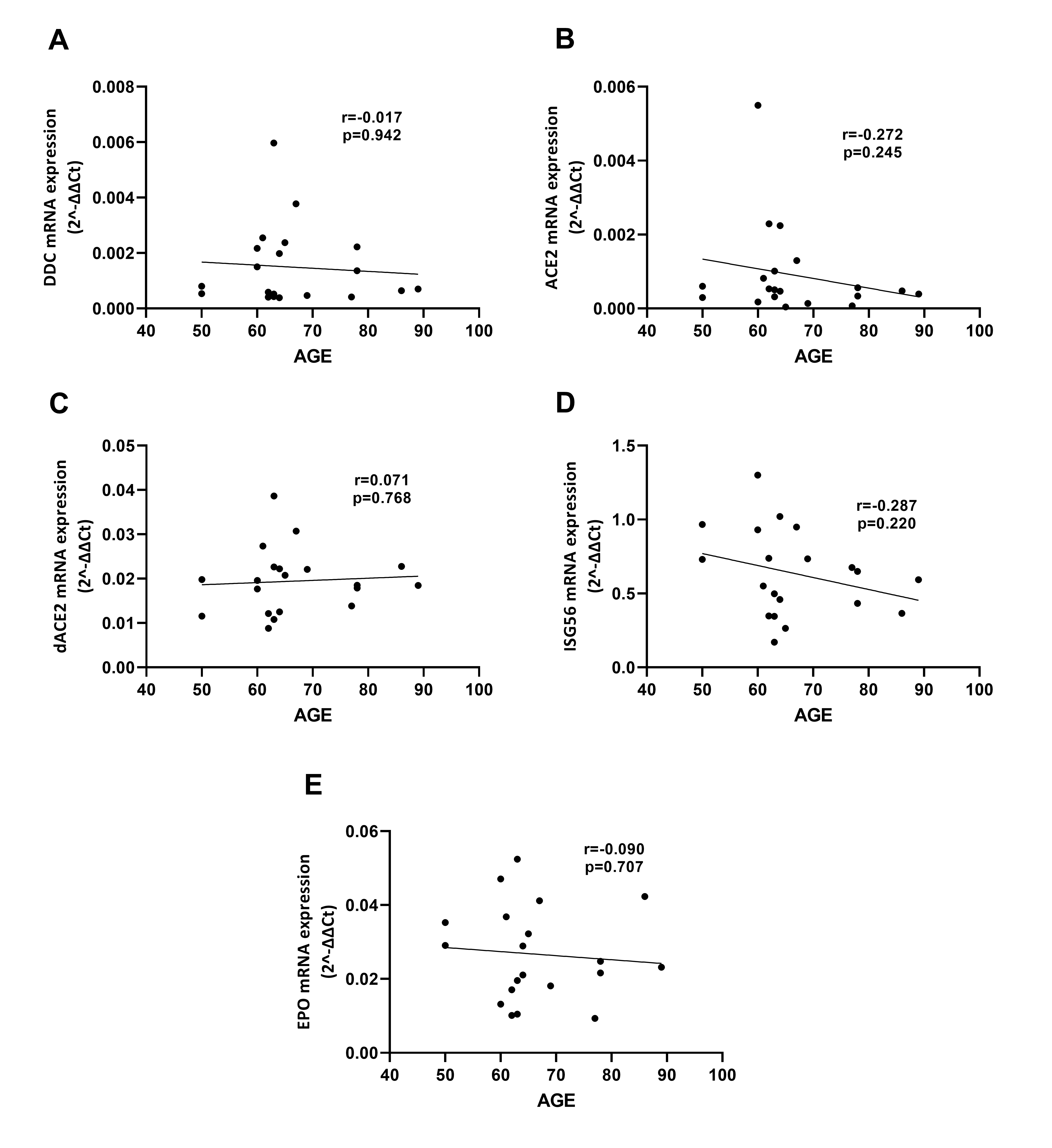

Supplement: Supplementary file 1 [file cells-12-00012-s001.zip › cells-2050602-supplementary/Figure S2.tif]

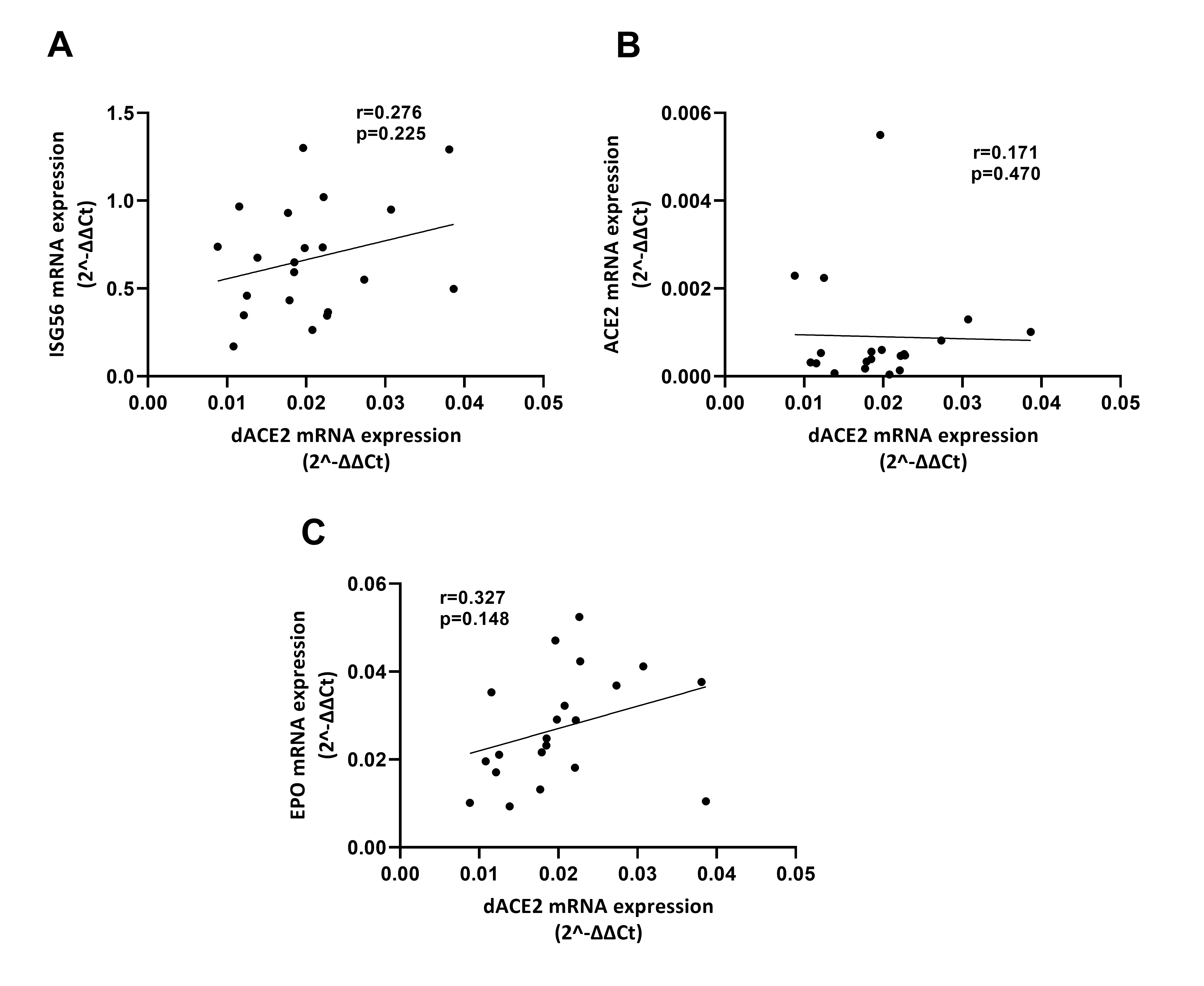

Supplement: Supplementary file 1 [file cells-12-00012-s001.zip › cells-2050602-supplementary/Figure S3.tif]

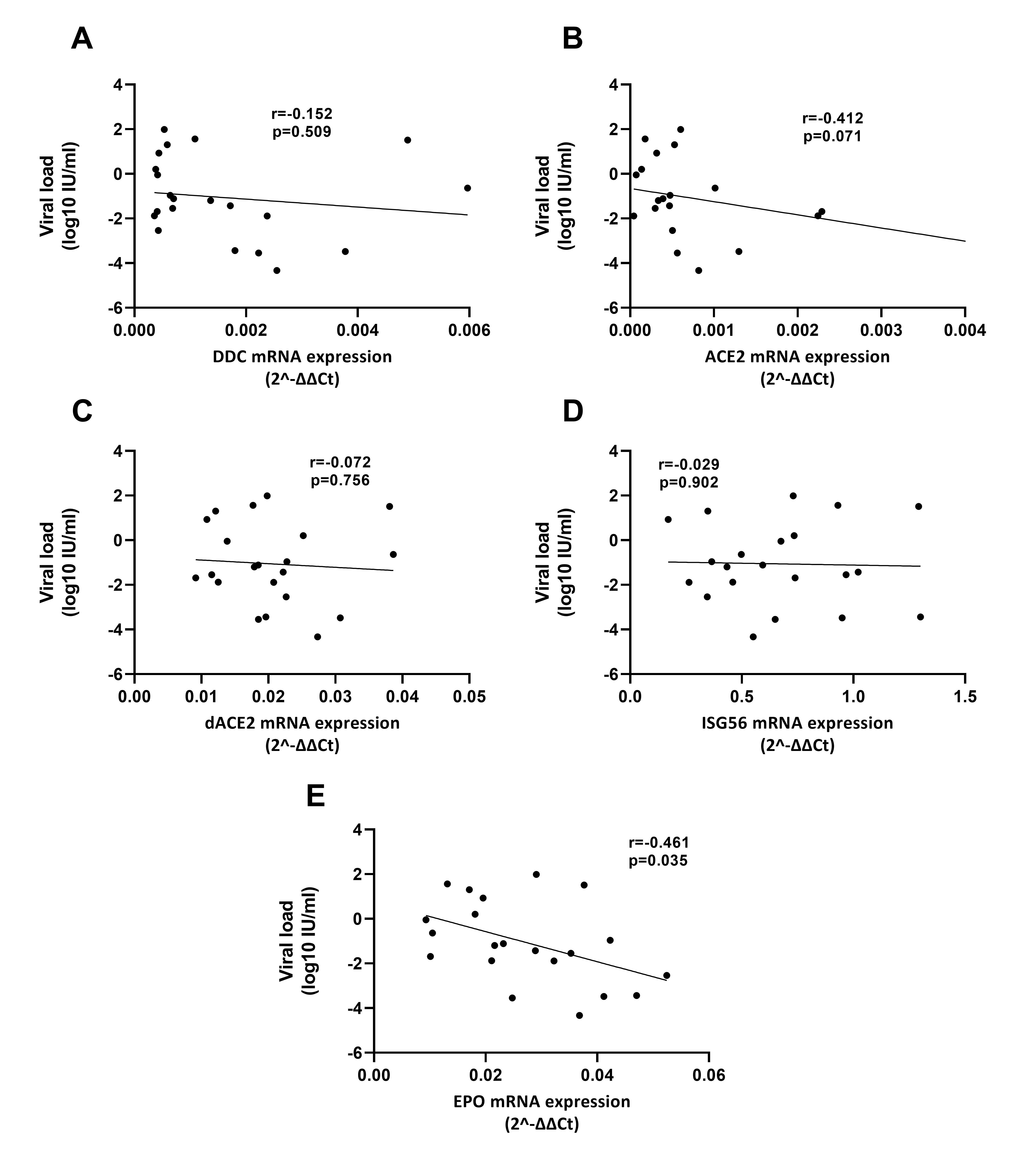

Supplement: Supplementary file 1 [file cells-12-00012-s001.zip › cells-2050602-supplementary/Figure S4.tif]

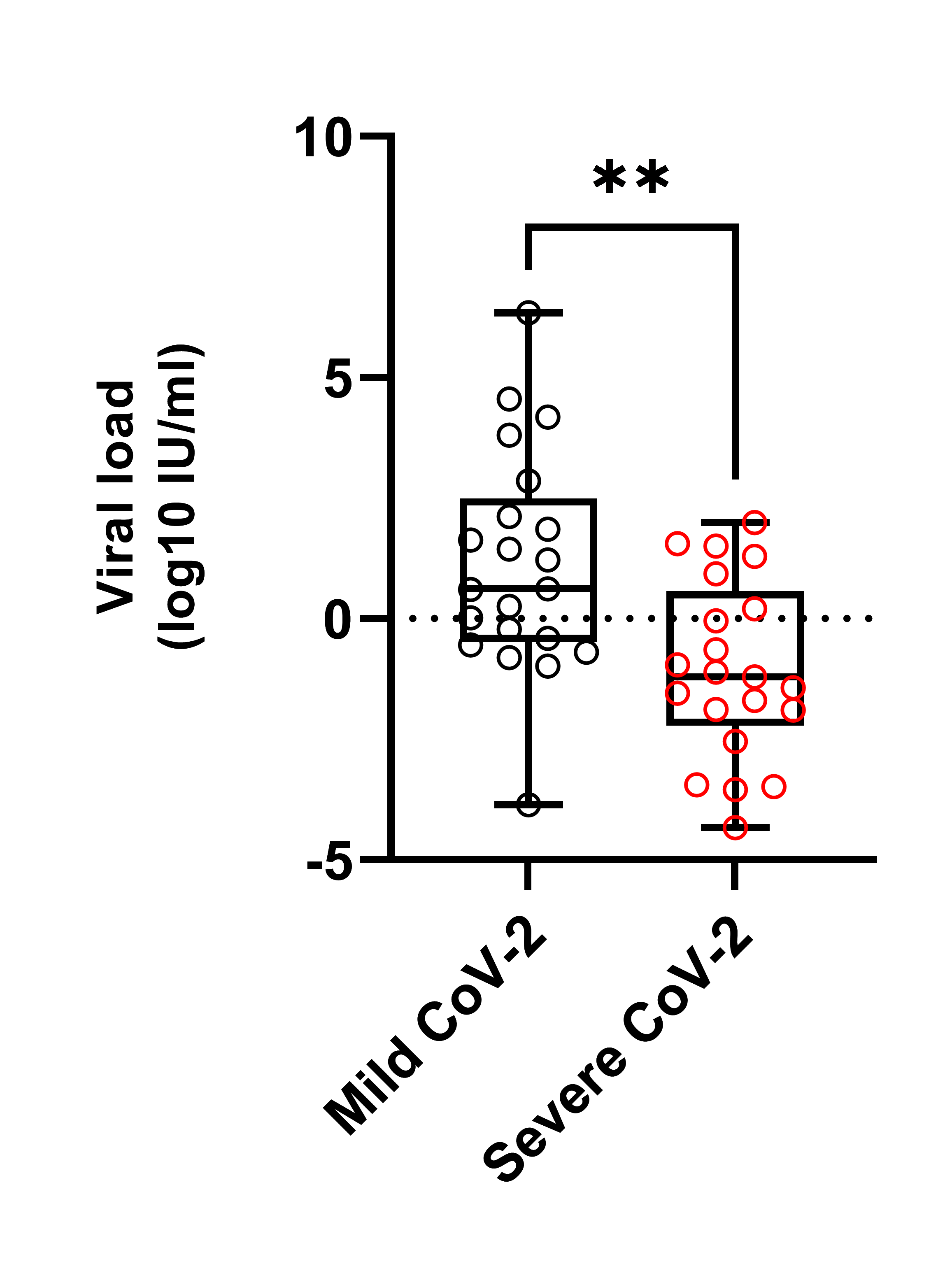

Supplement: Supplementary file 1 [file cells-12-00012-s001.zip › cells-2050602-supplementary/Figure S5.tif]

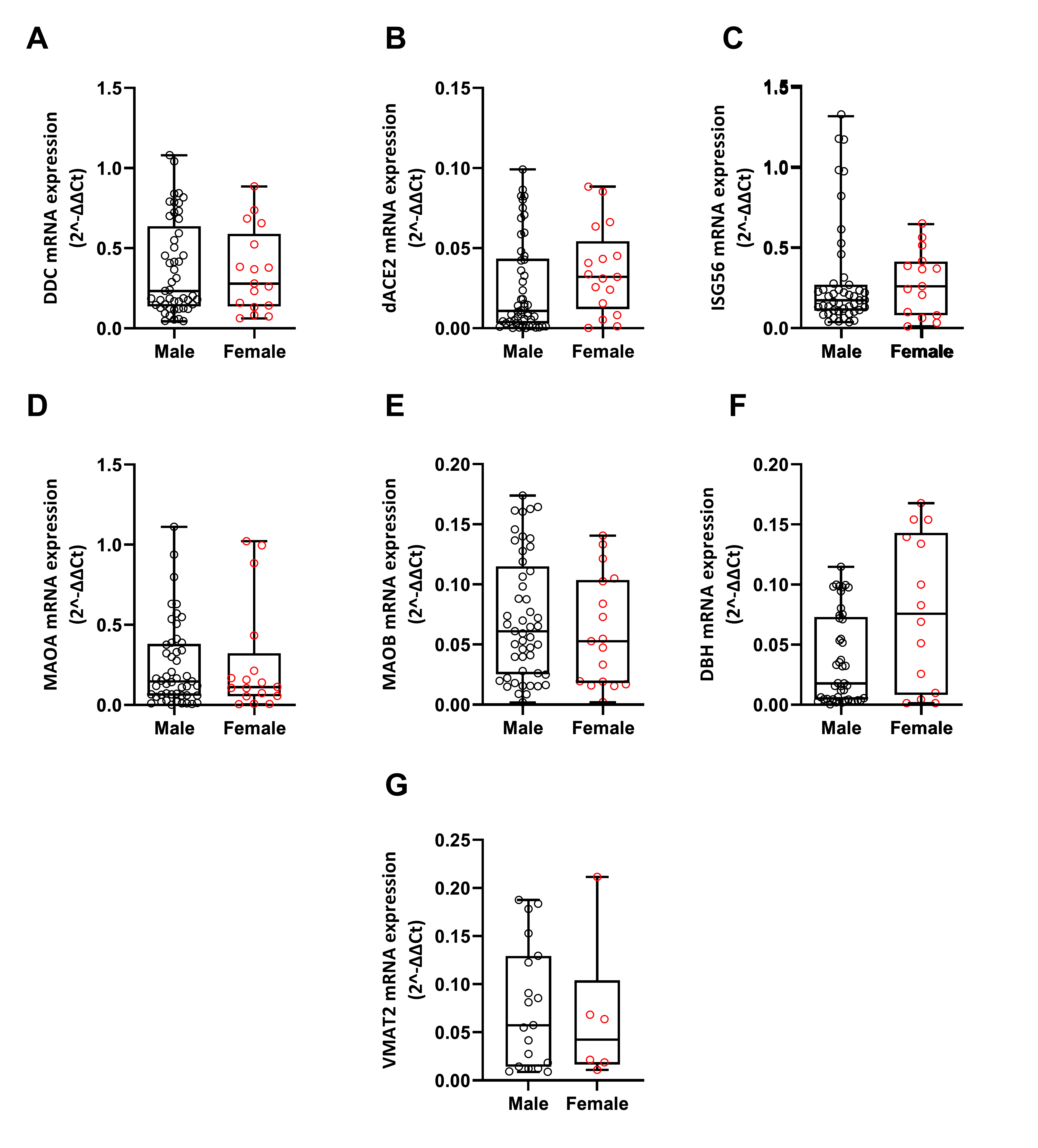

Supplement: Supplementary file 1 [file cells-12-00012-s001.zip › cells-2050602-supplementary/Figure S6.tif]

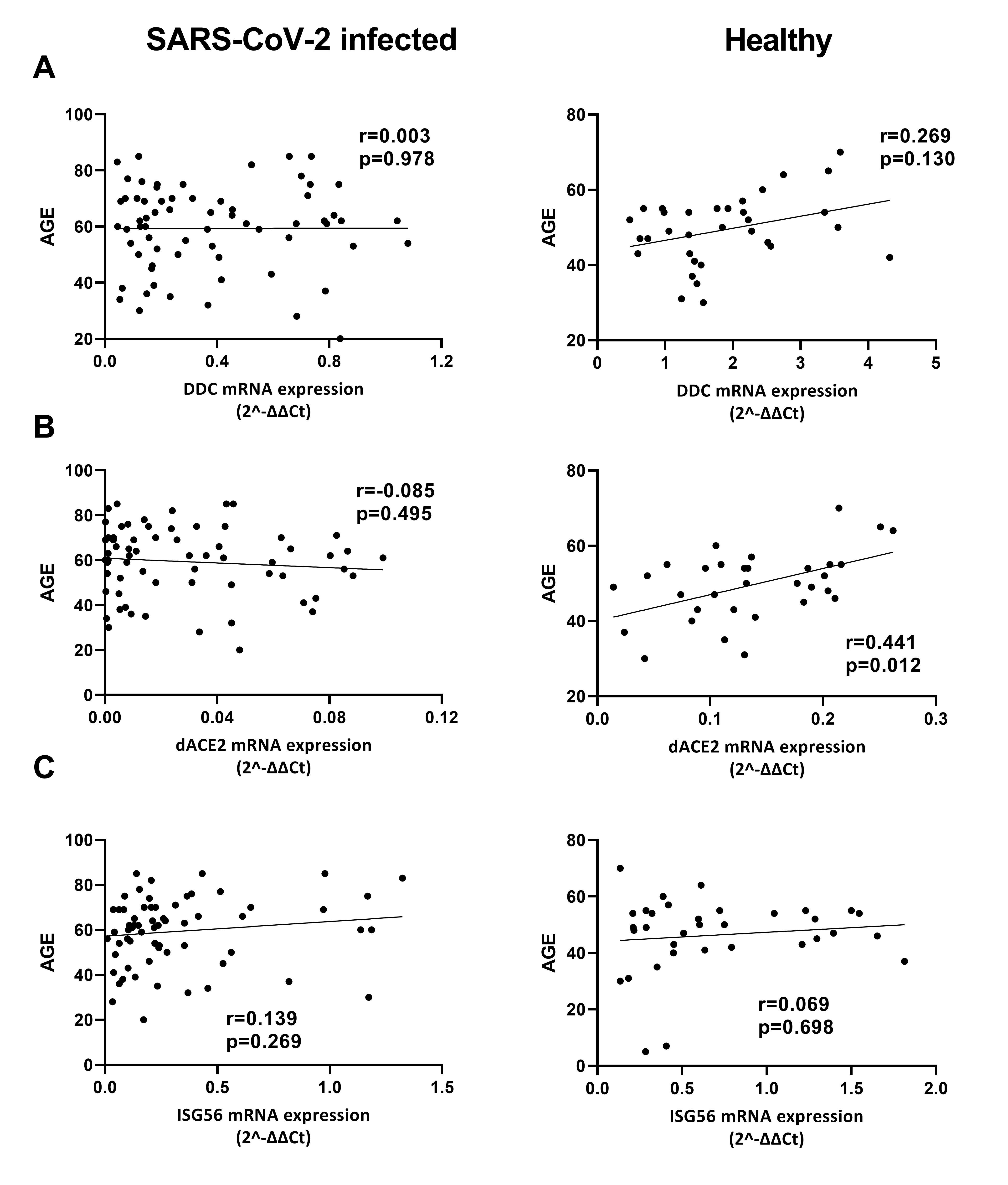

Supplement: Supplementary file 1 [file cells-12-00012-s001.zip › cells-2050602-supplementary/Figure S7.tif]

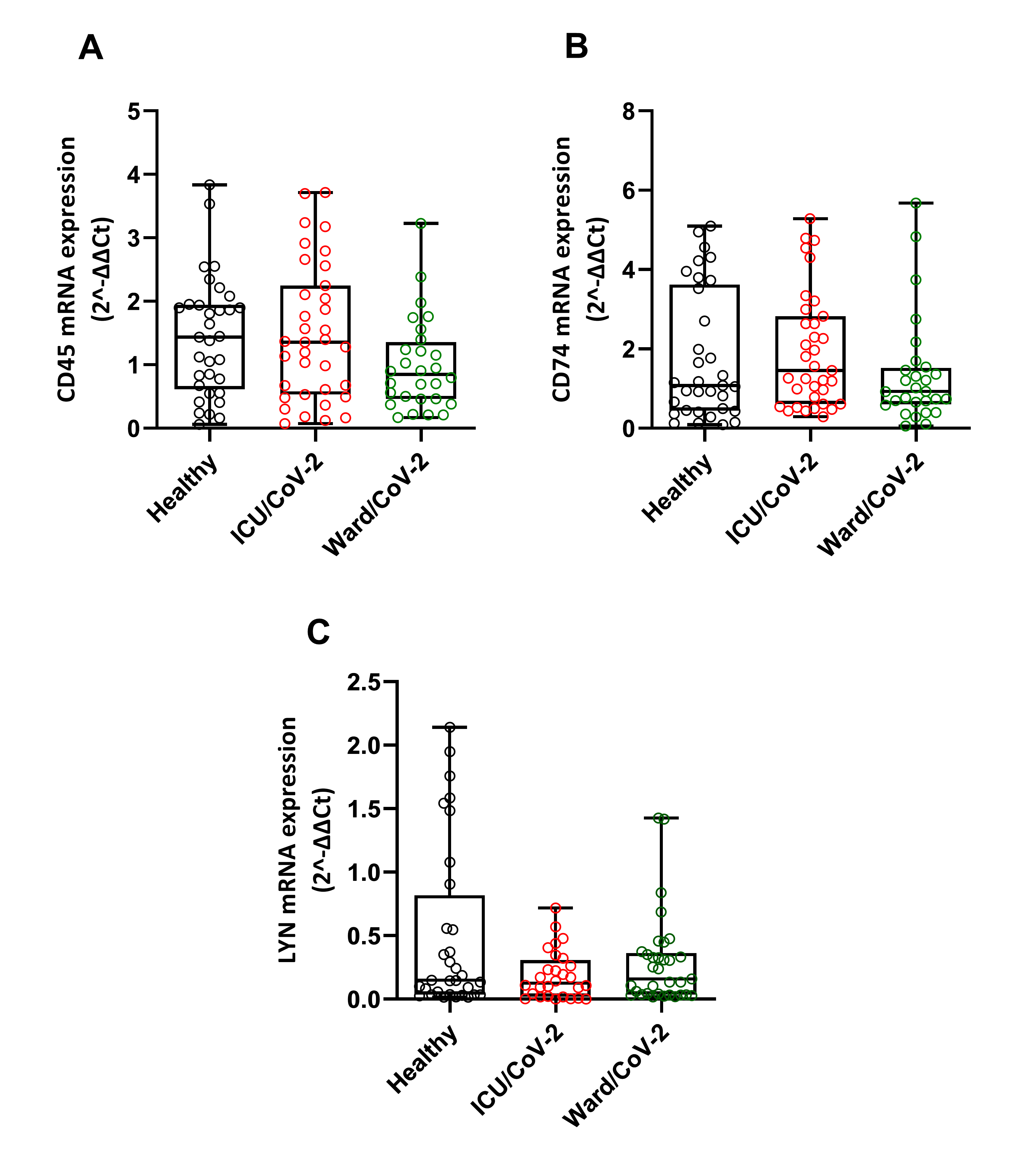

Supplement: Supplementary file 1 [file cells-12-00012-s001.zip › cells-2050602-supplementary/Figure S8.tif]

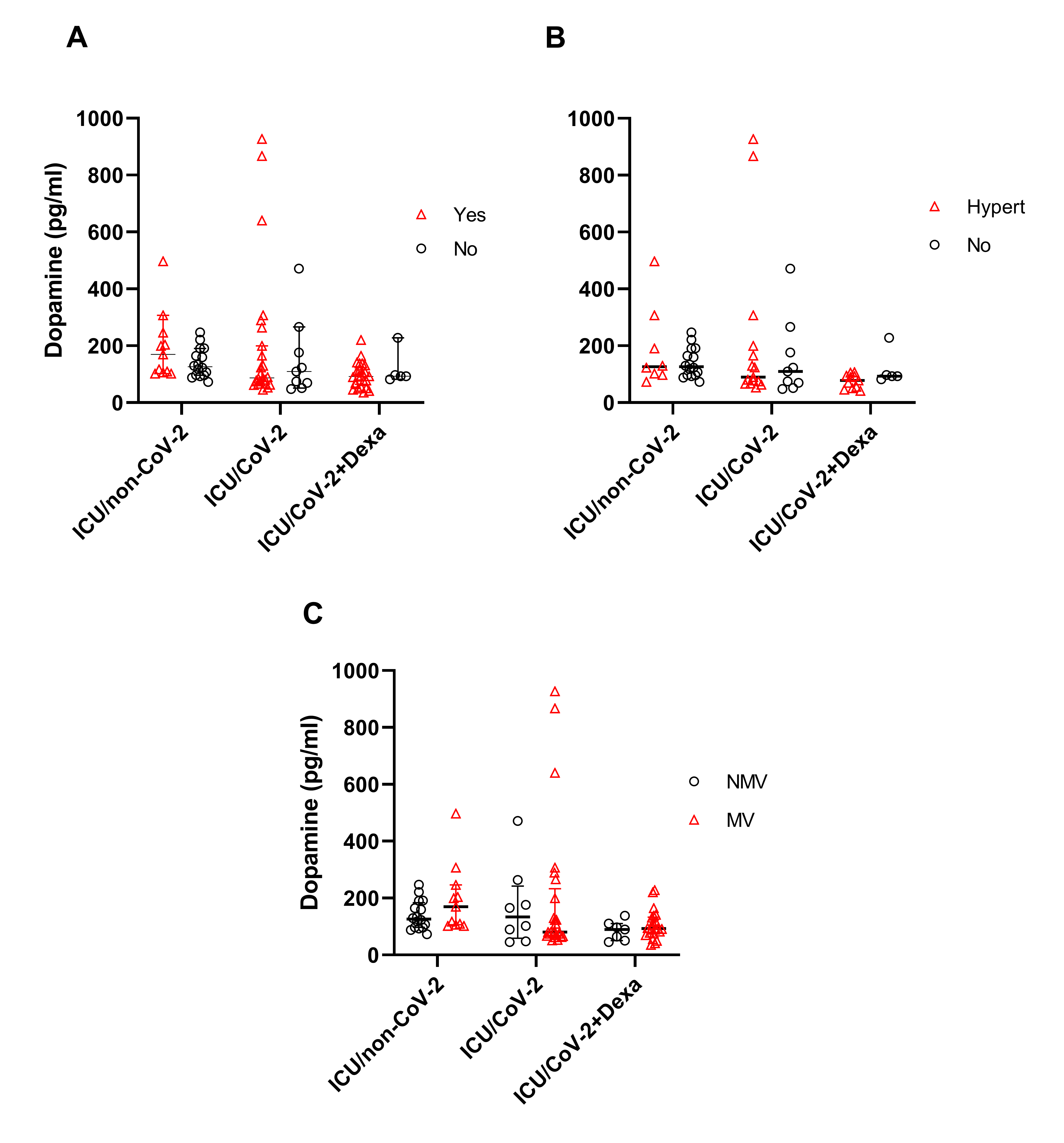

Supplement: Supplementary file 1 [file cells-12-00012-s001.zip › cells-2050602-supplementary/Figure S9.tif]
